# The *Tetraodon nigroviridis* reference transcriptome: developmental transition, length retention and microsynteny of long non-coding RNAs in a compact vertebrate genome

**DOI:** 10.1038/srep33210

**Published:** 2016-09-15

**Authors:** Swaraj Basu, Yavor Hadzhiev, Giuseppe Petrosino, Chirag Nepal, Jochen Gehrig, Olivier Armant, Marco Ferg, Uwe Strahle, Remo Sanges, Ferenc Müller

**Affiliations:** 1 Biology and Evolution of Marine Organisms, Stazione Zoologica Anton Dohrn, Villa Comunale, Naples - 80121, Italy; 2 Institute of Cancer and Genomic Sciences, College of Medical and Dental Sciences, University of Birmingham, Edgbaston, Birmingham, B15 2TT, UK; 3 BRIC - Biotech Research & Innovation Centre, University of Copenhagen, Ole Maaløes Vej 5, DK-2200 Copenhagen N, Denmark; 4 Karlsruhe Institute of Technology (KIT), Campus North, Institute of Toxicology and Genetics (ITG), Hermann-von-Helmholtz-Platz 1, 76344 Eggenstein-Leopoldshafen, Germany

## Abstract

Pufferfish such as fugu and tetraodon carry the smallest genomes among all vertebrates and are ideal for studying genome evolution. However, comparative genomics using these species is hindered by the poor annotation of their genomes. We performed RNA sequencing during key stages of maternal to zygotic transition of *Tetraodon nigroviridis* and report its first developmental transcriptome. We assembled 61,033 transcripts (23,837 loci) representing 80% of the annotated gene models and 3816 novel coding transcripts from 2667 loci. We demonstrate the similarities of gene expression profiles between pufferfish and zebrafish during maternal to zygotic transition and annotated 1120 long non-coding RNAs (lncRNAs) many of which differentially expressed during development. The promoters for 60% of the assembled transcripts result validated by CAGE-seq. Despite the extreme compaction of the tetraodon genome and the dramatic loss of transposons, the length of lncRNA exons remain comparable to that of other vertebrates and a small set of lncRNAs appears enriched for transposable elements suggesting a selective pressure acting on lncRNAs length and composition. Finally, a set of lncRNAs are microsyntenic between teleost and vertebrates, which indicates potential regulatory interactions between lncRNAs and their flanking coding genes. Our work provides a fundamental molecular resource for vertebrate comparative genomics and embryogenesis studies.

Pufferfish species of the Tetraodontidae family such as fugu^1^ and tetraodon^2^ carry the smallest genomes among all vertebrates (350–400 Mb) whose size is about 1/8^th^ of the human genome. Their genomes are thought to be enriched for functional elements because, with respect to other vertebrates, they are characterized by lower percentages of repetitive sequences, shorter introns and intergenic regions, higher gene density and chromosomal stability^2^. Therefore, they were suggested to be ideal models for comparative analysis^3^. The compact nature of these genomes could also aid in the characterization of syntenic relationships and potentially highlight regulatory interactions between *cis* regulatory elements which are distributed over megabases in larger vertebrate genomes. The sequencing of the tetraodon genome permitted the first accurate prediction of the number of human protein coding genes^4^ and allowed the discovery of a teleost-specific whole genome duplication, which occurred in an ancestral teleost species about 300 million years ago^2^. The tetraodon genome is also characterized by significantly higher assembly contiguity compared to the *Takifugu rubribes* genome, thus resulting the ideal pufferfish genome for comparative and evolutionary studies in vertebrates. However, efficient exploitation of these advantages is hindered by the scarcity of experimentally produced transcriptomes leading to an underestimation of its transcriptional output, especially for what concerns long non-coding RNAs (lncRNAs). Indeed, despite numerous studies have demonstrated the widespread transcription of lncRNAs in organisms as diverse as mammals, zebrafish, insects and nematodes^5^,^6^,^7^,^8^, annotations of pufferfish gene models are devoid of them. The cause for this lack is lower expression levels of lncRNAs with respect to protein-coding genes which makes their identification difficult using low and medium scale EST libraries approaches. In addition, their grammar is unknown and therefore we do not have any algorithm to predict them purely from the sequence of a genome using *ab-initio* approaches. Nevertheless, they have fundamental and diverse functional roles in nervous system functioning, development and disease^9^,^10^ even if they are little or no conserved at the sequence level^11^.

The early embryonic transcriptome is of particular interest for gene discovery, as it represents a large proportion of the organism’s transcriptome. We can consider it as the output of two different transcriptomes: the maternal composed of mRNAs expressed by the mother’s genome in the oocyte and the embryonic/zygotic composed of mRNAs expressed by the genome of the embryo in preparation for differentiation and establishment of the different cell types. During the maternal to zygotic transition (MZT), a large proportion of the maternal transcriptome is replaced by the processes of large-scale degradation of maternal mRNAs and the dramatic increase in activation of zygotic genes^12^. LncRNAs are also reported to be expressed in a stage-specific fashion during MZT, which suggests that they play important roles during early embryogenesis^13^. However, the pattern of transcription and gene regulation during embryogenesis in teleosts remains scarcely studied, except for notable examples in zebrafish^14^,^15^,^16^,^17^,^18^.

The gene set of *Tetraodon nigroviridis* relies on *ab-initio* and comparative predictions aided by a set of medium scale ESTs libraries from adult individuals prepared and sequenced for the genome project^2^. This low source of transcriptional evidences is mainly due to the past difficulties of breeding tetraodon in laboratory conditions, which was achieved only recently^19^. Consequently, we reported observation of the early development of tetraodon in a laboratory environment^20^ and here, we present for the first time its developmental transcriptome. We have annotated 3 times as many transcripts (61,033 in 23,837 loci) as available in the Ensembl genome browser and identified ~1100 lncRNAs expressed during embryogenesis. We also report maternally inherited and zygotically initiated transcripts during early embryogenesis and compared them with zebrafish genes which are active in MZT.

## Results

### The early developmental transcriptome of tetraodon gives transcriptional evidence to predicted gene models and expands it to novel coding and non-coding gene loci

To provide transcriptional evidence for the Ensembl annotated tetraodon gene models and catalog its embryonic active genes, we generated more than 700 million (M) RNA-seq reads from three developmental stages: eggs, whole embryo at 30% epiboly (30 epi) and whole embryo at 24 hours post fertilisation (24 hpf). More than 90% of reads from all stages passed the quality filtering and approximately 70% of the filtered reads mapped to the genome (Suppl. Table S1). The majority of the bases covered by the mapped reads fall on the annotated coding exons (75–78%) confirming that a significant percentage of transcription occurs in the protein-coding loci during embryogenesis. However, a sizable fraction of reads (12–15%) also falls in intergenic regions suggesting the existence of previously unannotated embryonic expressed transcripts. The filtering parameters used in the cleaning and mapping of the reads assure us that these transcripts are unlikely to be derived from mapping artifacts (see methods). We can also exclude DNA contamination since the percentage of spliced reads mapping in intergenic regions (37%) is similar to the one mapping on known exons (32%).

**Figure 1:**
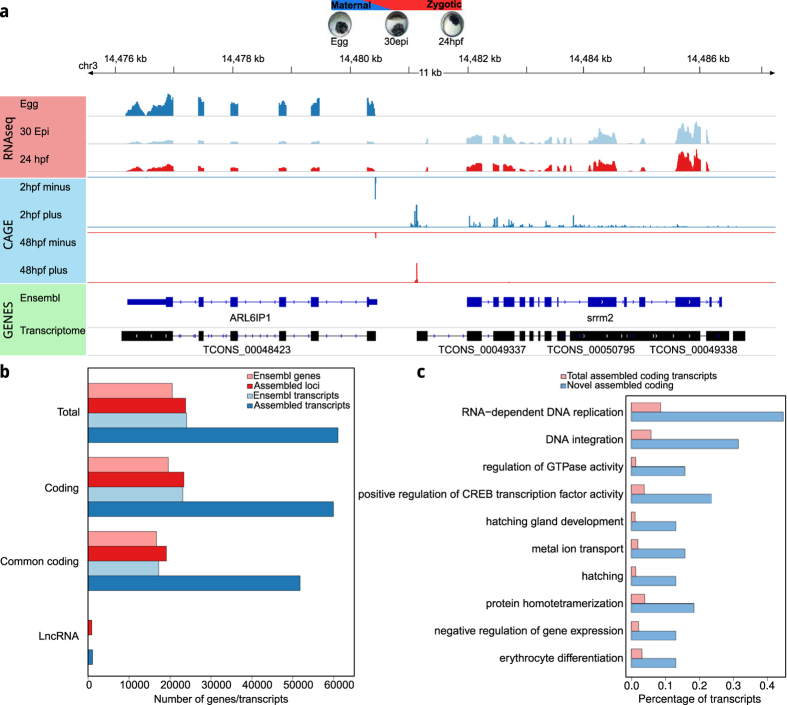
Principal features of the assembled developmental transcriptome of *Tetraodon nigroviridis.* (**a)** Coverage plot from a representative 12 KB region on the tetraodon genome showing data from RNAseq of the three developmental stages, CAGE transcriptional start site peaks, Ensembl genes and assembled transcript models. (**b)** Comparison of the number of assembled loci and transcripts against the existing gene models in Ensembl. *Common coding* refers to assembled loci and transcripts mapped to a known Ensembl transcript model. (**c)** GO enrichment of the novel discovered coding transcripts. The novel coding transcripts are enriched in GO terms deemed important for early embryo development. The x-axis indicates the percentage of transcripts which are associated to a particular GO biological process while the y-axis reports the significantly enriched GO classes.

The mapped reads were assembled into 61,033 transcript models falling under 23,837 loci (minimum length cut-off 200 nucleotides) (Fig. 1a,b and Suppl. Data S1,2). The assembled transcripts were functionally and compositionally annotated using Annocript^21^ measuring also their non-coding potential scores (NCP) using Portrait^22^. The annotations were then used to stringently classify *candidate lncRNAs*. We classified transcripts lacking any homology to coding sequences and/or protein domains and showing ORFs shorter than 100 aminoacids as *candidate lncRNAs*. We then evaluated the distribution of NCP for all the transcripts (Suppl. Fig. S1a) and chose the average NCP of all *candidate lncRNAs* (0.76) as a conservative cut-off to predict *potential lncRNAs* (Suppl. Fig. S1b and Suppl. Data S3–5). Next, we filtered out all *potential lncRNAs* proximal to 3′UTRs, because they might represent alternative polyadenylation events of coding genes to finally classify 1120 transcripts (918 loci) as lncRNAs (Suppl. Data S6). The different additive filtering strategies and the chosen cut-offs make us confident that the identified final set of lncRNAs contains few false positives, while the total number of lncRNAs in tetraodon is probably under-estimated (see methods). The remaining 59,913 transcripts from 23,390 loci were classified as *coding*. Among them, the assembled coding transcripts mapping to an Ensembl coding gene were classified as *common coding* while those not mapping on an Ensembl gene model and/or an ESTs from GenBank were classified as *novel*.

We checked the overlap of the assembled transcripts with Ensembl gene models by location-based mapping thereby transcripts classified as coding were used, since the Ensembl annotated genes for tetraodon lack lncRNAs. The majority of the Ensembl gene models were mapped to an assembled locus (85%) indicating a broad agreement between the two datasets. The majority (65%) of our assembled coding loci are longer than their Ensembl counterpart (Suppl. Fig. S2a), leading to the extension of both the 3′ and 5′ ends of the Ensembl genes (about 48% of mapped Ensembl genes), while about 8% extend only at the 3′ ends and another 8% extend only at the 5′ ends. Interestingly, transcriptional start sites (TSSs) for 57% of coding and 85% of lncRNAs are supported by CAGE-seq data generated from early developmental stages (2 hpf and 48 hpf)^23^. Tetraodon lncRNAs result to be shorter with fewer but longer exons in comparison to coding transcripts. In addition, 14% of them (161 transcripts) have a single exon and do not show any splicing evidence (Suppl. Fig. S2b–d). They also show lower expression levels compared to coding transcripts (Suppl. Fig. S3). These observations were reported also for lncRNAs in all other vertebrate species analyzed^6^,^8^.

The assembled gene models provide transcriptional evidence to 2667 (3816 transcripts) novel coding loci the majority of which (67%, 1671 loci, 2557 transcripts) are conserved with other annotated teleosts genes while a smaller fraction is conserved among vertebrates (43%, 1049 loci, 1646 transcripts). The remaining 32% of the novel loci (1033 loci, 1213 transcripts) have no known homolog in other species, and might therefore represent transcripts specific to *tetraodontiformes*. However, these genes are currently undetected in fugu probably due to the scarcity of experimentally generated transcriptomes and the fragmented nature of the fugu genome. Functional analysis of the novel coding loci resulted in significant enrichments for biological processes related to development such as *RNA dependent DNA replication, regulation of CREB transcription factor and GTPase*, and *hatching gland development* (Fig. 1c). However, a contribution to the enrichment for *RNA dependent DNA replication* and *DNA integration* terms might result from the annotation of transposon related sequences that were masked during the initial reference genome annotation.

The transcriptome herein assembled provides experimental evidences for the predicted gene models, identifies novel coding loci and the active transcription of lncRNAs during early embryogenesis representing an important resource to improve the current genome annotation in tetraodon.

### The retention of exon lengths in a compact genome indicates the conservation effects of a selective pressure on lncRNAs across vertebrates

**Figure 2:**
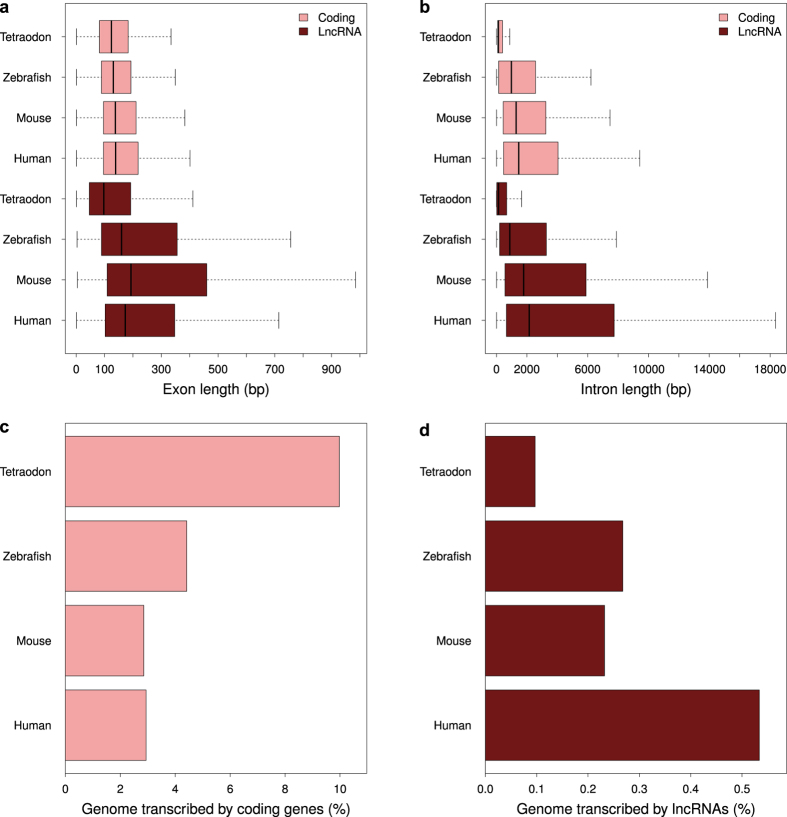
lncRNA features across vertebrate genomes. (**a)** Distribution of coding and lncRNA exon size across vertebrates. (**b)** Distribution of coding and lncRNA intron size across vertebrates. (**c)** Coding transcribed fraction of vertebrate genomes. (**d)** Non-coding transcribed fraction in vertebrate genomes.

Reduction in intron size, low rates of transposition and scarcity of non-coding genes are the mechanisms reported to underlie the drastic reduction in genome size of Tetraodontiformes^2^,^3^,^24^. With the availability of high quality annotated coding and lncRNA genes, we decided to analyse how the compaction of the tetraodon genome has affected the length of exons and introns. The impact of genome size reduction is best observed within the introns of protein coding genes, which are significantly smaller in pufferfishes as compared to other vertebrates, while their exon lengths remained comparable. Remarkably, in our analysis, tetraodon lncRNA transcripts also showed a dramatic reduction in introns length with respect to other vertebrates, while the lengths of their exons remained comparable (Fig. 2a,b). It is important to note, that the reduction in genome size led us to observe an increase in the genomic fraction harbouring coding RNAs (Fig. 2c) but not lncRNAs (Fig. 2d). This can be explained by the reduced discovery rate caused by lower transcriptional levels of lncRNAs and by our stringent classification criteria, which might have affected the overall estimation of the non-coding transcriptome. Similarly to other species, further sequencing efforts of additional tissues and stages will provide a more comprehensive lncRNA catalogue also for tetraodon. However, the very limited reduction of lncRNA exon lengths (compared to introns) in such a compact genome is surprising. LncRNA sequences evolve faster compared to coding genes because they lack evolutionary pressure to retain codon structure^25^ and therefore the retention of their lengths represents evidence suggesting specific functional and evolutionary constraints. We propose that although the compact genome of tetraodon is expected to be accompanied by a loss of repetitive and non-coding elements, the transcription and processing of lncRNAs exons must remain unaltered to maintain their functionality at least during embryogenesis.

### Association of lncRNAs and coding genes with transposable elements (TEs)

**Figure 3:**
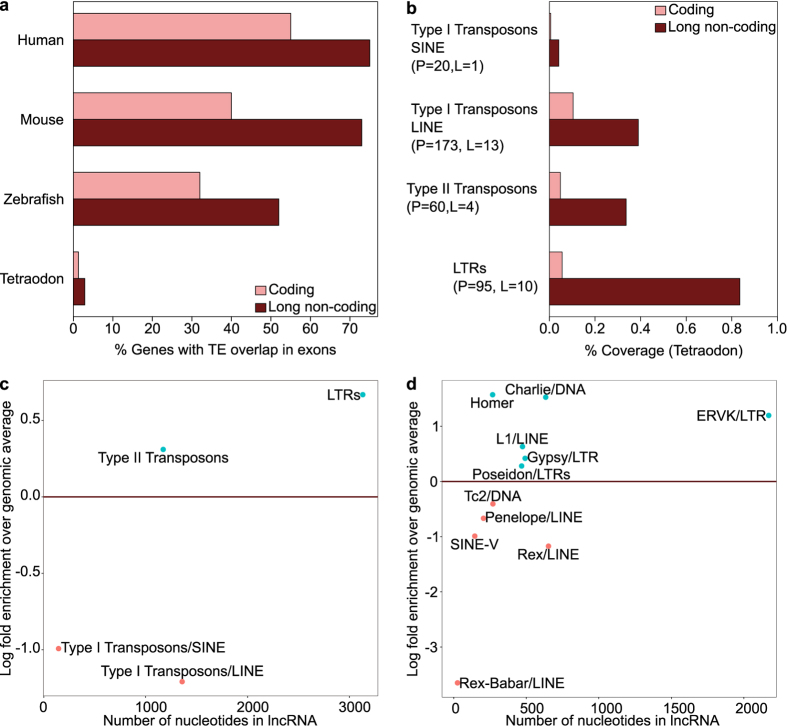
Association of lncRNAs with transposable elements (TEs). (**a)** Percentage of coding and lncRNA genes containing an exonic TE fragment in human, mouse, zebrafish and tetraodon. (**b)** Coverage of different classes of TEs in transcripts of tetraodon. (**c**,**d**) fraction of the transcriptome containing sequences from specific TE (**c**) classes and (**d**) families with respect to the respective TEs genomic averages. Larger families are to the right. On the y-axis, enrichments are above zero and depletions are below zero. ERVK families are particularly enriched. P and L indicate respectively the number of coding and lncRNA loci associated with a particular family of TEs.

The majority of human long intergenic non-coding RNA (lincRNA) exons (80%), unlike protein-coding sequences, contain fragments derived from transposable elements (TEs). These appear to be enriched for elements belonging to the long terminal repeats (LTRs) class of the human endogenous retrovirus (HERV) family. The enrichment is strongest at lincRNAs transcriptional start sites (TSSs) and suggests the functional involvement of HERVs in the regulation of lincRNAs expression. Similar enrichments have also been observed in mouse, where the ERV1 family of LTR is enriched in lincRNAs while ERVK appear to be mainly associated with TSSs^26^. A diversity of TEs might be enriched in lncRNAs in different vertebrate species, for example in zebrafish lncRNAs there is an enrichment for DNA transposons (type II transposons), which reflect their unique genomic expansion in this species. These observations suggest a role for TEs in lncRNAs evolution^27^. We compared the association of major TE classes to lncRNAs in tetraodon, human, mouse, and zebrafish. The analyzed classes are: (1) long interspersed elements (LINE), (2) short interspersed elements (SINE), (3) Type II transposons also known as DNA transposons and (4) long terminal repeats (LTRs). In contrast to other vertebrates, we find a general decoupling of both coding and lncRNAs from TEs (Fig. 3a), which reflects the overall lower frequency of TEs at the level of the whole genome. Nonetheless, a higher fraction of lncRNAs (3%, 27 loci) contains TE fragments in their exons with respect to coding loci (1.3%, 325 loci). In addition, tetraodon lncRNAs display a higher content of TEs in comparison to coding transcripts (Fig. 3b). LTR represents the class with the highest coverage in lncRNAs over protein-coding transcripts. Fragments derived from LTRs are present in 95 coding transcripts and 10 lncRNAs and LTRs are the only class of TEs overlapping with TSSs (33 coding and 6 lncRNAs contain an LTR at their TSS) in addition to a single coding transcript containing a LINE fragment at its TSS. Similarly to analysis performed in human and mouse^26^, we calculated the ratio between the fraction of the genome and the lncRNAs overlapping with specific TE fragments. Our results show that LTR elements, specifically those belonging to the ERVK family, cover a higher fraction of lncRNAs with respect to the genomic average (Fig. 3c,d). This difference is not statistically significant due to the low number of lncRNAs containing LTRs. Nevertheless, this result is in agreement with what has been observed in mammals, where LTRs are enriched in lncRNAs with respect to the genomic average and suggest a potential regulatory role. These results suggest that, unlike other vertebrates, the majority of currently identified tetraodon lncRNAs have probably evolved independently of TEs. However, despite a drastic loss of TEs content in the tetraodon genome, some lncRNAs show a detectable enrichment of TEs mirroring the same TEs content and localization features observed in other vertebrate species^27^ and therefore supporting the potential role of TEs in the evolution and transcriptional regulation of at least a small set of tetraodon lncRNAs.

### Tetraodon maternal and embryonic transcripts are associated with early embryonic developmental functions

**Figure 4:**
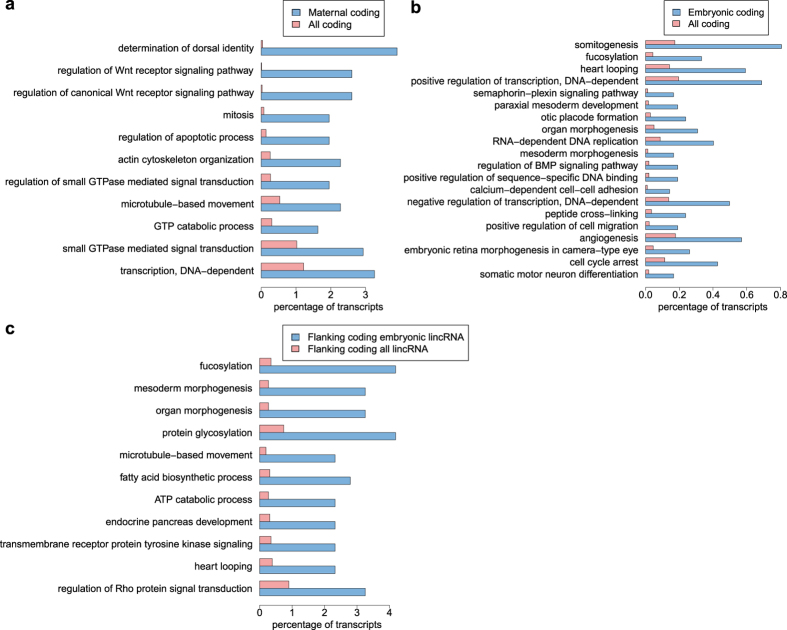
Functional association of coding and lincRNA transcripts with maternal and embryonic specific expression in tetraodon. GO enrichment analysis of tetraodon (**a**) maternal coding genes, (**b**) embryonic coding genes, (**c**) coding genes proximal to embryonic lincRNAs. The x-axis indicates the percentage of transcripts associated to a particular GO biological process while on the y-axis are reported the significantly enriched GO classes.

In order to identify maternal and embryonic expressed transcripts, we evaluated the expression levels in the three developmental sampled stages (Suppl. Data S7) and carried out pairwise comparisons between each combination of two stages. To limit the rate of false positives, which might arise due to the lack of replicates we used stringent parameters and a specific filtering strategy in the selection of differentially expressed transcripts (see methods). A total of 10,965 coding and 192 lncRNA transcripts showed a significant change in the expression levels in at least one of the comparison. Among them we identified and classified the groups of maternal (307 coding, 10 lncRNAs) and embryonic (4221 coding, 81 lncRNAs) specific transcripts (see methods, Table 1 and Suppl. Table S2 and Suppl. Data S8). To understand the functions of coding genes differentially expressed during early development in tetraodon, we performed a gene ontology (GO) enrichment analysis (Suppl. Table S3). Maternal-specific protein coding genes showed significant enrichment for GO classes such as *determination of dorsal identity, regulation of WNT signalling pathway, actin cytoskeleton organisation* and *mitosis* (Fig. 4a) in agreement with what has been observed for maternally deposited transcripts in other species^28^,^29^. The GO processes of *BMP signalling pathway* and *somitogenesis* were enriched in embryonic expressed genes along with *calcium-dependent cell-cell adhesion, organ morphogenesis* and *regulation of transcription*, terms usually associated with the developing embryo (Fig. 4b).

It has been suggested that lincRNAs are functionally associated and often co-expressed with their proximal coding genes^11^. We performed correlation analysis comparing the fold changes of lncRNAs transcripts with respect to the fold changes of their respective proximal coding genes. Results show a significant positive correlation (rho = 0.32, p-value = 1.34e10^−78^) indicating that lncRNAs present expression dynamics similar to those of their flanking coding genes. Hence, to identify the putative functions of developmentally regulated lincRNAs, we analysed the functional enrichments of coding genes proximal to embryonic specific lincRNAs with respect to coding genes proximal to the whole set of lincRNAs (10 KB upstream and downstream). The number of maternal lincRNAs was too low (10 transcripts) to perform a similar analysis on them. Remarkably, the terms significantly enriched among coding transcripts proximal to embryonic lincRNAs include processes related to development, signalling, organogenesis and differentiation (Fig. 4c). Our results highlight the importance of the assembled transcriptome as an important resource giving novel insights into the transcriptional dynamics and functions of protein-coding and non-coding transcripts during embryogenesis.

### Expression of tetraodon maternal and zygotic specific genes shows similarity to their zebrafish orthologs indicating conservation of transcriptional dynamics during the MZT in teleosts

**Figure 5:**
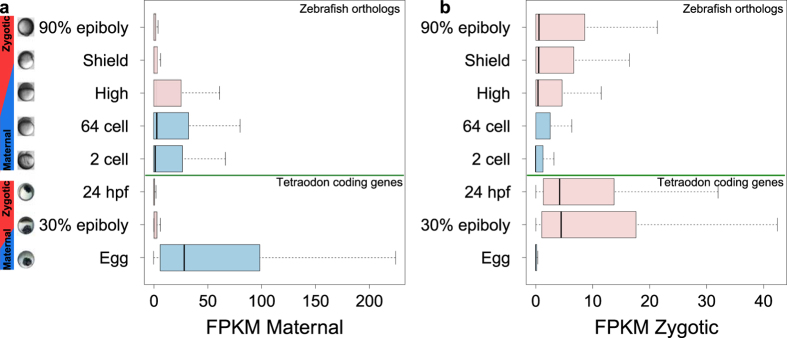
Transcriptional abundance during development in tetraodon maternal and embryonic specific genes and their orthologs in zebrafish. Distributions of expression levels for tetraodon maternal and embryonic genes and their zebrafish orthologs during different developmental stages. (**a**) Expression of maternal specific genes. (**b**) Expression of embryonic specific genes.

To estimate the relationships between developmental gene expression dynamics of zebrafish and tetraodon, we compared the expression levels of tetraodon maternal and embryonic coding genes with their respective orthologs in zebrafish^15^. More than 75% maternal and embryonic specific loci map to a corresponding Ensembl gene model. Among them ~85% have a corresponding ortholog in zebrafish. It is important to note that, due to the lack of developmental transcriptomic data from equivalent stages between zebrafish and tetraodon, the comparisons shown in Fig. 5a,b are restricted to the maternal stages (egg for tetraodon versus 2–64 cell stage for zebrafish) and the zygotic genome activation (30% epiboly for tetraodon versus high and shield for zebrafish). We also kept in account that tetraodon 24 hpf stage is morphologically comparable to the early stages of somitogenesis in zebrafish (10.5 hpf)^20^. Tetraodon maternal genes are predominantly expressed in the eggs with almost no detectable expression in 30% epiboly and 24 hpf (Fig. 5a). The expression pattern of their respective zebrafish orthologs show a similar dynamics and is in support of their maternal origin also in this species. Indeed, in zebrafish their expression results restricted to 2 cell, 64 cell and high stages followed by a rapid decrease during shield and 90% epiboly. Similar observations can be made for the zebrafish orthologs of the tetraodon embryonic genes (Fig. 5b). In our tetraodon data, the transcription of embryonic genes start at 30% epiboly and their zebrafish orthologs appear to be minimally expressed in 2 cell and 64 cell stage followed by a steep rise of expression abundance in the embryonic stages of high, shield and 90% epiboly (Fig. 5b, Suppl. Table S2). These results suggest that zebrafish orthologs of tetraodon maternal and embryonic genes show a broadly similar expression pattern implying similar functional roles during early embryonic development. Moreover, zebrafish specific genes previously reported to be of maternal and embryonic origin from two different studies^14^,^15^, when compared to their respective maternal and embryonic tetraodon orthologs, did not reveal a significant overlap (Suppl. Fig. S4). These results might be consistent with a recent report showing that developmentally earliest transcribed genes differ among various species^30^. Thus, the observed lack of significant overlaps between zebrafish and tetraodon maternal/embryonic gene sets, together with the maintenance of similar expression dynamics of the transcriptome as a whole might indicate the existence of a broad conserved transcriptional program in which a subset of genes differ between species. However, we observed a similar lack of overlap also when we compared the genes classified as maternal and embryonic in the two different zebrafish studies considered^14^,^15^ (Suppl. Fig. S4). Therefore, we cannot rule out the possibility that different experiments might identify incomplete sets of genes and thus cause the lack of substantial overlaps between them. Deeper and more detailed comparative transcriptomic studies are required to specifically answer this question.

### Conservation of tetraodon lncRNAs among vertebrates

The retention of exon size and the enrichment of TEs in tetraodon lncRNAs encouraged us to further analyse the degree of conservation of tetraodon lncRNAs with other vertebrates. We compared the genomic locations of the tetraodon coding and non-coding transcripts with whole genome alignments of 8 vertebrate species (Multiz8way, human, mouse, medaka, stickleback, fugu, tetraodon, clawed frog). In agreement with previous reports in other species^8^,^25^, tetraodon lncRNAs show a lower level of sequence conservation than coding transcripts but are marginally better conserved than random intergenic regions (Suppl. Fig. S5). In addition, lncRNA and lincRNA transcripts show a similar degree of conservation pattern.

**Figure 6:**
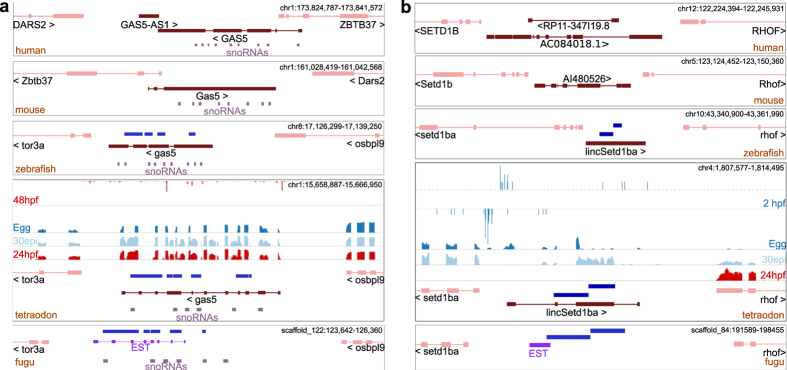
Conservation of tetraodon lncRNAs across vertebrates with specific examples of conservation of sequence and genomic organization. (**a**) Conservation of sequence in the *gas5* lincRNA in human, mouse, zebrafish, tetraodon and fugu. (**b**) Conservation of sequence in the lincRNA_*setd1b* in human, mouse, zebrafish, tetraodon and fugu. The tracks Egg, 30epi and 24hpf represent the RNAseq coverage depth while 2hpf and 48hpf represents CAGE peaks in tetraodon. The coding genes are marked in light red while the lincRNAs are marked in dark red. Blue boxes indicate conserved sequence fragments.

We then searched for lincRNAs showing sequence conservation with annotated lincRNAs in human, mouse, zebrafish and tetraodon. We analyzed only lincRNAs to avoid any potential bias caused by lncRNAs overlapping coding genes. This analysis provided only five lincRNA loci conserved at the exon level between tetraodon and zebrafish that we classified as teleost conserved (Suppl. Table S4). No one of them resulted conserved with mammalian lincRNA exons. The fraction of lincRNAs conserved between zebrafish and tetraodon is significantly higher than what expected by chance (p-value = 2e10^−4^). In addition, following manual curation, we added two transcripts to this list that were filtered out by the initial stringent filters applied to the group of *candidate lncRNAs*. This addition is based on evidences showing that the conserved regions are not originating from a coding gene and that these two *candidate lncRNAs* constitute transcriptional units independent from the flanking coding genes. The first identified transcript corresponds to the tetraodon ortholog of the zebrafish *gas5* lincRNA (Fig. 6a). Tetraodon *gas5* shows conservation at exonic as well as intronic level with its zebrafish ortholog, while with mammals only the intronic regions are highly conserved due to the presence of several host snoRNAs^31^. *Gas5* is involved independently in two functions: regulator of growth and apoptosis and host of snoRNAs^32^,^33^. We detected 10 aligned blocks between the zebrafish and tetraodon *gas5* exons (mean aligned length 190 bp; mean percentage identity 56%). SnoRNAs are highly conserved across diverse eukaryotes, suggesting that the aligned blocks might represent snoRNA sequences overlapping lncRNA exons^34^. However, the majority of conserved blocks (9 out of 10) show no or marginal overlap with snoRNAs indicating that the exonic conservation is independent from snoRNAs in fishes. At the genomic level, we noticed blocks of sequence conservation also with fugu, which support transcription in the homologous locus also in this species. In addition, zebrafish, tetraodon and fugu *gas5* loci are conserved also at the microsyntenic level, where the microsynteny does not extend to mammals (*zbtb37* and *dars2* flanking genes in mammals; *tor3a* and *osbpl9* in fishes). The *gas5* example shows sequence conservation for the *gas5* non-coding gene exons in teleosts along with conservation of local gene order, both of which are lost in mammals. The second manually annotated conserved lincRNA is *lnc_setd1b* lying upstream to the *setd1b* coding gene (Fig. 6b; Suppl. Table S4). *Setd1b* is a histone 3 lysine 4 methyltransferase, which facilitates H3K4 mono, di or tri methylation by regulation of its *SET1* domain^35^. In contrast to the *setd1b* gene the highly conserved paralog *setd1a* does not show the presence of a proximal lncRNA in mouse, zebrafish and tetraodon. We also noticed that the *lnc_setd1b* and the *setd1b* genes show clear and distinct transcriptional initiation signals during early developmental stage of 2hpf in tetraodon CAGE-seq data, an observation, which also holds true during early development in zebrafish and in human cell lines evidenced by previously published CAGE-seq data^23^,^36^. These results suggest that *lnc_setd1b* is an independent transcriptional unit, rather than an alternative polyadenylated transcript of *setd1b* or the downstream *rhof* gene. The transcript not only shows sequence conservation in teleosts (5 conserved blocks; mean aligned length 511 bp; mean percentage identity 52%) but also maintains microsynteny, retaining the same flanking genes and orientations in all the vertebrates species considered.

Finally, in order to negate the effect of lincRNA sequence divergence over large evolutionary distances, we decided to analyse the conservation of lincRNAs based exclusively on microsynteny. The analysis was done using a custom lincRNA microsynteny detection pipeline (SynLinc, code available at http://bit.ly/217nbdV). The pipeline was used to identify lincRNAs between pair of species sharing at least one orthologous flanking coding gene. We found that a substantial number of lincRNAs retains orthologs proximal coding genes when comparing pairs of species (number of syntenic lincRNAs pairs regardless of the orientation, human/zebrafish: 2048/929, mouse/zebrafish: 475/459, zebrafish/tetraodon: 192/165). A recent report counts ~1000 human lincRNAs retaining position and orientation synteny in zebrafish^37^. Considering also the conservation of the relative orientation, in our analysis, we found 790 human lincRNAs to be syntenic with 540 zebrafish lincRNAs, which is comparable to the results from Hezroni *et al*. Finally, 38 tetraodon lincRNA transcripts from 28 loci show conservation of microsynteny across all the species considered (tetraodon, human, mouse and zebrafish), and are classified as vertebrate microsyntenic lincRNAs (Suppl. Table S5, 6). The fraction of lncRNAs found to be microsyntenic in all the species analysed is significantly higher than what can be expected by chance based on 1000 randomizations (p-values from 1e10^−09^ to 8e10^−29^) (Suppl. Fig. S6). We propose this predicted set of 38 lincRNAs with conserved microsynteny across vertebrate genomes as an important dataset for experimental validation of lincRNA function in vertebrates.

## Discussion

In the current study, we have generated, assembled and annotated the first developmental transcriptome of *Tetraodon nigroviridis* despite the difficulties in breeding of this model species. This permitted us to improve the current gene-set and to explore the molecular basis of developmental processes during tetraodon embryogenesis. The transcriptome has been produced exploiting the power of high-throughput sequencing technologies and therefore is characterized by high coverage and depth which permitted us to discover, for the first time in this species, the transcription of lncRNAs. We performed extensive computational analyses to assemble and annotate the generated transcriptome as well as to analyse the temporal variations in transcripts abundances in relation to their functions. Our sequencing of polyadenylated RNA across three developmental stages resulted in the assembly of 59,913 coding transcripts from 23,390 loci and 1120 lncRNAs from 918 loci. LncRNAs detection and functional characterization is often made difficult by their low level of sequence conservation across large evolutionary distances^25^,^11^,^38^,^39^,^40^ and the tendency of current computational pipelines to classify transcripts coding for short peptides as lncRNAs^41^,^42^,^43^. We therefore exploited a stringent annotation^21^, which considers both homology and sequence composition features to classify lncRNAs.

Previous reports have shown that lncRNAs are shorter in length and have lower expression levels coupled with a smaller number of splice sites with respect to coding genes^8^,^44^. They are indeed subjected to inefficient splicing often leading to the production of mono- or bi-exonic transcripts^45^. These features have also been reported in the GENCODE catalogue of lncRNAs^6^. Accordingly, we have found lower level of splicing among the detected lncRNAs with significantly shorter transcript lengths compared to coding genes, suggesting similar transcript anatomy with other vertebrates. Tetraodon lncRNA transcripts show a drastic reduction in their intron size while their respective exon lengths remain more comparable among vertebrate species suggesting the existence of a selective constraint on lncRNA exon lengths. About 28 tetraodon lincRNA loci result to be syntenic within vertebrates, while 7 lincRNA loci show sequence conservation between teleosts including the well-studied *gas5* gene. A unique case of sequence similarity among teleosts with retained microsynteny and orientation in vertebrates has drawn our attention to the *lnc_setd1b* gene. We propose this and similar candidate lncRNAs, showing conservation of local gene order over large evolutionary distances, as prime targets for experimental validation.

More than 80% of human lincRNAs contain TEs, in sharp contrast to protein-coding genes and with an enrichment of LTRs from HERV elements at TSSs^26^. In addition, specific TEs families have been found enriched in lncRNA exons across different vertebrate species^27^. We found that, compared to other vertebrates, a sensibly lower number of tetraodon lncRNAs is associated with TEs suggesting that this association might not be a predominant feature of lncRNA evolution in tetraodon. Nonetheless, similarly to other species, the 27 tetraodon lncRNA loci, containing TE fragments embedded in their exons, remain enriched for TE sequence content with respect to coding genes. Among them, 10 loci containing LTR elements display a higher fraction of TEs sequence also with respect to the genomic average with a preference for the presence of the LTR at their transcriptional start site. This supports the potential role of TEs in the evolution and transcriptional regulation of at least a subset of tetraodon lncRNAs.

Based on their expression across developmental stages, we were able to classify both coding and lncRNAs as either maternal or embryonic specific during early development. The annotated functions of maternal and embryonic coding transcripts are in agreement with their expected functional roles during development and show similar expression dynamic with their zebrafish orthologs. We also observed that embryonic specific lincRNAs lie preferentially near genes reported to play fundamental roles in body patterning, cellular proliferation and signalling thus supporting previous reports about functional involvement of lincRNAs during early development. Therefore, even though our study presents a lack of replicates, evidences from our functional analyses, coupled with the observed expression dynamics, support the integrity of the assembly, mapping and quantification of the transcriptome herein presented.

The raw RNAseq data of the study are available in the ArrayExpress database (www.ebi.ac.uk/arrayexpress) under accession number E-MTAB-3804 and a fasta file containing the assembled transcripts is available in Suppl. Data S9.

## Methods

### RNA extraction and sequencing

All experiments with Tetraodon were performed in accordance with guidelines and regulations covered and approved by the Home Office Project Licence 44518. Breeding of fishes, extraction of eggs and collection of embryos was performed as described in a recent report^20^. Total RNA was extracted with Trizol (Invitrogen) according to the manufacturer’s protocol from eggs, whole embryo at 30% epiboly (30 epi) and whole embryo at 24 hours post fertilisation (24 hpf). The RNA samples were treated with 2U Dnase I (Qiagen) per μg RNA sample at 37 °C for 10 minutes. Digested samples were then treated with 20 mg/mL proteinase K (Sigma Aldrich) at 37 °C for 45 minutes. The quality and quantity of total RNA were assessed with the Bioanalyzer 2100 (Agilent) and no sign of degradation was detected (RIN > 9.0). Sequencing libraries were generated from total RNA samples following the Truseq RNA protocol (Illumina). Single end reads (1 × 50 nucleotides) were obtained from 3 lanes on a Hiseq1000 using SBS v3 kits (Illumina). Cluster detection and base calling were performed using RTAv1.13 (Illumina). Quality of reads was assessed with CASAVA v1.9. Sequencing reads with a mean Phred score higher than 37 were further considered for mapping and assembly.

### Quality filtering, mapping and assembly of sequenced reads

The raw sequencing reads from eggs, 30 epi and 24 hpf were processed with the Trimmomatic program^46^ to: (a) trim low quality bases, (b) filter reads with low quality and (c) filter reads smaller than 36 bases after trimming (parameters: ILLUMINACLIP::2:30:10 LEADING:3 TRAILING:3 SLIDINGWINDOW:4:15 MINLEN:36 HEADCROP:5). The raw reads were mapped on the tetraodon genome (tetNig2) using the Tophat2 software^47^ (parameters: –GTF –library-type fr-unstranded –segment-length 21 segment-mismatches 1 –raw-juncs –prefilter-multihits). A reference gene model file in the Gene Transfer Format (GTF) was used while mapping the reads. The reference GTF file comprises: pooled genomic features from Ensembl genes^48^, transmap mRNA and transmap refgene tracks of the UCSC genome browser for tetraodon^49^. The output files from Tophat2 in BAM format were converted to BigWig format using the genomeCoverageBed binary from the BEDTools package (v2.17)^50^ and the bedGraphToBigWig utility from the UCSC database (http://hgdownload.cse.ucsc.edu/admin/exe/). The Cufflinks program^51^ was used to assemble the reads mapped by using the described mapping strategy (parameters: –frag-bias-correct –library-type fr-unstranded –upper-quartile-norm –no-effective-length-correction). The transcript models generated by Cufflinks for the egg, 30 epi and 24 hpf mappings were merged together by the Cuffcompare utility from the Cufflinks software package (parameters: -V -R -r -s -C). All assembled transcripts longer than 200 bases were retained and considered in further analyses. CAGE tag data mapping transcription start sites during development in tetraodon was obtained from a previous report^23^. Custom scripts in Perl and R along with intersectBed utility from the BEDTools package were used to associate assembled transcripts to CAGE tags. A transcript is associated with a CAGE tag if the tag lies within −1000/+400 of its start coordinate (0.5 tpm cut-off).

### Annotation of assembled transcripts

Reference file of gene models in GTF format was obtained from Ensembl (ftp://ftp.Ensembl.org/pub/release-72/gtf/tetraodon_nigroviridis/). The reference GTF file from Ensembl was converted in refFlat format (http://genome.ucsc.edu/goldenPath/gbdDescriptionsOld.html#RefFlat) using the UCSC utility gtfToGenePred (http://hgdownload.cse.ucsc.edu/admin/exe/) and a custom script in the Perl language. The reference file in refFlat format was compared with the mapped reads from eggs, 30 epi and 24 hpf to extract the percentage of reads mapping to different genomic features using the CollectRnaSeqMetrics.jar utility from the Picard tools software package v1.88 (http://picard.sourceforge.net/). The annotation of the assembled transcripts was performed with Annocript pipeline v1.1^21^. According to the Annocript results, potential long non-coding RNAs were identified as transcripts not showing any conservation against known proteins, domains, small and ribosomal RNAs and an ORF shorter than 100 aa. In order to classify *bona-fide* lncRNAs we calculated the distribution of the non-coding potential (NCP) scores associated to the potential lncRNAs from Annocript and defined as cutoff the mean value of their NCP score. A score greater than the mean NCP score of all potential lncRNAs (0.76) was used to call the final lncRNA set while the remaining transcripts were placed within coding class. It is worth noticing that Annocript uses Portrait^22^ to generate the NCP score. According to Portrait authors, a non-coding potential score bigger than 0.5 is sufficient to classify a transcript as non-coding. We applied more stringent conditions and despite the combination of multiple parameters and the application of these stringent cutoffs we were still able to identify more than 3800 transcripts likely representing lncRNAs. Mono-exonic lncRNAs irrespective of transcriptional orientation were filtered if they overlapped a coding gene or lay 5KB downstream of a coding gene. The rest lncRNA transcripts lying 5 Kb downstream of coding genes or overlapping coding genes and transcribed in the same strand as the coding gene were filtered. This step was taken to avoid classifying potential alternative polyadenylated transcripts of coding genes as lncRNAs^52^. The filtering resulted in a final set of 1120 lncRNA transcripts. The coordinates of the predicted lncRNAs with the predicted coding transcripts and coding genes from Ensembl (v72) were checked with the intersectBed program from the BEDTools package. All lncRNA transcripts not overlapping a coding locus were classified as long intergenic non-coding RNAs (lincRNAs). The assembled transcripts were merged and mapped to the reference Ensembl GTF file (v72) and the tetraodon GenBank ESTs from the UCSC genome browser using Cuffcompare (parameters: -V -R -r -s -C). Assembled loci containing coding transcripts neither mapped to an Ensembl gene nor to a GenBank tetraodon EST were classified as novel coding loci. The transcript sequences of the novel coding loci were compared against Ensembl cDNA (ftp://ftp.ensembl.org/pub/release-72/) and Unigene sequences (ftp://ftp.ncbi.nlm.nih.gov/repository/UniGene/) of seven vertebrate species (human, mouse, *Xenopus tropicalis*, medaka, stickleback, fugu) using tblastx program (-word_size 4 -threshold 18 -evalue 1e-3). On the basis of the results from this analysis the novel loci were classified as vertebrate conserved, teleost conserved or tetraodon specific.

### Differential expression analysis of the assembled transcripts

The raw read counts for all exons of assembled transcripts were obtained with the multiBamCov utility from BEDTools software package (parameter: -split). The sum of read counts for all exons of a given transcripts was considered as the raw count of the transcript. The bioconductor edgeR 3.8.6 package^53^ was used to calculate the differential expression of transcripts across the developmental stages. This package measures the significance of the variation in expression levels using the dispersion of the expression levels among sample replicates. In the absence of replicates the software can infer the dispersion value using the fluctuations in the expression levels of selected *housekeeping* genes among the different samples. Given that the analysed dataset was only composed by single samples for each stage (i.e. no replicates) and there is no information about *housekeeping* genes in tetraodon, we used the following strategy to infer an acceptable dispersion value. All the transcripts showing less than 1 per million mapped reads in the sum of the experiments were discarded to filter lowly expressed or background-biased transcripts. For all the remaining transcripts we calculated the standard deviation among the expression levels. Transcripts were then sorted in descending order based on the standard deviation values. Next, we filtered out all the genes that did not get any match against the SwissProt database in the annotation step. Then, based on the standard deviations and the annotations, we selected the transcripts showing the lowest variations until we were able to collect 100 different genes (based on the annotations). The selected 100 genes, corresponding to 305 transcripts, were considered bona-fide *housekeeping*, and the dispersion value was calculated using them. The calculated dispersion value (0.35) was used for the differential expression analysis in edgeR. Transcripts with more than 0.5 reads per million mapped reads in at least one sample were retained for the analysis. The following comparisons were executed using the exactTest function with default parameters: eggs vs 30 epiboly, eggs vs 24 hours post fertilisation, 30 epiboly vs 24 hours post fertilisation. According to the comparison, significantly up/downregulated transcripts were selected, based on a stringent FDR and logarithmic fold change cut-off (FDR < = 0.01; absolute log fold change > = 2). The maternal specific list of transcripts was prepared selecting only those transcripts resulting significantly upregulated in the eggs in both the comparisons involving the eggs samples. The embryonic list of transcripts was prepared selecting only those transcripts resulting significantly downregulated in the eggs in both comparisons involving the eggs samples. Further, coding transcripts flanking up to a distance of 10 KB from all lincRNAs and embryonic lincRNAs were identified using the windowBed utility from the BedTools package (parameter: -w 10000). To reduce the probability of a GO term to be associated with a lncRNA due to an overlapping coding gene we considered only intergenic lncRNAs (lincRNAs) in this analysis. The Gene Ontology^54^ enrichment analysis was performed on the GO mapping done in the annotation step using a custom R script exploiting the Fisher exact test and p-value FDR correction to select significantly enriched GO classes in the group of differentially expressed transcripts compared to the total transcriptome (minimum representatives for a GO class: 5 transcripts; FDR < = 0.05).

### Comparison of expression abundance between maternal and zygotic transcripts in zebrafish with their tetraodon orthologs

The expression of zebrafish genes during early development and the lists of genes reported to be maternal, zygotic and maternal/zygotic were obtained from previously published studies^14^,^15^. To make our data comparable with this report, FPKM expression values of the assembled transcripts in tetraodon were calculated by Cuffdiff program from the Cufflinks software package (parameter: –frag-bias-correct –multi-read-correct –no-effective-length-correction –upper-quartile-norm –max-frag-multihits 20). The mean FPKM of all transcripts mapped to an Ensembl gene was considered to be the level of expression for that gene. Orthologous protein coding genes mapping between zebrafish and tetraodon was obtained using the Bioconductor^55^ biomaRt^56^ package.

### Detection of sequence conservation

The lncRNA dataset for human was downloaded from Gencode v17^57^. All lncRNAs annotated in the Ensembl database version 72 were considered for mouse^48^ and lncRNAs predicted by two prior published studies^8^,^39^ along with those classified by Ensembl database version 72 were pooled together for zebrafish. MultiZ^58^ alignments of eight vertebrate genomes with the zebrafish as reference (other species: human, mouse, medaka, stickleback, fugu, tetraodon, *Xenopus tropicalis*) were downloaded in the Multiple Alignment Format (MAF) from the UCSC database (http://hgdownload.soe.ucsc.edu/goldenPath/danRer7/multiz8way/multiz8way.maf.gz). Genome wide alignment blocks for all the species were extracted in BED format from the MultiZ alignment using a custom Perl script. The intersectBed utility from BEDTools package was used to find the intersection of the exonic coordinates of the assembled transcripts with the aligned blocks in tetraodon. Each aligned block is assigned a similarity score. The product of this score with the fraction of the aligned block overlapping an exon was taken as the conservation score for the exon. The sum of scores for all exons of a transcript was taken as the conservation score for the transcript. The MultiZ aligned coordinates for human, mouse, zebrafish and tetraodon were parsed into genomic coordinates using custom Perl scripts and compared against the respective lincRNA exons using the intersectBED utility. Further the lincRNA transcripts in all species were shuffled genome-wide using the shuffleBED utility (parameter: -excl) excluding the shuffled regions falling within a coding or lncRNA locus and compared with the respective alignment coordinates. Specifically, the multiz8way alignments were parsed to extract only genomic regions which align concurrently between human, mouse, zebrafish and tetraodon. These alignments were considered as vertebrate conserved genomic blocks. Each block is characterized by four elements containing the genomic coordinate of each candidate species. If each element within a given block overlaps a lncRNA exon (in each respective species), the overlapping lncRNAs are predicted to be conserved. Next, the multiz8way alignments were parsed to obtain genomic regions aligned between zebrafish and tetraodon that were considered as teleost conserved genomic blocks. In this case each block has two elements containing the genomic coordinate of zebrafish and tetraodon. Hence if both elements within a block overlap lncRNA exons (in each respective species), the overlapping lncRNAs are predicted to be conserved. Finally only intergenic lncRNAs (lincRNAs) were considered for generating the final set of conserved lncRNAs. An exception was made for two lncRNA loci (lncRNA *gas5* and lncRNA *setd1b*) which were included in the conservation results after manual curation, since they are not predicted as lincRNAs on account of an overlapping transcript without homology to any coding gene but with a slightly lower non-coding potential than the threshold cut-off. However overwhelming evidence from CAGE, RNAseq, conservation of microsynteny and published literature led us to establish these transcripts as putative lincRNAs.

### Identification of microsynteny

The genomic coordinates of coding genes and their homology relationships for each organism were downloaded from the Ensembl Compara database^59^ (version 72). The data retrieval from the Ensembl database was carried out using the Bioconductor package biomaRt (Durinck *et al*.^56^). An in-house lincRNA microsynteny detection pipeline (SynLinc) was developed to predict putative microsyntenic lincRNAs between zebrafish/tetraodon, human/zebrafish, human/mouse and mouse/zebrafish considering only immediate flanking coding genes for each lincRNA. The pipeline can compare the lncRNA dataset of a pair of organisms at a time. Only intergenic long non-coding RNAs were considered for the analysis. Pairs of intergenic lncRNAs from two organisms whose immediate flanking coding genes share homology are classified as microsyntenic. All possible pairwise comparisons were performed between human, mouse, zebrafish, tetraodon using the pipeline. Finally the lncRNAs which share homologous coding genes in all the organsisms were classified as vertebrate microsyntenic. To perform the randomization analysis in Supp. Fig. 6 we classified intergenic regions containing a lincRNA in human (Gencode v17), mouse (Ensembl v72), zebrafish (Ensembl v72 + Pauli *et al*. + Ulitsky *et al*.) and tetraodon as lincIGs and calculated the percentage of lincIGs in each organism which retain microsynteny of closest coding gene (upstream or downstream) in all the organisms. We then shuffled the lincRNAs coordinates in each organism to get a new set of random lincRNAs. The intergenic regions containing a randomized lncRNA were classified as randIGs and we calculated the percentage of randIGs which retain microsynteny in the four species. Randomizations were repeated 1000 times producing a percentage distribution of randIGs resulting miscrosyntenic in human, mouse, zebrafish and tetraodon. Z-scores were used to calculate the significances.

### Analyses of repeats

Repeat elements predicted by the RepeatMasker program for human, mouse, zebrafish, fugu and tetraodon were downloaded from the Ensembl database (v72) in BED format using the Ensembl Perl API^60^. The Ensembl RepeatMasker predictions for tetraodon are not classified into families by default. Hence the tetraodon repeat classes were manually associated to family according to their classification in human, mouse, zebrafish and fugu. The exon coordinates of lincRNAs and coding transcripts for each species were merged in a non-redundant set using the mergeBED utility. For each species the repeat elements were compared against coding and long non-coding exons using the intersectBED utility. Repeat elements overlapping the start coordinate (end coordinate in case of minus strand transcripts) of a transcript were classified as start site associated.

## Additional Information

**How to cite this article**: Basu, S. *et al*. The *Tetraodon nigroviridis* reference transcriptome: developmental transition, length retention and microsynteny of long non-coding RNAs in a compact vertebrate genome. *Sci. Rep.* **6**, 33210; doi: 10.1038/srep33210 (2016).

## Supplementary Material

Supplementary Information

Supplementary Table S2

Supplementary Table S5

Supplementary Table S6

Supplementary Data S1

Supplementary Data S2

Supplementary Data S3

Supplementary Data S4

Supplementary Data S5

Supplementary Data S7

Supplementary Data S9

## Figures and Tables

**Table 1:** Number of transcripts and their corresponding loci showing differential expression.

	Coding transcripts		LncRNAs		LincRNAs	
	Transcript	Loci	Transcript	Loci	Transcript	Loci
Differential in any stage	10,965	4947	192	149	115	87
Maternal	307	137	10	8	7	5
Embryonic	4221	1971	81	62	50	38
