# Supplementary material for: The Tetraodon nigroviridis reference transcriptome: developmental transition, length retention and microsynteny of long non-coding RNAs in a compact vertebrate genome
Source: Sci Rep. 2016 Sep 15;6:33210. doi: 10.1038/srep33210 (PMC5024134; doi:10.1038/srep33210)
Supplement: Supplementary Information [file srep33210-s1.pdf]

Supplementary Figures and Legends

The *Tetraodon nigroviridis* reference transcriptome: developmental transition, length retention and microsynteny of long non-coding RNAs in a compact vertebrate genome

Swaraj Basu[1,5], Yavor Hadzhiev[2], Giuseppe Petrosino[1], Chirag Nepal[4], Jochen Gehrig[3], Olivier Armant[3], Marco Ferg[3], Uwe Strahle[3], Remo Sanges[1*] and Ferenc Müller[2*]

1. Biology and Evolution of Marine Organisms, Stazione Zoologica Anton Dohrn, Villa Comunale, Naples - 80121, Italy.

2. Institute of Cancer and Genomic Sciences, College of Medical and Dental Sciences, University of Birmingham, Edgbaston, Birmingham, B15 2TT, UK.

3. Karlsruhe Institute of Technology (KIT), Campus North, Institute of Toxicology and Genetics (ITG), Hermann-von-Helmholtz-Platz 1, 76344 Eggenstein-Leopoldshafen, Germany.

4. BRIC - Biotech Research & Innovation Centre, University of Copenhagen, Ole Maaløes Vej 5, DK-2200 Copenhagen N, Denmark.

5. Present address: Department of Medical Biochemistry and Cell Biology, Institute of Biomedicine, Sahlgrenska Academy, University of Gothenburg, 405 30 Gothenburg, Sweden.

* Correspondence to be addressed to: f.mueller@bham.ac.uk and remo.sanges@gmail.com

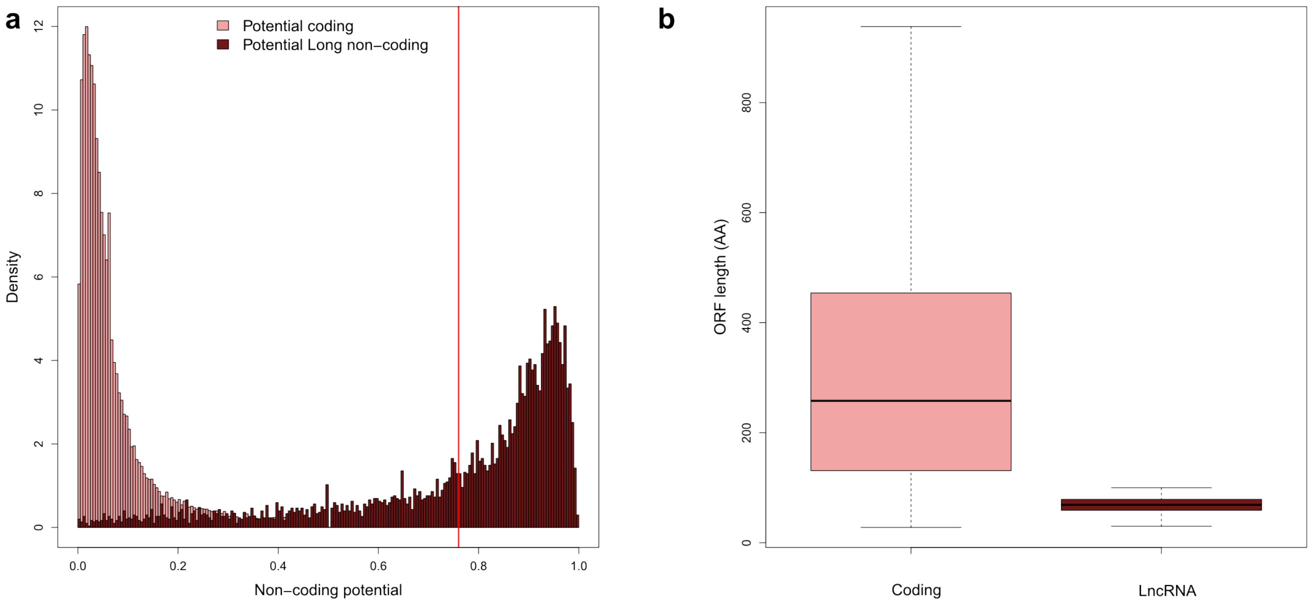

Supplementary figure S1. **Distribution of non coding potential scores and ORF length for the assembled transcripts**. **a)** Distribution of non-coding potential score for coding transcripts and potential long non-coding transcripts (without annotation and ORF <= 100 AA). The y axis represents the frequency, the bars in pink represent coding transcripts while the green bars represent the potential lncRNAs (transcripts without any annotation and an ORF < 100 AA). The red line marks the chosen cut-off non-coding potential score (0.76) for a potential lncRNA to be placed in the lncRNA dataset. **b)** Distribution of ORF length for coding and lncRNA transcripts (final lncRNA set post filtering with a non-coding potential score >0.76).

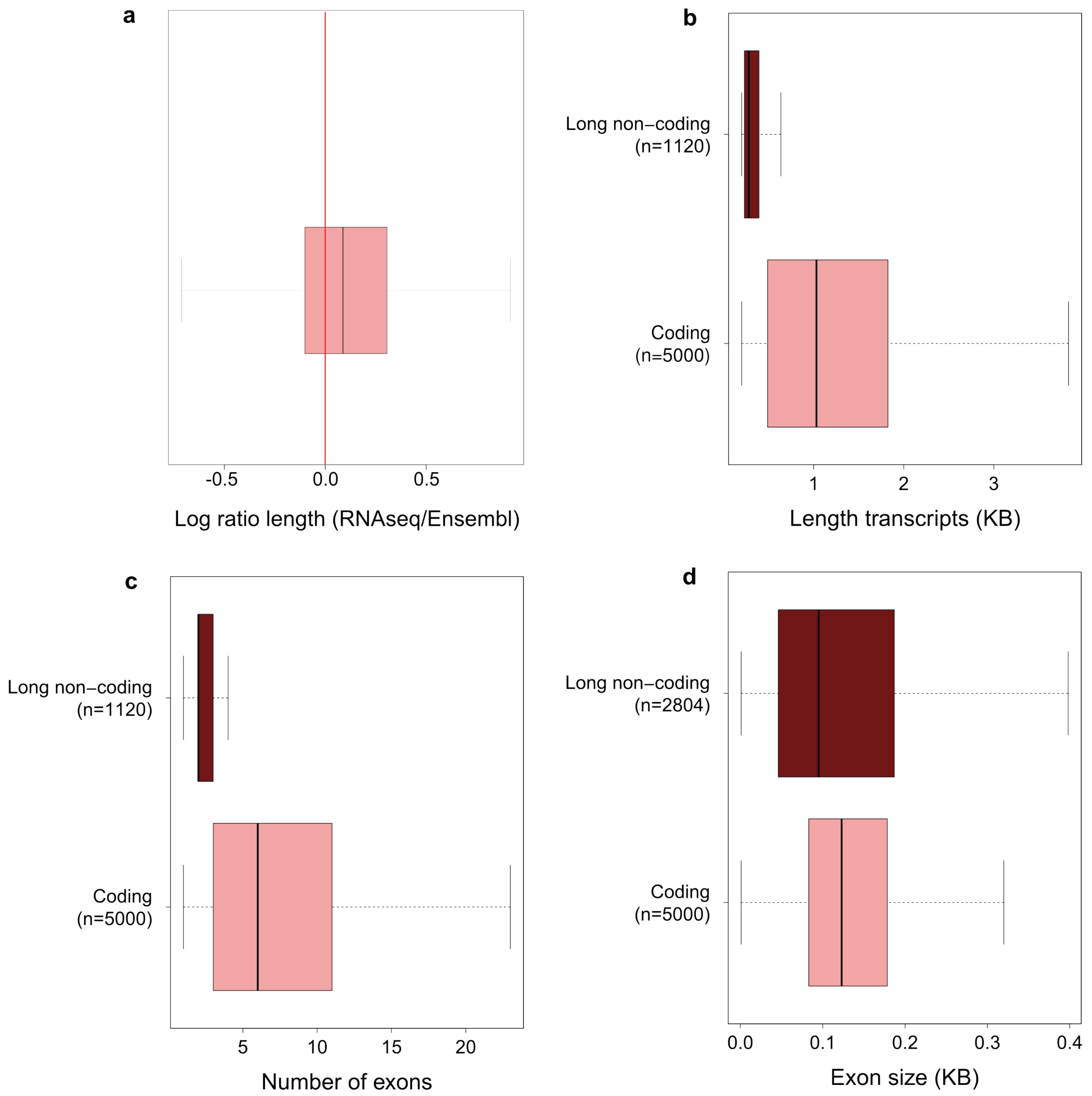

Supplementary Figure S2. Structural features of coding and long non-coding transcripts suggest similarity of the non-coding transcripts with those detected in other vertebrates. The average length of assembled transcripts is higher compared to the Ensembl gene models indicating a better coverage of the UTR regions by the RNAseq experiment. **a)** Length ratio of longest transcript for each mapped coding loci and its corresponding Ensembl coding gene **b)** Length of transcripts. **c)** Number of exons. **d)** Size of exons.

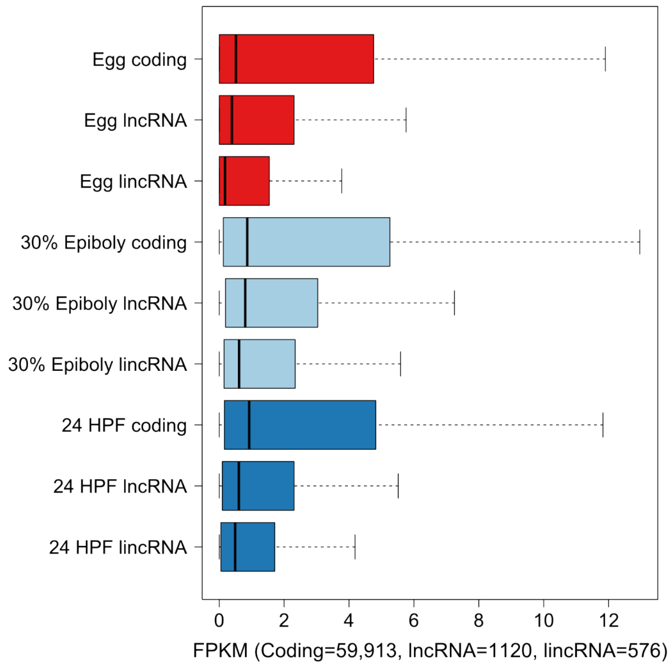

Supplementary figure S3. Distribution of expression for coding, long non-coding and long intergenic non-coding transcripts across early developmental stages in tetraodon (FPKM).

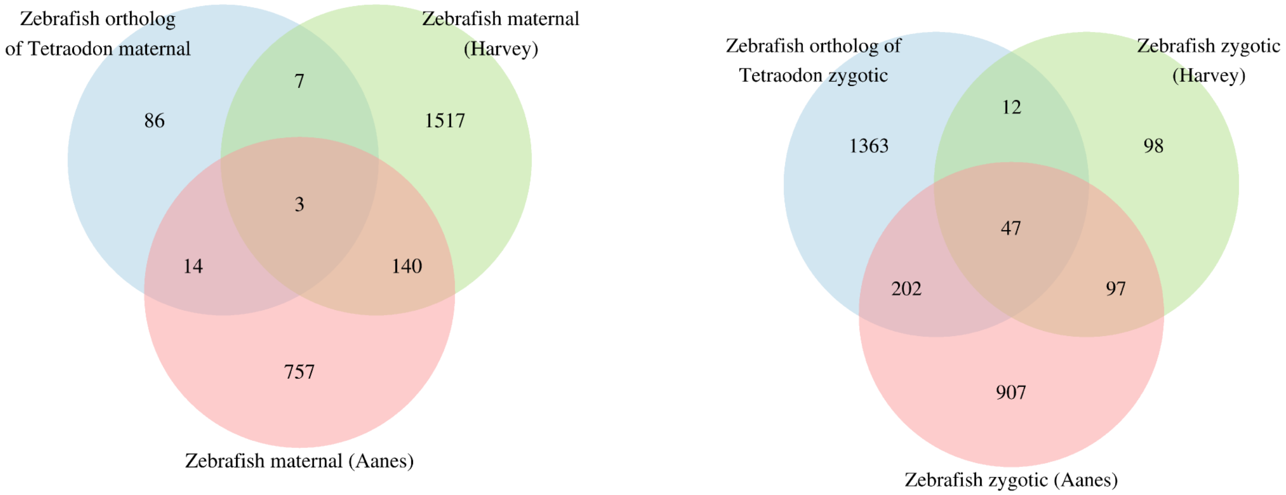

Supplementary figure S4. Comparison of genes predicted to be maternal and embryonic specific in zebrafish and tetraodon. a) Maternal genes. **b)** Embryonic genes.

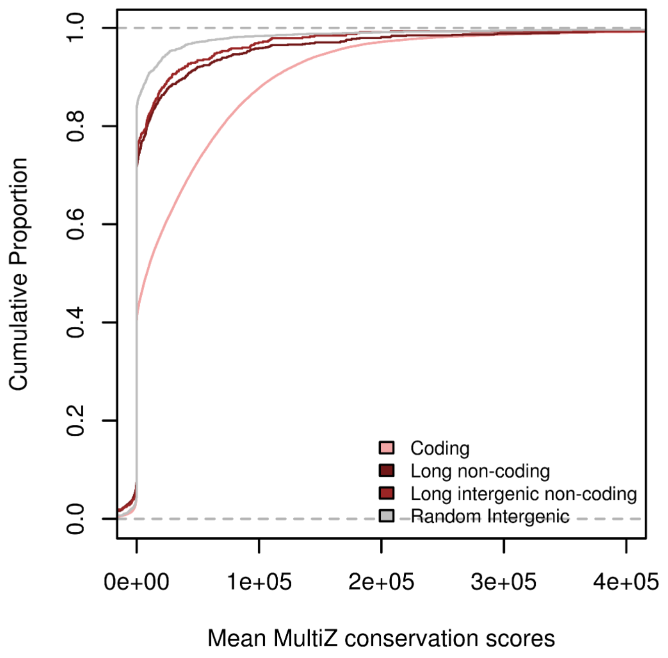

Supplementary figure S5. Mean MultiZ 8 way whole genome alignment scores of tetraodon for coding, long non-coding and long intergenic non-coding transcripts. The alignment scores are calculated by aligning the zebrafish genome with the human, mouse, *X. tropicalis*, tetraodon, medaka, fugu and stickleback genomes. The x-axis represents the mean MultiZ8way alignment scores and the y-axis represents the cumulative proportions of the transcripts.

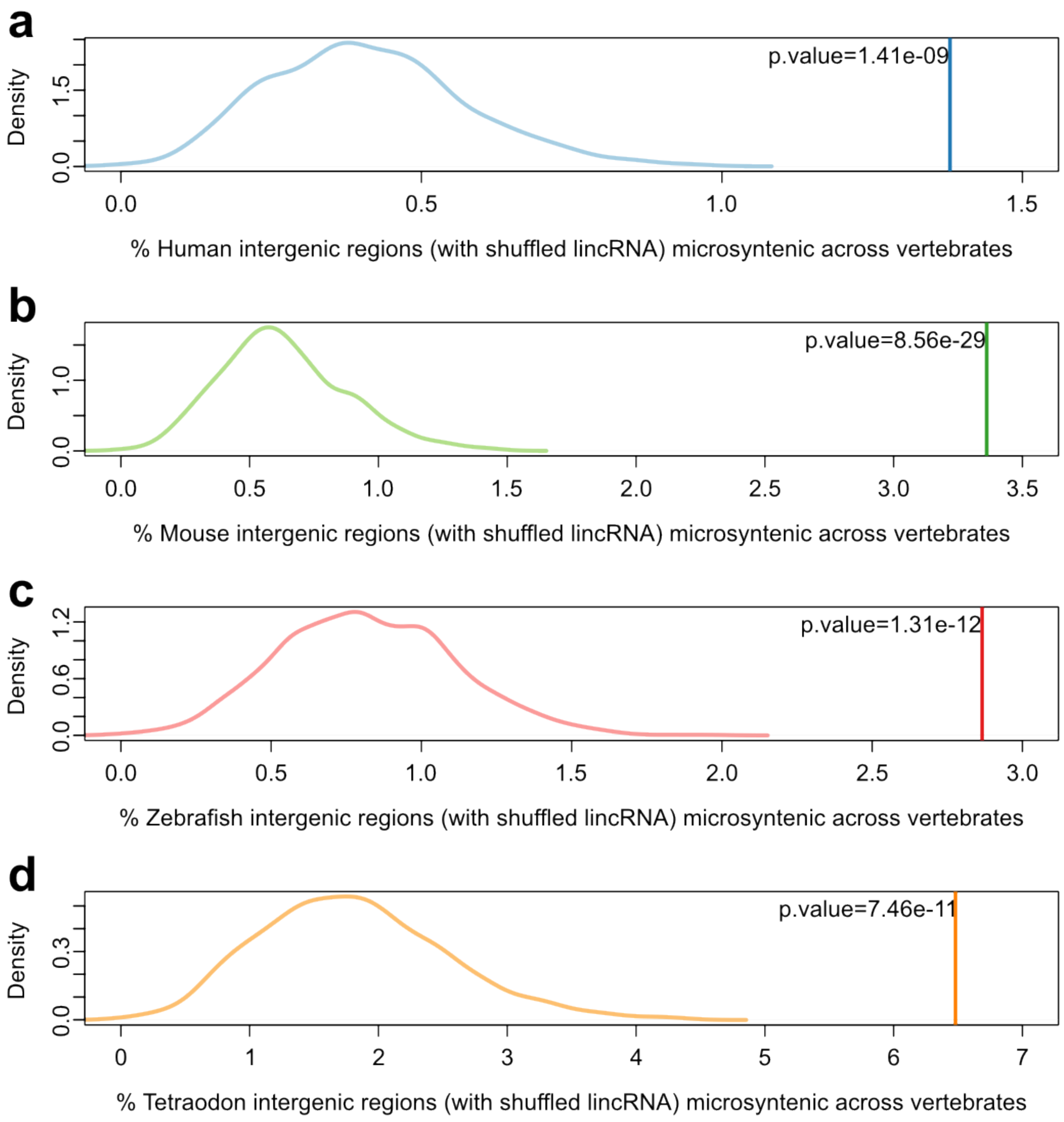

Supplementary figure S6. Percentage of intergenic regions containing randomized lincRNAs retaining microsynteny in vertebrates based on orthology of a proximal coding gene. a) human **b)** mouse **c)** zebrafish **d)** tetraodon. The vertical line in each panel shows the true percentage of intergenic regions containing a mycrosintenic lincRNA.

Supplementary table S1: Table containing statistics for filtering and mapping of raw sequencing reads (Page 10 of this PDF).

Supplementary table S2: Tables containing tetraodon maternal and embryonic specific genes and their zebrafish orthologs along with expression of the tetraodon gene (from early development samples) and expression of the zebrafish gene as generated by two previous studies (Aanes *et al*, 2011; Harvey *et al*, 2013) .

Supplementary table S3: Tables containing GO enrichment analysis on subset of tetraodon transcripts predicted to be novel, maternal specific, embryonic specific and coding transcripts associated with embryonic specific lincRNAs (Page 11 of this PDF).

Supplementary table S4: Tables containing statistics and genomic coordinates of lincRNAs predicted to show sequence conservation in teleost fishes (Page 19 of this PDF).

Supplementary table S5: Tables about lincRNAs predicted to show microsynteny on comparison of human, mouse, zebrafish and tetraodon lincRNAs.

Supplementary table S6: Tables containing species specific information for vertebrate microsyntenic lincRNAs.

Supplementary data S1: GTF file containing the genomic coordinates on Ensembl v72 for the assembled tetraodon early development transcriptome.

Supplementary data S2: Tab delimited text file containing the mapping between the assembled transcriptome and the Ensembl gene models based on Ensembl.

Supplementary data S3: Tab delimited text file containing the results for the annotations of all the assembled transcripts as generated by Annocript.

Supplementary data S4: The assembled coding transcripts in BED format.

Supplementary data S5: The assembled lncRNAs in BED format.

Supplementary data S6: List of IDs of all the assembled non-coding transcripts grouped into classes based on their genomic location (Page 21 of this PDF).

Supplementary data S7: Tab delimited text file containing, for each transcript, the number of reads mapping from every sequenced sample.

Supplementary data S8: List of IDs of all the transcripts predicted to be maternal or embryonic specific during early development in tetraodon (Page 30 of this PDF).

Supplementary data S9: FASTA format file containing the sequences of all the assembled transcripts.

Supplementary table S1: Table containing statistics for filtering and mapping of raw sequencing reads.

Sample	Total number of reads	Quality filtered	Mapped reads	% Mapped
Egg	229741278	215219078	148750648	69.1159210337
30% Epiboly	248151367	226292377	151424127	66.9152576006
24 hpf	239853692	224089479	166611571	74.3504656013

Supplementary table S3: Tables containing GO enrichment analysis on subset of tetraodon transcripts predicted to be novel, maternal specific, embryonic specific and coding transcripts associated with embryonic specific lincRNAs.

Novel transcripts

ID	Number of novel coding transcripts	Total novel coding transcripts	Number of coding transcripts	Total coding transcripts	pval	definition	padj
GO:0031047	9	6066	30	59913	0.0032215264	gene silencing by RNA	0.0354367907
GO:0035188	5	6066	8	59913	0.0007554944	hatching	0.009065933
GO:0009059	5	6066	7	59913	0.0003444641	macromolecule biosynthetic process	0.0050305634
GO:0032793	9	6066	23	59913	0.0003353709	positive regulation of CREB transcription factor activity	0.0050305634
GO:0048785	5	6066	7	59913	0.0003444641	hatching gland development	0.0050305634
GO:0030001	7	6066	12	59913	8.02E-005	metal ion transport	0.001282462
GO:0043087	6	6066	8	59913	4.87E-005	regulation of GTPase activity	0.0008271237
GO:0042989	5	6066	5	59913	4.44E-005	sequestering of actin monomers	0.0007999707
GO:0009607	10	6066	15	59913	3.09E-007	response to biotic stimulus	5.86E-006
GO:0015074	18	6066	35	59913	9.54E-010	DNA integration	1.91E-008
GO:0006278	24	6066	52	59913	2.70E-011	RNA-dependent DNA replication	5.68E-010

Maternal specific transcripts

ID	Number of maternal specific coding transcripts	Total maternal specific coding transcripts	Number of coding transcripts	Total coding transcripts	pval	definition	padj
GO:0006351	10	307	731	59913	0.0014885902	transcription, DNA-dependent	0.0062940096
GO:0007264	9	307	612	59913	0.0012588019	small GTPase mediated signal transduction	0.0062940096
GO:0006184	5	307	183	59913	0.0001403324	GTP catabolic process	0.0008419943
GO:0007018	7	307	320	59913	8.39E-005	microtubule-based movement	0.0005876317
GO:0051056	6	307	160	59913	1.90E-007	regulation of small GTPase mediated signal transduction	1.52E-006
GO:0030036	7	307	155	59913	1.83E-010	actin cytoskeleton organization	1.64E-009
GO:0042981	6	307	85	59913	5.93E-014	regulation of apoptotic process	5.93E-013
GO:0007067	6	307	45	59913	3.27E-025	mitosis	3.60E-024
GO:0060828	8	307	16	59913	1.38E-099	regulation of canonical Wnt receptor signaling pathway	1.66E-098
GO:2000053	8	307	8	59913	7.71E-150	regulation of Wnt receptor signaling pathway involved in dorsal/ventral axis specification	1.00E-148
GO:0048263	12	307	25	59913	1.10E-150	determination of dorsal identity	1.55E-149

Embryonic specific transcripts

ID	Number of embryonic specific coding transcripts	Total embryonic specific coding transcripts	Number of coding transcripts	Total coding transcripts	pval	definition	padj
GO:0035050	8	4221	30	59913	0.0005349675	embryonic heart tube development	0.0444023026
GO:0007634	5	4221	12	59913	0.000470453	optokinetic behavior	0.0395180483
GO:0034446	6	4221	17	59913	0.0004000962	substrate adhesion-dependent cell spreading	0.034008174
GO:0030903	7	4221	22	59913	0.0002916939	notochord development	0.0250856735
GO:0051297	8	4221	28	59913	0.0002807559	centrosome organization	0.0244257673
GO:0001707	5	4221	11	59913	0.0002547031	mesoderm formation	0.0231779808
GO:0007412	5	4221	11	59913	0.0002547031	axon target recognition	0.0231779808
GO:0007413	5	4221	11	59913	0.0002547031	axonal fasciculation	0.0231779808
GO:0030177	5	4221	11	59913	0.0002547031	positive regulation of Wnt receptor signaling pathway	0.0231779808
GO:0061001	6	4221	16	59913	0.0002463884	regulation of dendritic spine morphogenesis	0.0226677366
GO:0016042	8	4221	27	59913	0.0001975483	lipid catabolic process	0.0183719938
GO:0048484	7	4221	21	59913	0.0001924377	enteric nervous system development	0.0180891451
GO:0048870	8	4221	26	59913	0.0001360163	cell motility	0.0129215464
GO:0070098	9	4221	32	59913	0.0001284841	chemokine-mediated signaling pathway	0.0123344783
GO:0048854	5	4221	10	59913	0.0001269648	brain morphogenesis	0.0123155883

GO:0016055	25	4221	159	59913	0.0001125797	Wnt receptor signaling pathway	0.011032807
GO:0001841	6	4221	14	59913	8.05E-005	neural tube formation	0.0079714079
GO:0090305	16	4221	80	59913	7.78E-005	nucleic acid phosphodiester bond hydrolysis	0.007775205
GO:0001568	7	4221	19	59913	7.58E-005	blood vessel development	0.0076588542
GO:0048665	5	4221	9	59913	5.73E-005	neuron fate specification	0.0058975418
GO:0051262	5	4221	9	59913	5.73E-005	protein tetramerization	0.0058975418
GO:0009880	9	4221	29	59913	4.32E-005	embryonic pattern specification	0.0044934642
GO:0006783	6	4221	13	59913	4.21E-005	heme biosynthetic process	0.0044593012
GO:0048546	6	4221	13	59913	4.21E-005	digestive tract morphogenesis	0.0044593012
GO:0030902	8	4221	23	59913	3.82E-005	hindbrain development	0.0040839271
GO:0006259	11	4221	40	59913	2.72E-005	DNA metabolic process	0.0029675539
GO:0070121	11	4221	40	59913	2.72E-005	Kupffer's vesicle development	0.0029675539
GO:0033339	7	4221	17	59913	2.55E-005	pectoral fin development	0.0028008456
GO:0060034	5	4221	8	59913	2.28E-005	notochord cell differentiation	0.0025341983
GO:0007156	43	4221	313	59913	2.18E-005	homophilic cell adhesion	0.0024427582
GO:0030947	6	4221	12	59913	2.04E-005	regulation of vascular endothelial growth	0.0023083003

						factor receptor signaling pathway	
GO:0007368	13	4221	52	59913	1.94E-005	determination of left/right symmetry	0.002207696
GO:0006979	11	4221	38	59913	1.38E-005	response to oxidative stress	0.001582974
GO:0001706	6	4221	11	59913	9.10E-006	endoderm formation	0.0010556859
GO:0071910	5	4221	7	59913	7.81E-006	determination of liver left/right asymmetry	0.0009133639
GO:0031016	9	4221	25	59913	7.37E-006	pancreas development	0.0008702196
GO:0042074	14	4221	54	59913	5.03E-006	cell migration involved in gastrulation	0.0005984372
GO:0015074	11	4221	35	59913	4.40E-006	DNA integration	0.0005275802
GO:0035622	8	4221	18	59913	2.33E-006	intrahepatic bile duct development	0.0002820961
GO:0046168	5	4221	6	59913	2.20E-006	glycerol-3-phosphate catabolic process	0.0002678725
GO:0001570	15	4221	55	59913	9.15E-007	vasculogenesis	0.0001125926
GO:0003171	5	4221	5	59913	4.79E-007	atrioventricular valve development	6.13E-005
GO:0014707	5	4221	5	59913	4.79E-007	branchiomeric skeletal muscle development	6.13E-005
GO:0021571	5	4221	5	59913	4.79E-007	rhombomere 5 development	6.13E-005
GO:0021572	5	4221	5	59913	4.79E-007	rhombomere 6 development	6.13E-005
GO:0021703	5	4221	5	59913	4.79E-007	locus ceruleus development	6.13E-005
GO:0021535	6	4221	8	59913	4.00E-007	cell migration in hindbrain	5.16E-005

GO:0048709	8	4221	15	59913	2.39E-007	oligodendrocyte differentiation	3.11E-005
GO:0021523	7	4221	11	59913	2.17E-007	somatic motor neuron differentiation	2.84E-005
GO:0007050	18	4221	68	59913	1.29E-007	cell cycle arrest	1.70E-005
GO:0060059	11	4221	27	59913	8.26E-008	embryonic retina morphogenesis in camera-type eye	1.10E-005
GO:0001525	24	4221	106	59913	6.08E-008	angiogenesis	8.14E-006
GO:0030335	8	4221	13	59913	3.62E-008	positive regulation of cell migration	4.89E-006
GO:0018149	10	4221	21	59913	3.25E-008	peptide cross-linking	4.42E-006
GO:0045892	21	4221	82	59913	2.42E-008	negative regulation of transcription, DNA-dependent	3.32E-006
GO:0016339	6	4221	6	59913	2.08E-008	calcium-dependent cell-cell adhesion	2.87E-006
GO:0051091	8	4221	12	59913	1.22E-008	positive regulation of sequence-specific DNA binding transcription factor activity	1.71E-006
GO:2000223	8	4221	12	59913	1.22E-008	regulation of BMP signaling pathway involved in heart jogging	1.71E-006
GO:0048332	7	4221	8	59913	4.70E-009	mesoderm morphogenesis	6.63E-007
GO:0006278	17	4221	52	59913	3.14E-009	RNA-dependent DNA replication	4.46E-007
GO:0009887	13	4221	30	59913	1.35E-009	organ morphogenesis	1.93E-007
GO:0043049	10	4221	17	59913	1.02E-009	otic placode formation	1.46E-007

GO:0048339	8	4221	10	59913	9.63E-010	paraxial mesoderm development	1.40E-007
GO:0071526	7	4221	7	59913	9.08E-010	semaphorin-plexin signaling pathway	1.33E-007
GO:0045893	29	4221	117	59913	1.37E-010	positive regulation of transcription, DNA-dependent	2.02E-008
GO:0001947	25	4221	85	59913	1.54E-011	heart looping	2.28E-009
GO:0036065	14	4221	26	59913	2.08E-012	fucosylation	3.09E-010
GO:0001756	34	4221	104	59913	2.40E-017	somitogenesis	3.59E-015

Coding transcripts proximal to embryonic lincRNA

ID	Number of coding transcripts near embryonic specific lincRNAs	Total coding transcripts near embryonic specific lincRNAs	Number of coding transcripts near lincRNAs	Total coding transcripts near lincRNAs	pval	definition	padj
GO:0035023	7	215	23	2566	0.0020371027	regulation of Rho protein signal transduction	0.0061113081
GO:0001947	5	215	10	2566	0.0006038804	heart looping	0.0024155216
GO:0007169	5	215	9	2566	0.0003044458	transmembrane receptor protein tyrosine kinase signaling pathway	0.0015222289
GO:0031018	5	215	8	2566	0.0001379934	endocrine pancreas development	0.0008279603
GO:0006200	5	215	7	2566	5.48E-005	ATP catabolic process	0.0003833798
GO:0006633	6	215	8	2566	4.70E-006	fatty acid biosynthetic process	4.23E-005
GO:0007018	5	215	5	2566	4.95E-006	microtubule-based movement	4.23E-005
GO:0006486	9	215	19	2566	3.33E-006	protein glycosylation	3.33E-005
GO:0009887	7	215	7	2566	2.76E-008	organ morphogenesis	3.31E-007
GO:0048332	7	215	7	2566	2.76E-008	mesoderm morphogenesis	3.31E-007
GO:0036065	9	215	9	2566	1.57E-010	fucosylation	2.04E-009

Supplementary table S4: Tables containing statistics and genomic coordinates of lincRNAs predicted to show sequence conservation in teleost fishes.

Statistics of lincRNA showing sequence consrevation in comparison to shuffled genomic features in zebrafish and tetraodon

	**Total transcripts (zebrafish)**	**MultiZ conserved transcripts (zebrafish)**	**Total transcripts (tetraodon)**	**MultiZ conserved transcripts (tetraodon)**
lincRNAs	2699	27	576	10
Shuffled elements lincRNA size and structure matched	13140	71	14970	60

List of lincRNA which show sequence conservation at the exon level between zebrafish and tetraodon

	**LincRNA loci (including proximal coding genes)**				**Feature overlapping conserved region**				**Number of lncRNA loci between flanking coding genes**				**Synteny of flanking coding genes**	
**Name**	**Tetraodon**	**Zebrafish**	**Mouse**	**Human**	**Tetraodon**	**Zebrafish**	**Mouse**	**Human**	**Tetraodon**	**Zebrafish**	**Mouse**	**Human**	**Teleost**	**Mammals**
linc_gart	chr3:2,595,172-2,677,286	chr1:1,011,559-1,068,134	Null	Null	lincRNA	lincRNA	Null	Null	1	1	Null	Null	Yes	Null
linc_oxr1	chr8:1,085,318-1,151,490	chr16:46,337,355-46,509,235	Null	Null	lincRNA	lincRNA	Coding exon	Coding exon	1	1	Null	Null	Yes	Yes
linc_narg2	chr13:7,566,2	chr25:35,0	Null	Null	lincRNA	lincRNA;	Coding	Coding	1	1	Null	Null	Yes	Yes

	84-7,577,232	06,379-35,025,324				coding UTR in v74	UTR	UTR					
linc_gas5	chr1:15,659,150-15,667,229	chr8:17,123,506-17,137,329	chr1:162,961,363-162,972,603	chr1:173,826,597-173,840,966	lincRNA*; snoRNA	lincRNA*; snoRNA	snoRNA	snoRNA	1	1	1	1	Yes	Null
linc_pag2g4	chr11:7,317,977-7,323,647	chr23:25,587,078-25,598,612	Null	Null	lincRNA*	lincRNA*	Null	Null	1	1	Null	Null	Yes	Null
linc_vcana	chr12:2,617,572-2,650,960	chr5:47,909,439-48,049,230	Null	Null	lincRNA*	lincRNA*	Null	Null	1	1	Null	Null	Yes	Null
lnc_setd1ba	chr4:1,806,612-1,815,015	chr10:43,342,780-43,356,038	chr5:123,121,050-123,159,248	chr12:122,227,701-122,245,066	lincRNA*	lincRNA*	lincRNA	lincRNA	1	1	1	1	Yes	Yes

***CAGE support**

Supplementary data S6: List of IDs of all the assembled non-coding transcripts grouped into classes based on their genomic location.

Class:LincRNA

TCONS_00000137 TCONS_00000209 TCONS_00000344 TCONS_00000345 TCONS_00000364 TCONS_00000716 TCONS_00000765 TCONS_00000906 TCONS_00000907

TCONS_00001075 TCONS_00001090 TCONS_00001166 TCONS_00001322 TCONS_00001331 TCONS_00001446 TCONS_00001520 TCONS_00002126 TCONS_00002139

TCONS_00002162 TCONS_00002175 TCONS_00002177 TCONS_00002311 TCONS_00002314 TCONS_00002407 TCONS_00003014 TCONS_00003407 TCONS_00004085

TCONS_00004105 TCONS_00004221 TCONS_00004633 TCONS_00004805 TCONS_00004832 TCONS_00004857 TCONS_00004858 TCONS_00005256 TCONS_00005357

TCONS_00005431 TCONS_00005700 TCONS_00005719 TCONS_00005751 TCONS_00005794 TCONS_00005998 TCONS_00006050 TCONS_00006209 TCONS_00006543

TCONS_00006599 TCONS_00006687 TCONS_00007003 TCONS_00007019 TCONS_00007127 TCONS_00007168 TCONS_00007274 TCONS_00007334 TCONS_00007540

TCONS_00007671 TCONS_00007923 TCONS_00007927 TCONS_00007928 TCONS_00007963 TCONS_00008376 TCONS_00008529 TCONS_00008535 TCONS_00008612

TCONS_00008661 TCONS_00009179 TCONS_00009199 TCONS_00009509 TCONS_00009582 TCONS_00009715 TCONS_00009716 TCONS_00009823 TCONS_00009947

TCONS_00010342 TCONS_00010382 TCONS_00010457 TCONS_00010461 TCONS_00010599 TCONS_00010736 TCONS_00011216 TCONS_00011437 TCONS_00011487

TCONS_00011535 TCONS_00011946 TCONS_00012001 TCONS_00012074 TCONS_00012176 TCONS_00013022 TCONS_00013175 TCONS_00013441 TCONS_00013491

TCONS_00013673 TCONS_00013674 TCONS_00013927 TCONS_00014303 TCONS_00014552 TCONS_00014553 TCONS_00014785 TCONS_00015031 TCONS_00015180

TCONS_00015399 TCONS_00015612 TCONS_00015632 TCONS_00015724 TCONS_00015727 TCONS_00015728 TCONS_00015751 TCONS_00015752 TCONS_00015787

TCONS_00015893 TCONS_00015935 TCONS_00016072 TCONS_00016262 TCONS_00016298 TCONS_00016774 TCONS_00016800 TCONS_00017021 TCONS_00017164

TCONS_00017475 TCONS_00017562 TCONS_00017620 TCONS_00018220 TCONS_00018227 TCONS_00018228 TCONS_00018229 TCONS_00018237 TCONS_00018256

TCONS_00018283 TCONS_00018668 TCONS_00018749 TCONS_00018793 TCONS_00018895 TCONS_00019194 TCONS_00019470 TCONS_00019809 TCONS_00020329

TCONS_00020487 TCONS_00020559 TCONS_00020644 TCONS_00020868 TCONS_00021537 TCONS_00021991 TCONS_00022030 TCONS_00022100 TCONS_00022115

TCONS_00022232 TCONS_00022549 TCONS_00022587 TCONS_00022898 TCONS_00023001 TCONS_00023297 TCONS_00023303 TCONS_00023571 TCONS_00023698

TCONS_00023959 TCONS_00023967 TCONS_00024417 TCONS_00024460 TCONS_00024523 TCONS_00024698 TCONS_00024842 TCONS_00025538 TCONS_00025563

TCONS_00026056 TCONS_00026057 TCONS_00026151 TCONS_00026662 TCONS_00026735 TCONS_00026736 TCONS_00026798 TCONS_00026822 TCONS_00026866

TCONS_00026963 TCONS_00027057 TCONS_00027116 TCONS_00027281 TCONS_00027312 TCONS_00027531 TCONS_00027608 TCONS_00027717 TCONS_00027738

TCONS_00027865 TCONS_00028094 TCONS_00028129 TCONS_00028134 TCONS_00028154 TCONS_00028472 TCONS_00028490 TCONS_00028685 TCONS_00028924

TCONS_00028925 TCONS_00028987 TCONS_00029215 TCONS_00029288 TCONS_00029391 TCONS_00029578 TCONS_00029630 TCONS_00029830 TCONS_00030084

TCONS_00030419 TCONS_00030420 TCONS_00030521 TCONS_00030567 TCONS_00030617 TCONS_00030670 TCONS_00030778 TCONS_00031274 TCONS_00031300

TCONS_00031329 TCONS_00031524 TCONS_00031532 TCONS_00031627 TCONS_00031712 TCONS_00031837 TCONS_00031990 TCONS_00032344 TCONS_00032407

TCONS_00032473 TCONS_00032526 TCONS_00032930 TCONS_00032960 TCONS_00033304 TCONS_00033365 TCONS_00033412 TCONS_00033627 TCONS_00033637

TCONS_00033863 TCONS_00033930 TCONS_00034066 TCONS_00034601 TCONS_00034642 TCONS_00034674 TCONS_00034702 TCONS_00035035 TCONS_00035241

TCONS_00035616 TCONS_00035673 TCONS_00035729 TCONS_00036235 TCONS_00036387 TCONS_00036471 TCONS_00036539 TCONS_00036614 TCONS_00036623

TCONS_00036922 TCONS_00037026 TCONS_00037268 TCONS_00037315 TCONS_00037613 TCONS_00037614 TCONS_00037706 TCONS_00038063 TCONS_00038292

TCONS_00038293 TCONS_00038294 TCONS_00038461 TCONS_00038508 TCONS_00038509 TCONS_00038663 TCONS_00038664 TCONS_00038758 TCONS_00038760

TCONS_00038974 TCONS_00038996 TCONS_00038997 TCONS_00039015 TCONS_00039175 TCONS_00039351 TCONS_00039374 TCONS_00039375 TCONS_00039501

TCONS_00039603 TCONS_00040235 TCONS_00040482 TCONS_00040483 TCONS_00040726 TCONS_00040825 TCONS_00040831 TCONS_00040902 TCONS_00040989

TCONS_00041282 TCONS_00041340 TCONS_00041341 TCONS_00041641 TCONS_00042124 TCONS_00042606 TCONS_00042741 TCONS_00042864 TCONS_00043175

TCONS_00043975 TCONS_00044050 TCONS_00044408 TCONS_00044557 TCONS_00044586 TCONS_00044630 TCONS_00044850 TCONS_00044997 TCONS_00045360

TCONS_00045421 TCONS_00047227 TCONS_00047556 TCONS_00047750 TCONS_00047763 TCONS_00047976 TCONS_00047977 TCONS_00047981 TCONS_00048125

TCONS_00048401 TCONS_00048792 TCONS_00049025 TCONS_00049379 TCONS_00049539 TCONS_00049852 TCONS_00050364 TCONS_00050372 TCONS_00050522

TCONS_00050523 TCONS_00050710 TCONS_00050846 TCONS_00050926 TCONS_00051436 TCONS_00051656 TCONS_00051716 TCONS_00051717 TCONS_00051800

TCONS_00051927 TCONS_00051960 TCONS_00051961 TCONS_00052075 TCONS_00052101 TCONS_00052194 TCONS_00052231 TCONS_00052440 TCONS_00052481

TCONS_00052629 TCONS_00052839 TCONS_00052892 TCONS_00052894 TCONS_00052900 TCONS_00052998 TCONS_00053182 TCONS_00053273 TCONS_00053893

TCONS_00053965 TCONS_00054027 TCONS_00054062 TCONS_00054131 TCONS_00054132 TCONS_00054174 TCONS_00054323 TCONS_00054334 TCONS_00054525

TCONS_00054567 TCONS_00054725 TCONS_00054799 TCONS_00054842 TCONS_00054899 TCONS_00054916 TCONS_00055157 TCONS_00055158 TCONS_00055236

TCONS_00055248 TCONS_00055271 TCONS_00055299 TCONS_00055331 TCONS_00055332 TCONS_00055951 TCONS_00056012 TCONS_00056404 TCONS_00056449

TCONS_00056504 TCONS_00056505 TCONS_00056619 TCONS_00057004 TCONS_00057018 TCONS_00057085 TCONS_00057214 TCONS_00057360 TCONS_00057428

TCONS_00057582 TCONS_00057637 TCONS_00057950 TCONS_00058250 TCONS_00058349 TCONS_00058359 TCONS_00058369 TCONS_00058527 TCONS_00058721

TCONS_00058945 TCONS_00058959 TCONS_00058983 TCONS_00059301 TCONS_00059303 TCONS_00059324 TCONS_00059469 TCONS_00059470 TCONS_00059476

TCONS_00059480 TCONS_00059495 TCONS_00059561 TCONS_00059562 TCONS_00059564 TCONS_00059584 TCONS_00059660 TCONS_00059832 TCONS_00059955

TCONS_00059956 TCONS_00059957 TCONS_00060097 TCONS_00060186 TCONS_00060509 TCONS_00060510 TCONS_00060603 TCONS_00060652 TCONS_00060804

TCONS_00060820 TCONS_00061062 TCONS_00061072 TCONS_00061191 TCONS_00061616 TCONS_00061757 TCONS_00062146 TCONS_00062472 TCONS_00062563

TCONS_00062614 TCONS_00062615 TCONS_00062676 TCONS_00062736 TCONS_00062769 TCONS_00063054 TCONS_00063155 TCONS_00063383 TCONS_00063384

TCONS_00063459 TCONS_00063514 TCONS_00063564 TCONS_00063591 TCONS_00063834 TCONS_00063838 TCONS_00063965 TCONS_00063992 TCONS_00064276

TCONS_00064357 TCONS_00064382 TCONS_00064489 TCONS_00064684 TCONS_00064685 TCONS_00064902 TCONS_00064928 TCONS_00065047 TCONS_00065292

TCONS_00065455 TCONS_00065508 TCONS_00065510 TCONS_00065511 TCONS_00065560 TCONS_00065750 TCONS_00065757 TCONS_00065838 TCONS_00065913

TCONS_00066038 TCONS_00066065 TCONS_00066094 TCONS_00066303 TCONS_00066327 TCONS_00066480 TCONS_00066481 TCONS_00066762 TCONS_00066954

TCONS_00067064 TCONS_00067526 TCONS_00067652 TCONS_00067971 TCONS_00067988 TCONS_00068148 TCONS_00068311 TCONS_00068651 TCONS_00068741

TCONS_00068902 TCONS_00068949 TCONS_00069456 TCONS_00069457 TCONS_00069619 TCONS_00070159 TCONS_00070578 TCONS_00070815 TCONS_00071365

TCONS_00071590 TCONS_00072077 TCONS_00072654 TCONS_00072714 TCONS_00072726 TCONS_00073242 TCONS_00073327 TCONS_00073571 TCONS_00074820

TCONS_00075325 TCONS_00075759 TCONS_00075849 TCONS_00075946 TCONS_00076478 TCONS_00076502 TCONS_00076553 TCONS_00076697 TCONS_00077091

TCONS_00077167 TCONS_00077291 TCONS_00077506 TCONS_00078599 TCONS_00078949 TCONS_00079208 TCONS_00079214 TCONS_00079632 TCONS_00079754

TCONS_00080366 TCONS_00080429 TCONS_00080656 TCONS_00080734 TCONS_00080844 TCONS_00080845 TCONS_00081180 TCONS_00081287 TCONS_00081288

TCONS_00081935 TCONS_00082735 TCONS_00082829 TCONS_00083003 TCONS_00083010 TCONS_00083161 TCONS_00083422 TCONS_00083679 TCONS_00083908

TCONS_00084074 TCONS_00084119 TCONS_00084186 TCONS_00084215 TCONS_00084221 TCONS_00084222 TCONS_00084297 TCONS_00084604 TCONS_00084973

TCONS_00084992 TCONS_00085337 TCONS_00085443 TCONS_00085567 TCONS_00085569 TCONS_00085588 TCONS_00085603 TCONS_00085708 TCONS_00085782

TCONS_00085839 TCONS_00085940 TCONS_00085945 TCONS_00085978 TCONS_00086022 TCONS_00086047 TCONS_00086233 TCONS_00086771 TCONS_00087074

TCONS_00087786 TCONS_00088706 TCONS_00089078 TCONS_00089792 TCONS_00090113 TCONS_00090680 TCONS_00090858 TCONS_00091129 TCONS_00091220

TCONS_00091373 TCONS_00091534 TCONS_00092469 TCONS_00092493 TCONS_00092499 TCONS_00092665 TCONS_00092721 TCONS_00092870 TCONS_00092878

Class:Intronic

TCONS_00000098 TCONS_00000190 TCONS_00000813 TCONS_00001214 TCONS_00001309 TCONS_00004111 TCONS_00004118 TCONS_00004119 TCONS_00004950

TCONS_00005380 TCONS_00005873 TCONS_00006015 TCONS_00007894 TCONS_00010706 TCONS_00010795 TCONS_00011383 TCONS_00013222 TCONS_00015625

TCONS_00016724 TCONS_00017187 TCONS_00017709 TCONS_00017727 TCONS_00017798 TCONS_00018346 TCONS_00018828 TCONS_00019552 TCONS_00019958

TCONS_00020036 TCONS_00020109 TCONS_00020112 TCONS_00020994 TCONS_00022419 TCONS_00022842 TCONS_00023280 TCONS_00025374 TCONS_00026956

TCONS_00027726 TCONS_00027888 TCONS_00028657 TCONS_00029130 TCONS_00032444 TCONS_00033494 TCONS_00033759 TCONS_00034278 TCONS_00035247

TCONS_00038623 TCONS_00038772 TCONS_00039879 TCONS_00040223 TCONS_00040295 TCONS_00040664 TCONS_00041026 TCONS_00041027 TCONS_00041183

TCONS_00041220 TCONS_00043916 TCONS_00044164 TCONS_00044668 TCONS_00045611 TCONS_00045615 TCONS_00045617 TCONS_00047425 TCONS_00047886

TCONS_00048133 TCONS_00048169 TCONS_00048309 TCONS_00049060 TCONS_00049064 TCONS_00049631 TCONS_00050074 TCONS_00050274 TCONS_00050569

TCONS_00050589 TCONS_00050590 TCONS_00051314 TCONS_00051641 TCONS_00051958 TCONS_00052809 TCONS_00052978 TCONS_00054004 TCONS_00054286

TCONS_00056700 TCONS_00057980 TCONS_00057981 TCONS_00058137 TCONS_00058575 TCONS_00058709 TCONS_00058818 TCONS_00059419 TCONS_00062332

TCONS_00062565 TCONS_00063378 TCONS_00063743 TCONS_00063747 TCONS_00064145 TCONS_00065300 TCONS_00067260 TCONS_00067270 TCONS_00067385

Class:Antisense

TCONS_00002755 TCONS_00002797 TCONS_00004120 TCONS_00006350 TCONS_00008008 TCONS_00009662 TCONS_00009877 TCONS_00011529 TCONS_00014123

TCONS_00016099 TCONS_00016332 TCONS_00016333 TCONS_00017303 TCONS_00020517 TCONS_00021579 TCONS_00022724 TCONS_00022912 TCONS_00024756

TCONS_00027852 TCONS_00029348 TCONS_00030386 TCONS_00032867 TCONS_00034269 TCONS_00037238 TCONS_00039248 TCONS_00040913 TCONS_00042090

TCONS_00043638 TCONS_00043681 TCONS_00049744 TCONS_00049981 TCONS_00051678 TCONS_00052979 TCONS_00053025 TCONS_00053524 TCONS_00053892

TCONS_00053973 TCONS_00053974 TCONS_00054066 TCONS_00055087 TCONS_00055089 TCONS_00055561 TCONS_00057447 TCONS_00058141 TCONS_00059417

TCONS_00059977 TCONS_00061733 TCONS_00062134 TCONS_00065255

Class:Others

TCONS_00000513 TCONS_00000759 TCONS_00000983 TCONS_00001111 TCONS_00001112 TCONS_00001164 TCONS_00001168 TCONS_00001260 TCONS_00001440

TCONS_00001568 TCONS_00002033 TCONS_00002079 TCONS_00002153 TCONS_00002498 TCONS_00002525 TCONS_00002670 TCONS_00002902 TCONS_00002905

TCONS_00003004 TCONS_00003221 TCONS_00003352 TCONS_00003476 TCONS_00003543 TCONS_00003581 TCONS_00004053 TCONS_00004093 TCONS_00004564

TCONS_00005057 TCONS_00005267 TCONS_00006162 TCONS_00006400 TCONS_00006476 TCONS_00006606 TCONS_00006612 TCONS_00006788 TCONS_00007087

TCONS_00007323 TCONS_00007820 TCONS_00007877 TCONS_00008130 TCONS_00008372 TCONS_00008639 TCONS_00008722 TCONS_00009056 TCONS_00009084

TCONS_00009107 TCONS_00009421 TCONS_00009506 TCONS_00009565 TCONS_00009973 TCONS_00010097 TCONS_00010199 TCONS_00010223 TCONS_00010227

TCONS_00010243 TCONS_00010751 TCONS_00010752 TCONS_00010874 TCONS_00011425 TCONS_00011571 TCONS_00012130 TCONS_00012189 TCONS_00012300

TCONS_00012422 TCONS_00012464 TCONS_00012503 TCONS_00013454 TCONS_00013617 TCONS_00013642 TCONS_00014026 TCONS_00014080 TCONS_00014081

TCONS_00014475 TCONS_00014938 TCONS_00014940 TCONS_00014958 TCONS_00015189 TCONS_00015297 TCONS_00015310 TCONS_00015613 TCONS_00015853

TCONS_00015924 TCONS_00015963 TCONS_00016046 TCONS_00016064 TCONS_00016139 TCONS_00016385 TCONS_00016386 TCONS_00016463 TCONS_00016910

TCONS_00016924 TCONS_00016928 TCONS_00016930 TCONS_00016985 TCONS_00016986 TCONS_00017395 TCONS_00017491 TCONS_00017739 TCONS_00018179

TCONS_00018324 TCONS_00018478 TCONS_00018513 TCONS_00018596 TCONS_00018879 TCONS_00019026 TCONS_00019564 TCONS_00019590 TCONS_00019792

TCONS_00020455 TCONS_00020627 TCONS_00020981 TCONS_00021140 TCONS_00021187 TCONS_00021413 TCONS_00021664 TCONS_00021718 TCONS_00021723

TCONS_00021933 TCONS_00021972 TCONS_00022057 TCONS_00022059 TCONS_00022089 TCONS_00022090 TCONS_00022091 TCONS_00022092 TCONS_00022093

TCONS_00022183 TCONS_00022247 TCONS_00022899 TCONS_00023066 TCONS_00023067 TCONS_00023285 TCONS_00023289 TCONS_00023513 TCONS_00023544

TCONS_00023584 TCONS_00023717 TCONS_00023789 TCONS_00023920 TCONS_00023974 TCONS_00023976 TCONS_00024158 TCONS_00024301 TCONS_00024472

TCONS_00024503 TCONS_00024575 TCONS_00024849 TCONS_00024880 TCONS_00024896 TCONS_00025018 TCONS_00025098 TCONS_00025194 TCONS_00026671

TCONS_00026702 TCONS_00026703 TCONS_00026811 TCONS_00026960 TCONS_00027099 TCONS_00027483 TCONS_00028127 TCONS_00028128 TCONS_00028162

TCONS_00028488 TCONS_00028535 TCONS_00028631 TCONS_00028713 TCONS_00028929 TCONS_00029056 TCONS_00029085 TCONS_00029191 TCONS_00029388

TCONS_00029575 TCONS_00029576 TCONS_00029708 TCONS_00029833 TCONS_00030085 TCONS_00030148 TCONS_00030273 TCONS_00030388 TCONS_00030592

TCONS_00030630 TCONS_00030751 TCONS_00030753 TCONS_00030821 TCONS_00030862 TCONS_00030866 TCONS_00031132 TCONS_00031145 TCONS_00031398

TCONS_00031422 TCONS_00031795 TCONS_00031800 TCONS_00032077 TCONS_00032370 TCONS_00032707 TCONS_00032708 TCONS_00032709 TCONS_00032753

TCONS_00032764 TCONS_00032928 TCONS_00033575 TCONS_00033579 TCONS_00033913 TCONS_00033993 TCONS_00034598 TCONS_00034723 TCONS_00035104

TCONS_00035400 TCONS_00035447 TCONS_00035558 TCONS_00035660 TCONS_00035924 TCONS_00035983 TCONS_00036133 TCONS_00036134 TCONS_00036244

TCONS_00036246 TCONS_00036436 TCONS_00036673 TCONS_00036680 TCONS_00036934 TCONS_00037229 TCONS_00037312 TCONS_00037322 TCONS_00037987

TCONS_00037991 TCONS_00038269 TCONS_00038789 TCONS_00038916 TCONS_00039135 TCONS_00039159 TCONS_00039161 TCONS_00039203 TCONS_00039829

TCONS_00040353 TCONS_00040452 TCONS_00040504 TCONS_00040809 TCONS_00040861 TCONS_00040903 TCONS_00040978 TCONS_00041099 TCONS_00041142

TCONS_00041164 TCONS_00041273 TCONS_00041336 TCONS_00041505 TCONS_00042134 TCONS_00042145 TCONS_00042254 TCONS_00042621 TCONS_00042734

TCONS_00042993 TCONS_00042997 TCONS_00043101 TCONS_00043111 TCONS_00043648 TCONS_00043837 TCONS_00043923 TCONS_00044117 TCONS_00044121

TCONS_00044123 TCONS_00044140 TCONS_00044406 TCONS_00044407 TCONS_00044446 TCONS_00044493 TCONS_00044494 TCONS_00044652 TCONS_00044776

TCONS_00044805 TCONS_00044987 TCONS_00045009 TCONS_00045012 TCONS_00045102 TCONS_00045328 TCONS_00045490 TCONS_00045574 TCONS_00046997

TCONS_00047038 TCONS_00047087 TCONS_00047100 TCONS_00047148 TCONS_00047304 TCONS_00047636 TCONS_00047998 TCONS_00048104 TCONS_00048240

TCONS_00048307 TCONS_00048373 TCONS_00048929 TCONS_00049002 TCONS_00049061 TCONS_00049137 TCONS_00049649 TCONS_00049691 TCONS_00049723

TCONS_00049859 TCONS_00049962 TCONS_00049977 TCONS_00050008 TCONS_00050009 TCONS_00050309 TCONS_00050345 TCONS_00050647 TCONS_00050942

TCONS_00051000 TCONS_00051198 TCONS_00051308 TCONS_00051671 TCONS_00051744 TCONS_00051745 TCONS_00051856 TCONS_00051996 TCONS_00052116

TCONS_00052163 TCONS_00052278 TCONS_00052483 TCONS_00052831 TCONS_00052868 TCONS_00053101 TCONS_00053285 TCONS_00053358 TCONS_00053420

TCONS_00053519 TCONS_00053700 TCONS_00053926 TCONS_00053991 TCONS_00054216 TCONS_00054364 TCONS_00054747 TCONS_00054828 TCONS_00054832

TCONS_00055553 TCONS_00055615 TCONS_00055838 TCONS_00055948 TCONS_00055956 TCONS_00055957 TCONS_00056023 TCONS_00056034 TCONS_00056049

TCONS_00056569 TCONS_00056751 TCONS_00056753 TCONS_00056842 TCONS_00056850 TCONS_00057003 TCONS_00057072 TCONS_00057423 TCONS_00057728

TCONS_00059139 TCONS_00059508 TCONS_00059883 TCONS_00059917 TCONS_00060405 TCONS_00060433 TCONS_00061033 TCONS_00061128 TCONS_00061158

TCONS_00061258 TCONS_00062069 TCONS_00062135 TCONS_00062244 TCONS_00062272 TCONS_00062385 TCONS_00062595 TCONS_00062946 TCONS_00062983

TCONS_00063506 TCONS_00063515 TCONS_00064101 TCONS_00064376 TCONS_00064377 TCONS_00064534 TCONS_00065480 TCONS_00065507 TCONS_00065614

TCONS_00065949 TCONS_00065961 TCONS_00066168 TCONS_00066198 TCONS_00066434 TCONS_00066529 TCONS_00066658 TCONS_00067010 TCONS_00067207

TCONS_00067227 TCONS_00067425 TCONS_00067582 TCONS_00068139 TCONS_00068303 TCONS_00068319 TCONS_00068333 TCONS_00068506 TCONS_00079101

Supplementary data S8: List of IDs of all the transcripts predicted to be maternal or embryonic specific during early development in tetraodon.

Class:Maternal specific transcripts

TCONS_00026727 TCONS_00021172 TCONS_00021171 TCONS_00021173 TCONS_00073691 TCONS_00070957 TCONS_00073688 TCONS_00048092 TCONS_00054938

TCONS_00054939 TCONS_00048093 TCONS_00014123 TCONS_00051109 TCONS_00026726 TCONS_00071968 TCONS_00058359 TCONS_00055454 TCONS_00055455

TCONS_00010382 TCONS_00011311 TCONS_00020719 TCONS_00011312 TCONS_00010073 TCONS_00028381 TCONS_00040623 TCONS_00052515 TCONS_00075607

TCONS_00075608 TCONS_00084028 TCONS_00038655 TCONS_00038656 TCONS_00042555 TCONS_00042554 TCONS_00040622 TCONS_00014079 TCONS_00040621

TCONS_00021751 TCONS_00064900 TCONS_00063831 TCONS_00014643 TCONS_00015821 TCONS_00044176 TCONS_00013951 TCONS_00038293 TCONS_00013304

TCONS_00038294 TCONS_00058175 TCONS_00047813 TCONS_00038292 TCONS_00035987 TCONS_00052514 TCONS_00058176 TCONS_00072282 TCONS_00021750

TCONS_00014065 TCONS_00008790 TCONS_00054772 TCONS_00017359 TCONS_00017358 TCONS_00019777 TCONS_00019778 TCONS_00018613 TCONS_00018614

TCONS_00077282 TCONS_00047218 TCONS_00053623 TCONS_00047219 TCONS_00033013 TCONS_00006028 TCONS_00000682 TCONS_00010453 TCONS_00033012

TCONS_00035222 TCONS_00035223 TCONS_00004513 TCONS_00034249 TCONS_00058358 TCONS_00001222 TCONS_00006025 TCONS_00006026 TCONS_00001221

TCONS_00051826 TCONS_00054033 TCONS_00053027 TCONS_00074031 TCONS_00045511 TCONS_00045982 TCONS_00018953 TCONS_00082354 TCONS_00045983

TCONS_00046332 TCONS_00046584 TCONS_00080235 TCONS_00000897 TCONS_00044524 TCONS_00010454 TCONS_00032163 TCONS_00038080 TCONS_00002922

TCONS_00006027 TCONS_00018952 TCONS_00071728 TCONS_00002923 TCONS_00038987 TCONS_00023799 TCONS_00017674 TCONS_00028436 TCONS_00026791

TCONS_00073242 TCONS_00023368 TCONS_00023800 TCONS_00028435 TCONS_00032249 TCONS_00023387 TCONS_00029275 TCONS_00033874 TCONS_00009535

TCONS_00029274 TCONS_00058745 TCONS_00044776 TCONS_00047055 TCONS_00048739 TCONS_00032641 TCONS_00034969 TCONS_00033875 TCONS_00012022

TCONS_00058141 TCONS_00005912 TCONS_00030325 TCONS_00057956 TCONS_00038985 TCONS_00045896 TCONS_00012295 TCONS_00005913 TCONS_00010050

TCONS_00034968 TCONS_00039246 TCONS_00041221 TCONS_00056588 TCONS_00046252 TCONS_00020860 TCONS_00011279 TCONS_00012470 TCONS_00043680

TCONS_00063415 TCONS_00063414 TCONS_00064498 TCONS_00065448 TCONS_00064497 TCONS_00065449 TCONS_00089782 TCONS_00081923 TCONS_00056810

TCONS_00010049 TCONS_00067721 TCONS_00005914 TCONS_00005047 TCONS_00067722 TCONS_00005046 TCONS_00001351 TCONS_00055443 TCONS_00055442

TCONS_00073563 TCONS_00038288 TCONS_00040214 TCONS_00023873 TCONS_00058254 TCONS_00006909 TCONS_00058255 TCONS_00023608 TCONS_00025300

TCONS_00033492 TCONS_00034633 TCONS_00057616 TCONS_00023436 TCONS_00023437 TCONS_00066569 TCONS_00068642 TCONS_00060253 TCONS_00032243

TCONS_00070055 TCONS_00023273 TCONS_00023435 TCONS_00043059 TCONS_00087132 TCONS_00061341 TCONS_00039237 TCONS_00041211 TCONS_00034635

TCONS_00061342 TCONS_00042512 TCONS_00033493 TCONS_00060254 TCONS_00063417 TCONS_00063416 TCONS_00062412 TCONS_00025049 TCONS_00047905

TCONS_00040215 TCONS_00006685 TCONS_00062411 TCONS_00050948 TCONS_00060252 TCONS_00022118 TCONS_00066695 TCONS_00023003 TCONS_00022119

TCONS_00088787 TCONS_00072187 TCONS_00021053 TCONS_00034634 TCONS_00078418 TCONS_00013057 TCONS_00032250 TCONS_00087133 TCONS_00049490

TCONS_00031855 TCONS_00029898 TCONS_00031854 TCONS_00030924 TCONS_00048423 TCONS_00035810 TCONS_00049999 TCONS_00060667 TCONS_00047506

TCONS_00061770 TCONS_00043711 TCONS_00043968 TCONS_00043712 TCONS_00049122 TCONS_00069908 TCONS_00050981 TCONS_00050982 TCONS_00050980

TCONS_00047971 TCONS_00060096 TCONS_00049582 TCONS_00047970 TCONS_00090706 TCONS_00035809 TCONS_00047969 TCONS_00024826 TCONS_00000329

TCONS_00050914 TCONS_00083183 TCONS_00058083 TCONS_00066480 TCONS_00087134 TCONS_00003362 TCONS_00047864 TCONS_00006367 TCONS_00032434

TCONS_00054980 TCONS_00004243 TCONS_00069907 TCONS_00006806 TCONS_00010159 TCONS_00064372 TCONS_00011402 TCONS_00000814 TCONS_00059254

TCONS_00063288 TCONS_00058081 TCONS_00008119 TCONS_00020604 TCONS_00037184 TCONS_00069663 TCONS_00006592 TCONS_00044394 TCONS_00058080

TCONS_00063289 TCONS_00006276 TCONS_00066130 TCONS_00007576 TCONS_00055031 TCONS_00013106 TCONS_00007577 TCONS_00006277 TCONS_00073140

TCONS_00064373 TCONS_00055378 TCONS_00056590 TCONS_00074106 TCONS_00056530 TCONS_00038602 TCONS_00039236 TCONS_00072970 TCONS_00015746

TCONS_00013213 TCONS_00063051 TCONS_00082419 TCONS_00040565 TCONS_00013107 TCONS_00066131 TCONS_00047476 TCONS_00082418 TCONS_00022645

TCONS_00014545 TCONS_00073205 TCONS_00089524 TCONS_00065159 TCONS_00066211 TCONS_00078419 TCONS_00073308 TCONS_00059983 TCONS_00047208

TCONS_00055346 TCONS_00039272 TCONS_00047209 TCONS_00065980 TCONS_00056498 TCONS_00036143 TCONS_00013313 TCONS_00013314 TCONS_00038765

TCONS_00068071 TCONS_00069612

Class:Embryonic specific transcripts

TCONS_00035125 TCONS_00035126 TCONS_00034101 TCONS_00033404 TCONS_00034573 TCONS_00046342 TCONS_00010636 TCONS_00083117 TCONS_00010635

TCONS_00042222 TCONS_00040248 TCONS_00040247 TCONS_00084220 TCONS_00028114 TCONS_00010715 TCONS_00080647 TCONS_00021479 TCONS_00022514

TCONS_00040249 TCONS_00026098 TCONS_00026408 TCONS_00021403 TCONS_00028513 TCONS_00027650 TCONS_00085571 TCONS_00025567 TCONS_00024867

TCONS_00084624 TCONS_00012469 TCONS_00065571 TCONS_00064679 TCONS_00053148 TCONS_00028514 TCONS_00027651 TCONS_00043787 TCONS_00091627

TCONS_00053149 TCONS_00082587 TCONS_00028031 TCONS_00028032 TCONS_00028033 TCONS_00033426 TCONS_00091628 TCONS_00011276 TCONS_00086026

TCONS_00082586 TCONS_00005089 TCONS_00079113 TCONS_00061610 TCONS_00061303 TCONS_00084428 TCONS_00003421 TCONS_00028833 TCONS_00028834

TCONS_00044028 TCONS_00061611 TCONS_00054134 TCONS_00030566 TCONS_00030565 TCONS_00059533 TCONS_00053150 TCONS_00088883 TCONS_00080835

TCONS_00041980 TCONS_00040498 TCONS_00039957 TCONS_00041979 TCONS_00078718 TCONS_00039956 TCONS_00087372 TCONS_00089097 TCONS_00031542

TCONS_00079212 TCONS_00042461 TCONS_00081076 TCONS_00011028 TCONS_00012265 TCONS_00061290 TCONS_00064787 TCONS_00030564 TCONS_00027648

TCONS_00042957 TCONS_00052975 TCONS_00041079 TCONS_00061609 TCONS_00067508 TCONS_00067509 TCONS_00059001 TCONS_00028511 TCONS_00061431

TCONS_00062492 TCONS_00046410 TCONS_00040892 TCONS_00062350 TCONS_00079114 TCONS_00064556 TCONS_00087709 TCONS_00011660 TCONS_00046085

TCONS_00003010 TCONS_00058714 TCONS_00058713 TCONS_00062650 TCONS_00081995 TCONS_00062349 TCONS_00061282 TCONS_00050851 TCONS_00028521

TCONS_00080324 TCONS_00078709 TCONS_00030186 TCONS_00067713 TCONS_00077674 TCONS_00086550 TCONS_00068718 TCONS_00042595 TCONS_00004852

TCONS_00040671 TCONS_00027708 TCONS_00061921 TCONS_00049422 TCONS_00060803 TCONS_00067397 TCONS_00068458 TCONS_00081947 TCONS_00064682

TCONS_00003120 TCONS_00024902 TCONS_00065574 TCONS_00028831 TCONS_00064681 TCONS_00010741 TCONS_00028030 TCONS_00028841 TCONS_00028043

TCONS_00080482 TCONS_00027662 TCONS_00081462 TCONS_00049423 TCONS_00080453 TCONS_00042411 TCONS_00061608 TCONS_00088639 TCONS_00027435

TCONS_00040450 TCONS_00078283 TCONS_00053308 TCONS_00054274 TCONS_00089834 TCONS_00040893 TCONS_00081077 TCONS_00090461 TCONS_00090462

TCONS_00081946 TCONS_00026093 TCONS_00026404 TCONS_00026403 TCONS_00026092 TCONS_00082781 TCONS_00046837 TCONS_00046945 TCONS_00067140

TCONS_00048986 TCONS_00003309 TCONS_00091949 TCONS_00003310 TCONS_00084906 TCONS_00078798 TCONS_00089589 TCONS_00026094 TCONS_00091623

TCONS_00081879 TCONS_00081880 TCONS_00046836 TCONS_00010989 TCONS_00037254 TCONS_00080452 TCONS_00065749 TCONS_00068356 TCONS_00002315

TCONS_00089835 TCONS_00046795 TCONS_00084425 TCONS_00064114 TCONS_00064905 TCONS_00067296 TCONS_00085584 TCONS_00046907 TCONS_00036607

TCONS_00087017 TCONS_00067297 TCONS_00067063 TCONS_00068151 TCONS_00063966 TCONS_00022156 TCONS_00078939 TCONS_00030627 TCONS_00012525

TCONS_00026459 TCONS_00026161 TCONS_00011347 TCONS_00068216 TCONS_00046344 TCONS_00027661 TCONS_00011337 TCONS_00031577 TCONS_00011338

TCONS_00055863 TCONS_00057023 TCONS_00055864 TCONS_00078940 TCONS_00077714 TCONS_00057024 TCONS_00086017 TCONS_00083907 TCONS_00084562

TCONS_00088749 TCONS_00046343 TCONS_00040705 TCONS_00082346 TCONS_00090115 TCONS_00028930 TCONS_00025602 TCONS_00068150 TCONS_00062484

TCONS_00062485 TCONS_00082347 TCONS_00028170 TCONS_00064680 TCONS_00087265 TCONS_00058825 TCONS_00085706 TCONS_00091239 TCONS_00046782

TCONS_00086581 TCONS_00027913 TCONS_00027912 TCONS_00083150 TCONS_00084192 TCONS_00089397 TCONS_00002195 TCONS_00043088 TCONS_00041241

TCONS_00004993 TCONS_00031678 TCONS_00040229 TCONS_00081464 TCONS_00084199 TCONS_00042206 TCONS_00081276 TCONS_00083487 TCONS_00082145

TCONS_00089961 TCONS_00042207 TCONS_00087264 TCONS_00041140 TCONS_00012101 TCONS_00049420 TCONS_00012102 TCONS_00018177 TCONS_00091455

TCONS_00003306 TCONS_00042416 TCONS_00040459 TCONS_00080829 TCONS_00085832 TCONS_00080827 TCONS_00080627 TCONS_00010687 TCONS_00055862

TCONS_00088874 TCONS_00061289 TCONS_00011959 TCONS_00021856 TCONS_00022774 TCONS_00078235 TCONS_00080828 TCONS_00078356 TCONS_00003307

TCONS_00084189 TCONS_00082230 TCONS_00062085 TCONS_00090042 TCONS_00068357 TCONS_00036306 TCONS_00026293 TCONS_00064868 TCONS_00019456

TCONS_00081153 TCONS_00018175 TCONS_00068108 TCONS_00027911 TCONS_00018176 TCONS_00003311 TCONS_00082212 TCONS_00067036 TCONS_00003631

TCONS_00003632 TCONS_00005289 TCONS_00058596 TCONS_00036997 TCONS_00019909 TCONS_00059182 TCONS_00056401 TCONS_00019811 TCONS_00059181

TCONS_00025462 TCONS_00089149 TCONS_00056400 TCONS_00024728 TCONS_00033195 TCONS_00084450 TCONS_00019532 TCONS_00019533 TCONS_00045332

TCONS_00028727 TCONS_00028726 TCONS_00021993 TCONS_00083762 TCONS_00033391 TCONS_00010842 TCONS_00021992 TCONS_00010841 TCONS_00010854

TCONS_00018774 TCONS_00034565 TCONS_00065559 TCONS_00049387 TCONS_00084200 TCONS_00030733 TCONS_00043855 TCONS_00067586 TCONS_00067585

TCONS_00030610 TCONS_00056443 TCONS_00082166 TCONS_00018775 TCONS_00044080 TCONS_00064154 TCONS_00004992 TCONS_00042126 TCONS_00040122

TCONS_00026253 TCONS_00027835 TCONS_00077715 TCONS_00085039 TCONS_00049843 TCONS_00049844 TCONS_00068628 TCONS_00068629 TCONS_00087369

TCONS_00064155 TCONS_00086012 TCONS_00022901 TCONS_00022900 TCONS_00065180 TCONS_00025566 TCONS_00090602 TCONS_00058811 TCONS_00083007

TCONS_00018773 TCONS_00027982 TCONS_00036266 TCONS_00081973 TCONS_00051235 TCONS_00081972 TCONS_00027981 TCONS_00061417 TCONS_00050829

TCONS_00040120 TCONS_00040365 TCONS_00067360 TCONS_00058809 TCONS_00080483 TCONS_00042325 TCONS_00026448 TCONS_00026145 TCONS_00026449

TCONS_00003140 TCONS_00090030 TCONS_00083139 TCONS_00040123 TCONS_00058810 TCONS_00040579 TCONS_00052944 TCONS_00033885 TCONS_00024866

TCONS_00020091 TCONS_00049126 TCONS_00025024 TCONS_00085594 TCONS_00033886 TCONS_00085787 TCONS_00007140 TCONS_00024147 TCONS_00025025

TCONS_00060974 TCONS_00085040 TCONS_00045331 TCONS_00091461 TCONS_00033950 TCONS_00061919 TCONS_00042521 TCONS_00053202 TCONS_00035016

TCONS_00090459 TCONS_00033402 TCONS_00050640 TCONS_00033949 TCONS_00059545 TCONS_00040121 TCONS_00034571 TCONS_00060802 TCONS_00036305

TCONS_00068769 TCONS_00067774 TCONS_00061489 TCONS_00031099 TCONS_00034572 TCONS_00062547 TCONS_00062546 TCONS_00040727 TCONS_00064785

TCONS_00085049 TCONS_00040728 TCONS_00081594 TCONS_00031100 TCONS_00059374 TCONS_00059373 TCONS_00058808 TCONS_00084212 TCONS_00059016

TCONS_00031098 TCONS_00035017 TCONS_00049109 TCONS_00049108 TCONS_00058806 TCONS_00004383 TCONS_00033415 TCONS_00082667 TCONS_00033416

TCONS_00046532 TCONS_00046531 TCONS_00030039 TCONS_00009115 TCONS_00084449 TCONS_00007982 TCONS_00045071 TCONS_00045529 TCONS_00033414

TCONS_00049388 TCONS_00030040 TCONS_00078535 TCONS_00082779 TCONS_00077897 TCONS_00079284 TCONS_00033951 TCONS_00061416 TCONS_00050520

TCONS_00082886 TCONS_00088371 TCONS_00071561 TCONS_00061418 TCONS_00088372 TCONS_00027910 TCONS_00081395 TCONS_00071560 TCONS_00031531

TCONS_00085231 TCONS_00056506 TCONS_00079234 TCONS_00068767 TCONS_00091522 TCONS_00058807 TCONS_00030557 TCONS_00030556 TCONS_00031530

TCONS_00084282 TCONS_00061419 TCONS_00002316 TCONS_00004238 TCONS_00040134 TCONS_00037697 TCONS_00030740 TCONS_00037696 TCONS_00010790

TCONS_00087237 TCONS_00078536 TCONS_00083761 TCONS_00067772 TCONS_00084281 TCONS_00060801 TCONS_00090604 TCONS_00007784 TCONS_00053020

TCONS_00091521 TCONS_00053887 TCONS_00064668 TCONS_00064669 TCONS_00046530 TCONS_00033561 TCONS_00014418 TCONS_00046267 TCONS_00030555

TCONS_00079608 TCONS_00083145 TCONS_00033959 TCONS_00068759 TCONS_00004076 TCONS_00033971 TCONS_00081299 TCONS_00040133 TCONS_00053457

TCONS_00002081 TCONS_00033970 TCONS_00081342 TCONS_00067437 TCONS_00002082 TCONS_00084213 TCONS_00025346 TCONS_00079101 TCONS_00067758

TCONS_00042138 TCONS_00004329 TCONS_00064363 TCONS_00064521 TCONS_00083506 TCONS_00030755 TCONS_00030739 TCONS_00061607 TCONS_00057196

TCONS_00033754 TCONS_00090603 TCONS_00052832 TCONS_00024552 TCONS_00033755 TCONS_00065460 TCONS_00041369 TCONS_00033756 TCONS_00062648

TCONS_00019920 TCONS_00026451 TCONS_00041368 TCONS_00062649 TCONS_00033403 TCONS_00082826 TCONS_00082827 TCONS_00057195 TCONS_00082211

TCONS_00060980 TCONS_00090599 TCONS_00040871 TCONS_00052941 TCONS_00002430 TCONS_00007051 TCONS_00026146 TCONS_00015793 TCONS_00082780

TCONS_00061606 TCONS_00024480 TCONS_00018794 TCONS_00008344 TCONS_00081343 TCONS_00082563 TCONS_00026147 TCONS_00050620 TCONS_00090292

TCONS_00042137 TCONS_00064522 TCONS_00081449 TCONS_00012234 TCONS_00042875 TCONS_00055991 TCONS_00053676 TCONS_00082733 TCONS_00055990

TCONS_00033958 TCONS_00008345 TCONS_00018459 TCONS_00090876 TCONS_00040985 TCONS_00044808 TCONS_00034872 TCONS_00024553 TCONS_00049538

TCONS_00081539 TCONS_00090875 TCONS_00005303 TCONS_00019661 TCONS_00053675 TCONS_00034873 TCONS_00092061 TCONS_00014862 TCONS_00090029

TCONS_00015993 TCONS_00003671 TCONS_00034875 TCONS_00081396 TCONS_00034874 TCONS_00014749 TCONS_00034936 TCONS_00003653 TCONS_00062499

TCONS_00002053 TCONS_00018440 TCONS_00024146 TCONS_00014750 TCONS_00092499 TCONS_00043979 TCONS_00035092 TCONS_00018532 TCONS_00062498

TCONS_00090460 TCONS_00033824 TCONS_00043978 TCONS_00083098 TCONS_00036411 TCONS_00078258 TCONS_00086997 TCONS_00015910 TCONS_00091895

TCONS_00002432 TCONS_00002433 TCONS_00082226 TCONS_00090037 TCONS_00002955 TCONS_00087821 TCONS_00083628 TCONS_00086591 TCONS_00046086

TCONS_00081303 TCONS_00088190 TCONS_00082225 TCONS_00041288 TCONS_00041287 TCONS_00084101 TCONS_00036629 TCONS_00049446 TCONS_00088562

TCONS_00028168 TCONS_00030734 TCONS_00090769 TCONS_00003176 TCONS_00086725 TCONS_00062195 TCONS_00084269 TCONS_00068627 TCONS_00011168

TCONS_00078157 TCONS_00002077 TCONS_00007684 TCONS_00008876 TCONS_00061097 TCONS_00040704 TCONS_00008877 TCONS_00091707 TCONS_00014396

TCONS_00024253 TCONS_00025118 TCONS_00091516 TCONS_00091131 TCONS_00003605 TCONS_00089815 TCONS_00092164 TCONS_00065253 TCONS_00089816

TCONS_00067656 TCONS_00067657 TCONS_00018658 TCONS_00091515 TCONS_00028929 TCONS_00014395 TCONS_00083160 TCONS_00080333 TCONS_00082316

TCONS_00043119 TCONS_00026163 TCONS_00021663 TCONS_00014606 TCONS_00026164 TCONS_00073131 TCONS_00011581 TCONS_00082319 TCONS_00037104

TCONS_00056505 TCONS_00036428 TCONS_00004075 TCONS_00086588 TCONS_00056504 TCONS_00081159 TCONS_00077721 TCONS_00040928 TCONS_00083129

TCONS_00067756 TCONS_00052834 TCONS_00079738 TCONS_00087216 TCONS_00080719 TCONS_00082320 TCONS_00082321 TCONS_00078512 TCONS_00078513

TCONS_00054578 TCONS_00057067 TCONS_00033812 TCONS_00055943 TCONS_00010791 TCONS_00053677 TCONS_00081824 TCONS_00083215 TCONS_00080718

TCONS_00027434 TCONS_00052943 TCONS_00082318 TCONS_00005021 TCONS_00061281 TCONS_00081825 TCONS_00083262 TCONS_00090730 TCONS_00088799

TCONS_00007034 TCONS_00037633 TCONS_00024481 TCONS_00084285 TCONS_00027926 TCONS_00052942 TCONS_00083263 TCONS_00041240 TCONS_00080845

TCONS_00077720 TCONS_00053983 TCONS_00015909 TCONS_00091523 TCONS_00002499 TCONS_00004375 TCONS_00046573 TCONS_00090558 TCONS_00046314

TCONS_00079249 TCONS_00061675 TCONS_00035090 TCONS_00082939 TCONS_00060973 TCONS_00037724 TCONS_00016216 TCONS_00003328 TCONS_00015100

TCONS_00081606 TCONS_00009062 TCONS_00008582 TCONS_00046268 TCONS_00002076 TCONS_00033983 TCONS_00026216 TCONS_00088760 TCONS_00025292

TCONS_00049444 TCONS_00064798 TCONS_00031278 TCONS_00084424 TCONS_00017992 TCONS_00078548 TCONS_00077694 TCONS_00088848 TCONS_00089286

TCONS_00030245 TCONS_00035034 TCONS_00007317 TCONS_00083717 TCONS_00028403 TCONS_00027547 TCONS_00028402 TCONS_00015099 TCONS_00007886

TCONS_00078205 TCONS_00060971 TCONS_00026462 TCONS_00024145 TCONS_00090686 TCONS_00088563 TCONS_00036728 TCONS_00079912 TCONS_00049443

TCONS_00049447 TCONS_00080844 TCONS_00078206 TCONS_00052831 TCONS_00065671 TCONS_00049213 TCONS_00083582 TCONS_00030046 TCONS_00081319

TCONS_00007671 TCONS_00004967 TCONS_00004968 TCONS_00037103 TCONS_00088675 TCONS_00002434 TCONS_00044685 TCONS_00036427 TCONS_00083583

TCONS_00026168 TCONS_00053984 TCONS_00002570 TCONS_00041294 TCONS_00088284 TCONS_00091173 TCONS_00081448 TCONS_00089387 TCONS_00036726

TCONS_00064031 TCONS_00030048 TCONS_00007414 TCONS_00043879 TCONS_00091172 TCONS_00091199 TCONS_00035091 TCONS_00007833 TCONS_00077348

TCONS_00024864 TCONS_00040462 TCONS_00054577 TCONS_00084645 TCONS_00081287 TCONS_00003269 TCONS_00042417 TCONS_00022908 TCONS_00080430

TCONS_00040461 TCONS_00042418 TCONS_00049985 TCONS_00079913 TCONS_00067092 TCONS_00090681 TCONS_00083213 TCONS_00019277 TCONS_00080288

TCONS_00064032 TCONS_00079911 TCONS_00007787 TCONS_00007415 TCONS_00008676 TCONS_00008675 TCONS_00089591 TCONS_00027654 TCONS_00079600

TCONS_00065710 TCONS_00061756 TCONS_00066743 TCONS_00015202 TCONS_00016290 TCONS_00064169 TCONS_00011669 TCONS_00015201 TCONS_00046839

TCONS_00016291 TCONS_00084360 TCONS_00011670 TCONS_00080457 TCONS_00051323 TCONS_00042415 TCONS_00077347 TCONS_00048988 TCONS_00030324

TCONS_00079974 TCONS_00064170 TCONS_00061421 TCONS_00046575 TCONS_00058527 TCONS_00056084 TCONS_00079377 TCONS_00065471 TCONS_00084847

TCONS_00028132 TCONS_00040457 TCONS_00040366 TCONS_00044873 TCONS_00062487 TCONS_00079599 TCONS_00088286 TCONS_00087764 TCONS_00014610

TCONS_00036688 TCONS_00088300 TCONS_00086269 TCONS_00007048 TCONS_00011737 TCONS_00067094 TCONS_00077349 TCONS_00064117 TCONS_00064119

TCONS_00078547 TCONS_00084846 TCONS_00081970 TCONS_00022000 TCONS_00048887 TCONS_00064504 TCONS_00080926 TCONS_00072410 TCONS_00002435

TCONS_00081971 TCONS_00018793 TCONS_00012580 TCONS_00028737 TCONS_00065452 TCONS_00065611 TCONS_00014748 TCONS_00010986 TCONS_00090963

TCONS_00030323 TCONS_00025347 TCONS_00040434 TCONS_00090962 TCONS_00079603 TCONS_00064857 TCONS_00081428 TCONS_00007617 TCONS_00064760

TCONS_00044953 TCONS_00078585 TCONS_00007615 TCONS_00011418 TCONS_00008822 TCONS_00049442 TCONS_00081427 TCONS_00008823 TCONS_00007614

TCONS_00002521 TCONS_00010844 TCONS_00086270 TCONS_00060800 TCONS_00058914 TCONS_00002330 TCONS_00061235 TCONS_00061234 TCONS_00061236

TCONS_00040251 TCONS_00040431 TCONS_00050865 TCONS_00064759 TCONS_00064118 TCONS_00007811 TCONS_00007509 TCONS_00036121 TCONS_00073130

TCONS_00027617 TCONS_00008315 TCONS_00034001 TCONS_00048886 TCONS_00007001 TCONS_00008668 TCONS_00082315 TCONS_00011419 TCONS_00062301

TCONS_00046421 TCONS_00079704 TCONS_00004183 TCONS_00064004 TCONS_00061422 TCONS_00064116 TCONS_00033928 TCONS_00040433 TCONS_00082714

TCONS_00034043 TCONS_00045064 TCONS_00019919 TCONS_00020186 TCONS_00062302 TCONS_00041194 TCONS_00024556 TCONS_00063789 TCONS_00045392

TCONS_00004060 TCONS_00045525 TCONS_00086001 TCONS_00028430 TCONS_00083428 TCONS_00090878 TCONS_00007616 TCONS_00021401 TCONS_00090028

TCONS_00045524 TCONS_00011916 TCONS_00007094 TCONS_00028133 TCONS_00028899 TCONS_00010634 TCONS_00067561 TCONS_00080525 TCONS_00088690

TCONS_00062678 TCONS_00050421 TCONS_00083214 TCONS_00083212 TCONS_00007510 TCONS_00084356 TCONS_00034045 TCONS_00002943 TCONS_00012581

TCONS_00049204 TCONS_00012582 TCONS_00050275 TCONS_00036511 TCONS_00040406 TCONS_00084549 TCONS_00064115 TCONS_00090093 TCONS_00090095

TCONS_00086589 TCONS_00083192 TCONS_00030269 TCONS_00020455 TCONS_00014969 TCONS_00045176 TCONS_00041359 TCONS_00084012 TCONS_00081958

TCONS_00081957 TCONS_00010943 TCONS_00007264 TCONS_00079358 TCONS_00010942 TCONS_00002055 TCONS_00089804 TCONS_00008537 TCONS_00027908

TCONS_00081607 TCONS_00077695 TCONS_00039936 TCONS_00007686 TCONS_00045411 TCONS_00044893 TCONS_00067560 TCONS_00061633 TCONS_00080821

TCONS_00004492 TCONS_00064244 TCONS_00014970 TCONS_00059180 TCONS_00014408 TCONS_00078603 TCONS_00018460 TCONS_00067090 TCONS_00067091

TCONS_00045638 TCONS_00084272 TCONS_00028092 TCONS_00087283 TCONS_00087858 TCONS_00026169 TCONS_00018839 TCONS_00042414 TCONS_00042773

TCONS_00014858 TCONS_00010840 TCONS_00015989 TCONS_00058595 TCONS_00087182 TCONS_00078481 TCONS_00082478 TCONS_00007358 TCONS_00086447

TCONS_00040432 TCONS_00010688 TCONS_00008924 TCONS_00079116 TCONS_00077578 TCONS_00083757 TCONS_00067231 TCONS_00008626 TCONS_00054825

TCONS_00048685 TCONS_00040429 TCONS_00079670 TCONS_00083089 TCONS_00039854 TCONS_00010931 TCONS_00087710 TCONS_00079141 TCONS_00040855

TCONS_00002456 TCONS_00083118 TCONS_00090656 TCONS_00056690 TCONS_00088090 TCONS_00053685 TCONS_00044964 TCONS_00044358 TCONS_00014968

TCONS_00015200 TCONS_00091323 TCONS_00007727 TCONS_00082715 TCONS_00076124 TCONS_00021452 TCONS_00079936 TCONS_00079526 TCONS_00084619

TCONS_00084620 TCONS_00083640 TCONS_00067757 TCONS_00002050 TCONS_00052968 TCONS_00064009 TCONS_00052969 TCONS_00004352 TCONS_00080822

TCONS_00078355 TCONS_00018791 TCONS_00089342 TCONS_00012190 TCONS_00027551 TCONS_00040250 TCONS_00061632 TCONS_00022419 TCONS_00011520

TCONS_00040407 TCONS_00040901 TCONS_00023035 TCONS_00078000 TCONS_00061702 TCONS_00080590 TCONS_00081558 TCONS_00030735 TCONS_00080499

TCONS_00041033 TCONS_00033977 TCONS_00035030 TCONS_00061701 TCONS_00079775 TCONS_00035803 TCONS_00080332 TCONS_00042529 TCONS_00081297

TCONS_00034044 TCONS_00008673 TCONS_00002470 TCONS_00004354 TCONS_00075892 TCONS_00015067 TCONS_00026241 TCONS_00049438 TCONS_00062730

TCONS_00007413 TCONS_00004351 TCONS_00062677 TCONS_00086323 TCONS_00008674 TCONS_00061494 TCONS_00082732 TCONS_00041371 TCONS_00061630

TCONS_00061495 TCONS_00049123 TCONS_00065063 TCONS_00002052 TCONS_00091107 TCONS_00021859 TCONS_00004126 TCONS_00021739 TCONS_00087917

TCONS_00053261 TCONS_00022695 TCONS_00050638 TCONS_00011745 TCONS_00084416 TCONS_00004125 TCONS_00007514 TCONS_00080058 TCONS_00065057

TCONS_00036320 TCONS_00041360 TCONS_00043188 TCONS_00052093 TCONS_00089695 TCONS_00043120 TCONS_00039855 TCONS_00030209 TCONS_00061078

TCONS_00002471 TCONS_00002469 TCONS_00021858 TCONS_00028799 TCONS_00040430 TCONS_00002157 TCONS_00091508 TCONS_00089694 TCONS_00056354

TCONS_00077984 TCONS_00004353 TCONS_00012008 TCONS_00079330 TCONS_00088092 TCONS_00061493 TCONS_00026761 TCONS_00083142 TCONS_00084136

TCONS_00045065 TCONS_00007412 TCONS_00046891 TCONS_00027665 TCONS_00064245 TCONS_00064725 TCONS_00054584 TCONS_00040795 TCONS_00061079

TCONS_00083111 TCONS_00028563 TCONS_00028523 TCONS_00077433 TCONS_00070206 TCONS_00007513 TCONS_00091154 TCONS_00009004 TCONS_00012118

TCONS_00077579 TCONS_00021101 TCONS_00079210 TCONS_00079211 TCONS_00027715 TCONS_00021498 TCONS_00003094 TCONS_00033284 TCONS_00008878

TCONS_00040460 TCONS_00024557 TCONS_00056249 TCONS_00018509 TCONS_00083784 TCONS_00037053 TCONS_00056480 TCONS_00024595 TCONS_00084818

TCONS_00049041 TCONS_00018840 TCONS_00068580 TCONS_00067527 TCONS_00022527 TCONS_00024594 TCONS_00034144 TCONS_00042270 TCONS_00079527

TCONS_00082153 TCONS_00025377 TCONS_00042775 TCONS_00015227 TCONS_00004831 TCONS_00033413 TCONS_00003095 TCONS_00083516 TCONS_00079165

TCONS_00042776 TCONS_00018510 TCONS_00002468 TCONS_00002520 TCONS_00040311 TCONS_00042271 TCONS_00020188 TCONS_00077580 TCONS_00002051

TCONS_00040797 TCONS_00084056 TCONS_00067627 TCONS_00024788 TCONS_00067626 TCONS_00061193 TCONS_00091454 TCONS_00034225 TCONS_00003624

TCONS_00078343 TCONS_00024911 TCONS_00025612 TCONS_00078800 TCONS_00015225 TCONS_00084190 TCONS_00041289 TCONS_00079174 TCONS_00088285

TCONS_00015456 TCONS_00004127 TCONS_00042027 TCONS_00035155 TCONS_00091896 TCONS_00012119 TCONS_00083905 TCONS_00089386 TCONS_00005339

TCONS_00062272 TCONS_00079327 TCONS_00011215 TCONS_00030268 TCONS_00062184 TCONS_00084934 TCONS_00018670 TCONS_00014919 TCONS_00080050

TCONS_00004184 TCONS_00002350 TCONS_00003096 TCONS_00016099 TCONS_00091486 TCONS_00064006 TCONS_00019107 TCONS_00007725 TCONS_00091485

TCONS_00026369 TCONS_00061629 TCONS_00003702 TCONS_00007405 TCONS_00007382 TCONS_00083131 TCONS_00059416 TCONS_00016319 TCONS_00064246

TCONS_00016318 TCONS_00020453 TCONS_00057695 TCONS_00063972 TCONS_00085938 TCONS_00034178 TCONS_00082129 TCONS_00020452 TCONS_00031304

TCONS_00018508 TCONS_00031255 TCONS_00031305 TCONS_00053652 TCONS_00066596 TCONS_00003295 TCONS_00040796 TCONS_00064030 TCONS_00011004

TCONS_00084418 TCONS_00002687 TCONS_00028431 TCONS_00024596 TCONS_00019106 TCONS_00049440 TCONS_00040859 TCONS_00056444 TCONS_00062675

TCONS_00064536 TCONS_00058867 TCONS_00031256 TCONS_00086324 TCONS_00011005 TCONS_00041370 TCONS_00061363 TCONS_00010843 TCONS_00064806

TCONS_00080069 TCONS_00080595 TCONS_00055843 TCONS_00083034 TCONS_00020011 TCONS_00011343 TCONS_00067682 TCONS_00083169 TCONS_00028488

TCONS_00086343 TCONS_00026048 TCONS_00062300 TCONS_00004747 TCONS_00002351 TCONS_00011344 TCONS_00091745 TCONS_00092057 TCONS_00003332

TCONS_00087983 TCONS_00011219 TCONS_00037647 TCONS_00014689 TCONS_00091267 TCONS_00091268 TCONS_00002216 TCONS_00034269 TCONS_00080589

TCONS_00080654 TCONS_00033712 TCONS_00002984 TCONS_00025311 TCONS_00065022 TCONS_00034289 TCONS_00080938 TCONS_00065058 TCONS_00050858

TCONS_00002983 TCONS_00084897 TCONS_00015226 TCONS_00052811 TCONS_00082477 TCONS_00052810 TCONS_00045395 TCONS_00034268 TCONS_00034123

TCONS_00010689 TCONS_00036999 TCONS_00018887 TCONS_00022162 TCONS_00022163 TCONS_00049427 TCONS_00055866 TCONS_00088960 TCONS_00018900

TCONS_00010707 TCONS_00027885 TCONS_00010716 TCONS_00091868 TCONS_00083951 TCONS_00083952 TCONS_00053875 TCONS_00024341 TCONS_00080937

TCONS_00053873 TCONS_00053874 TCONS_00088773 TCONS_00077581 TCONS_00065509 TCONS_00088301 TCONS_00027987 TCONS_00007383 TCONS_00046255

TCONS_00046593 TCONS_00082130 TCONS_00027625 TCONS_00002310 TCONS_00079173 TCONS_00064285 TCONS_00089946 TCONS_00037663 TCONS_00084221

TCONS_00007705 TCONS_00040858 TCONS_00019105 TCONS_00067334 TCONS_00078541 TCONS_00087778 TCONS_00007704 TCONS_00049668 TCONS_00082131

TCONS_00055993 TCONS_00041884 TCONS_00090531 TCONS_00050868 TCONS_00065670 TCONS_00081045 TCONS_00049880 TCONS_00051257 TCONS_00079525

TCONS_00053876 TCONS_00080696 TCONS_00068059 TCONS_00027886 TCONS_00028709 TCONS_00080492 TCONS_00046254 TCONS_00084819 TCONS_00036235

TCONS_00090866 TCONS_00007793 TCONS_00066993 TCONS_00012418 TCONS_00040351 TCONS_00008644 TCONS_00021482 TCONS_00064805 TCONS_00079597

TCONS_00084985 TCONS_00068392 TCONS_00011392 TCONS_00040040 TCONS_00082888 TCONS_00012416 TCONS_00012419 TCONS_00036698 TCONS_00008907

TCONS_00057527 TCONS_00082997 TCONS_00086364 TCONS_00067148 TCONS_00068225 TCONS_00068391 TCONS_00079937 TCONS_00036697 TCONS_00056518

TCONS_00050869 TCONS_00036309 TCONS_00078781 TCONS_00030778 TCONS_00091975 TCONS_00024342 TCONS_00036308 TCONS_00007644 TCONS_00078207

TCONS_00050548 TCONS_00024173 TCONS_00024351 TCONS_00025115 TCONS_00092393 TCONS_00084550 TCONS_00008416 TCONS_00008844 TCONS_00083517

TCONS_00012417 TCONS_00077435 TCONS_00014713 TCONS_00024554 TCONS_00042677 TCONS_00068223 TCONS_00051157 TCONS_00068224 TCONS_00024399

TCONS_00004258 TCONS_00011216 TCONS_00040860 TCONS_00087828 TCONS_00033995 TCONS_00057526 TCONS_00022775 TCONS_00079176 TCONS_00037695

TCONS_00024508 TCONS_00079459 TCONS_00089287 TCONS_00024912 TCONS_00021411 TCONS_00025310 TCONS_00015877 TCONS_00021951 TCONS_00045368

TCONS_00078051 TCONS_00009445 TCONS_00075143 TCONS_00024507 TCONS_00026227 TCONS_00079220 TCONS_00019102 TCONS_00087074 TCONS_00022765

TCONS_00082547 TCONS_00026497 TCONS_00049754 TCONS_00087435 TCONS_00022776 TCONS_00090661 TCONS_00077410 TCONS_00065507 TCONS_00084506

TCONS_00024328 TCONS_00021845 TCONS_00034301 TCONS_00052998 TCONS_00025613 TCONS_00034507 TCONS_00075328 TCONS_00031712 TCONS_00035041

TCONS_00002381 TCONS_00081623 TCONS_00040435 TCONS_00077799 TCONS_00056481 TCONS_00044990 TCONS_00068542 TCONS_00019263 TCONS_00035077

TCONS_00077958 TCONS_00081619 TCONS_00002768 TCONS_00062299 TCONS_00012420 TCONS_00061233 TCONS_00009189 TCONS_00077457 TCONS_00088466

TCONS_00008908 TCONS_00002382 TCONS_00037435 TCONS_00033932 TCONS_00037589 TCONS_00067476 TCONS_00040842 TCONS_00056280 TCONS_00026049

TCONS_00046089 TCONS_00083138 TCONS_00089146 TCONS_00007796 TCONS_00044848 TCONS_00004292 TCONS_00007795 TCONS_00049838 TCONS_00079291

TCONS_00039386 TCONS_00068543 TCONS_00077505 TCONS_00031245 TCONS_00061364 TCONS_00035050 TCONS_00048987 TCONS_00002543 TCONS_00049839

TCONS_00027830 TCONS_00081182 TCONS_00045265 TCONS_00003464 TCONS_00005136 TCONS_00084373 TCONS_00002544 TCONS_00035049 TCONS_00083407

TCONS_00065064 TCONS_00083906 TCONS_00010706 TCONS_00040367 TCONS_00080261 TCONS_00017968 TCONS_00041293 TCONS_00082976 TCONS_00083168

TCONS_00088116 TCONS_00081798 TCONS_00081282 TCONS_00079841 TCONS_00090868 TCONS_00006652 TCONS_00007967 TCONS_00073406 TCONS_00089252

TCONS_00010845 TCONS_00041842 TCONS_00057158 TCONS_00045072 TCONS_00049453 TCONS_00063973 TCONS_00065023 TCONS_00024495 TCONS_00024248

TCONS_00065024 TCONS_00084955 TCONS_00009102 TCONS_00077402 TCONS_00049104 TCONS_00040352 TCONS_00084710 TCONS_00052956 TCONS_00046526

TCONS_00078750 TCONS_00087407 TCONS_00056056 TCONS_00091802 TCONS_00021481 TCONS_00061353 TCONS_00043801 TCONS_00011214 TCONS_00083161

TCONS_00088081 TCONS_00008461 TCONS_00036310 TCONS_00082219 TCONS_00079662 TCONS_00043983 TCONS_00079661 TCONS_00011221 TCONS_00044875

TCONS_00008964 TCONS_00042197 TCONS_00082970 TCONS_00078138 TCONS_00052997 TCONS_00082157 TCONS_00081631 TCONS_00057157 TCONS_00080183

TCONS_00008923 TCONS_00004291 TCONS_00046843 TCONS_00041242 TCONS_00079842 TCONS_00040681 TCONS_00078052 TCONS_00055047 TCONS_00078137

TCONS_00031527 TCONS_00079753 TCONS_00002911 TCONS_00007862 TCONS_00016413 TCONS_00037636 TCONS_00009040 TCONS_00036515 TCONS_00084155

TCONS_00017436 TCONS_00042602 TCONS_00077817 TCONS_00012573 TCONS_00091635 TCONS_00040682 TCONS_00079524 TCONS_00022899 TCONS_00048984

TCONS_00011025 TCONS_00004906 TCONS_00007726 TCONS_00082968 TCONS_00014898 TCONS_00079523 TCONS_00027609 TCONS_00084443 TCONS_00082391

TCONS_00046525 TCONS_00036514 TCONS_00065056 TCONS_00083144 TCONS_00080673 TCONS_00012263 TCONS_00026229 TCONS_00083048 TCONS_00026228

TCONS_00040509 TCONS_00043053 TCONS_00080068 TCONS_00092469 TCONS_00086662 TCONS_00037725 TCONS_00026498 TCONS_00087152 TCONS_00086337

TCONS_00004767 TCONS_00003009 TCONS_00020096 TCONS_00042366 TCONS_00042367 TCONS_00068604 TCONS_00012822 TCONS_00012821 TCONS_00008909

TCONS_00011865 TCONS_00080182 TCONS_00002383 TCONS_00086946 TCONS_00089132 TCONS_00041201 TCONS_00056484 TCONS_00082645 TCONS_00011383

TCONS_00043984 TCONS_00067771 TCONS_00005057 TCONS_00040802 TCONS_00003202 TCONS_00011378 TCONS_00084486 TCONS_00083561 TCONS_00090986

TCONS_00046180 TCONS_00061045 TCONS_00054119 TCONS_00034799 TCONS_00049334 TCONS_00064255 TCONS_00080555 TCONS_00083096 TCONS_00011719

TCONS_00091666 TCONS_00037737 TCONS_00040473 TCONS_00054576 TCONS_00056622 TCONS_00049723 TCONS_00082220 TCONS_00003377 TCONS_00030567

TCONS_00056414 TCONS_00067238 TCONS_00090628 TCONS_00019507 TCONS_00090356 TCONS_00036301 TCONS_00071444 TCONS_00003218 TCONS_00088082

TCONS_00079910 TCONS_00008666 TCONS_00010792 TCONS_00078437 TCONS_00087348 TCONS_00033570 TCONS_00034718 TCONS_00049693 TCONS_00071209

TCONS_00060896 TCONS_00002217 TCONS_00033569 TCONS_00034717 TCONS_00041448 TCONS_00004185 TCONS_00028749 TCONS_00040218 TCONS_00056413

TCONS_00087032 TCONS_00078045 TCONS_00022172 TCONS_00089540 TCONS_00011720 TCONS_00087985 TCONS_00086922 TCONS_00047139 TCONS_00083970

TCONS_00007026 TCONS_00040436 TCONS_00019264 TCONS_00086920 TCONS_00086923 TCONS_00048813 TCONS_00025212 TCONS_00048812 TCONS_00086402

TCONS_00024398 TCONS_00062027 TCONS_00077883 TCONS_00079222 TCONS_00086921 TCONS_00002384 TCONS_00062028 TCONS_00080785 TCONS_00027932

TCONS_00054464 TCONS_00023491 TCONS_00083641 TCONS_00071208 TCONS_00017969 TCONS_00019265 TCONS_00078311 TCONS_00051138 TCONS_00033288

TCONS_00091295 TCONS_00053556 TCONS_00054465 TCONS_00081617 TCONS_00081123 TCONS_00025895 TCONS_00082399 TCONS_00028748 TCONS_00082340

TCONS_00081829 TCONS_00068495 TCONS_00002137 TCONS_00004112 TCONS_00007027 TCONS_00078665 TCONS_00084957 TCONS_00025569 TCONS_00079175

TCONS_00018433 TCONS_00019654 TCONS_00079156 TCONS_00033835 TCONS_00086947 TCONS_00002496 TCONS_00033996 TCONS_00049211 TCONS_00090166

TCONS_00065669 TCONS_00079328 TCONS_00027425 TCONS_00047138 TCONS_00081618 TCONS_00022144 TCONS_00064278 TCONS_00064279 TCONS_00021524

TCONS_00068407 TCONS_00087748 TCONS_00011026 TCONS_00024870 TCONS_00071210 TCONS_00003604 TCONS_00033759 TCONS_00085034 TCONS_00019104

TCONS_00067438 TCONS_00068496 TCONS_00061713 TCONS_00078183 TCONS_00056323 TCONS_00056322 TCONS_00067558 TCONS_00077959 TCONS_00018661

TCONS_00089813 TCONS_00077677 TCONS_00022873 TCONS_00042678 TCONS_00079577 TCONS_00091994 TCONS_00030666 TCONS_00090554 TCONS_00086663

TCONS_00087776 TCONS_00022173 TCONS_00001126 TCONS_00086770 TCONS_00067439 TCONS_00007274 TCONS_00067730 TCONS_00023993 TCONS_00039734

TCONS_00076452 TCONS_00060897 TCONS_00003259 TCONS_00064612 TCONS_00089052 TCONS_00090683 TCONS_00080178 TCONS_00018434 TCONS_00019656

TCONS_00090112 TCONS_00054187 TCONS_00056164 TCONS_00053690 TCONS_00054593 TCONS_00067526 TCONS_00036243 TCONS_00024529 TCONS_00057259

TCONS_00018243 TCONS_00019655 TCONS_00027566 TCONS_00040479 TCONS_00080053 TCONS_00035042 TCONS_00056412 TCONS_00067355 TCONS_00088560

TCONS_00033930 TCONS_00033287 TCONS_00056163 TCONS_00080400 TCONS_00080401 TCONS_00034122 TCONS_00057258 TCONS_00053197 TCONS_00011024

TCONS_00064727 TCONS_00016094 TCONS_00078184 TCONS_00088644 TCONS_00003329 TCONS_00002211 TCONS_00028738 TCONS_00080793 TCONS_00057641

TCONS_00010951 TCONS_00050860 TCONS_00089814 TCONS_00065476 TCONS_00040395 TCONS_00090629 TCONS_00064008 TCONS_00002462 TCONS_00040217

TCONS_00003330 TCONS_00056162 TCONS_00042721 TCONS_00042722 TCONS_00037159 TCONS_00003562 TCONS_00081229 TCONS_00049210 TCONS_00053842

TCONS_00088387 TCONS_00036497 TCONS_00036498 TCONS_00082647 TCONS_00014857 TCONS_00002340 TCONS_00002341 TCONS_00040001 TCONS_00040804

TCONS_00011747 TCONS_00011746 TCONS_00057637 TCONS_00010489 TCONS_00007357 TCONS_00014855 TCONS_00014856 TCONS_00064545 TCONS_00014943

TCONS_00056619 TCONS_00030665 TCONS_00034508 TCONS_00043246 TCONS_00012801 TCONS_00005279 TCONS_00018573 TCONS_00002691 TCONS_00052805

TCONS_00060865 TCONS_00084810 TCONS_00043247 TCONS_00015987 TCONS_00017970 TCONS_00007923 TCONS_00080591 TCONS_00019262 TCONS_00079172

TCONS_00070198 TCONS_00033027 TCONS_00044043 TCONS_00034264 TCONS_00004135 TCONS_00009039 TCONS_00089145 TCONS_00057638 TCONS_00025180

TCONS_00036815 TCONS_00037055 TCONS_00080639 TCONS_00090281 TCONS_00077857 TCONS_00033066 TCONS_00083406 TCONS_00056623 TCONS_00046303

TCONS_00044088 TCONS_00053628 TCONS_00012339 TCONS_00002166 TCONS_00007843 TCONS_00024318 TCONS_00033303 TCONS_00088084 TCONS_00012514

TCONS_00049023 TCONS_00057528 TCONS_00079723 TCONS_00037056 TCONS_00062734 TCONS_00028071 TCONS_00049439 TCONS_00038620 TCONS_00069401

TCONS_00003561 TCONS_00046178 TCONS_00036375 TCONS_00037057 TCONS_00081143 TCONS_00002342 TCONS_00042208 TCONS_00043864 TCONS_00003672

TCONS_00042523 TCONS_00043863 TCONS_00007521 TCONS_00042524 TCONS_00014944 TCONS_00054357 TCONS_00000381 TCONS_00049452 TCONS_00004293

TCONS_00046339 TCONS_00052970 TCONS_00081142 TCONS_00057640 TCONS_00056621 TCONS_00056483 TCONS_00065508 TCONS_00044087 TCONS_00030032

TCONS_00040583 TCONS_00078162 TCONS_00040582 TCONS_00027666 TCONS_00046340 TCONS_00056620 TCONS_00083990 TCONS_00028867 TCONS_00050859

TCONS_00057639 TCONS_00002629 TCONS_00028524 TCONS_00083614 TCONS_00036209 TCONS_00064788 TCONS_00080096 TCONS_00086192 TCONS_00088846

TCONS_00082686 TCONS_00069402 TCONS_00080582 TCONS_00024397 TCONS_00040230 TCONS_00083064 TCONS_00008545 TCONS_00045448 TCONS_00007181

TCONS_00062509 TCONS_00055479 TCONS_00007271 TCONS_00001982 TCONS_00019960 TCONS_00079546 TCONS_00022191 TCONS_00030757 TCONS_00064339

TCONS_00049451 TCONS_00078161 TCONS_00018276 TCONS_00078348 TCONS_00014915 TCONS_00090631 TCONS_00089106 TCONS_00087069 TCONS_00074246

TCONS_00003260 TCONS_00080953 TCONS_00022192 TCONS_00052564 TCONS_00024812 TCONS_00008665 TCONS_00007404 TCONS_00058584 TCONS_00019543

TCONS_00084660 TCONS_00024319 TCONS_00037613 TCONS_00036828 TCONS_00071679 TCONS_00081091 TCONS_00004957 TCONS_00090383 TCONS_00055474

TCONS_00015281 TCONS_00028070 TCONS_00008211 TCONS_00007180 TCONS_00016368 TCONS_00022716 TCONS_00024336 TCONS_00082548 TCONS_00080638

TCONS_00019542 TCONS_00078708 TCONS_00011589 TCONS_00025175 TCONS_00004956 TCONS_00061993 TCONS_00044889 TCONS_00061992 TCONS_00031693

TCONS_00007744 TCONS_00084647 TCONS_00045407 TCONS_00078298 TCONS_00023066 TCONS_00004784 TCONS_00011384 TCONS_00014760 TCONS_00024332

TCONS_00006432 TCONS_00052960 TCONS_00059948 TCONS_00003463 TCONS_00008329 TCONS_00083157 TCONS_00008761 TCONS_00080463 TCONS_00061991

TCONS_00071114 TCONS_00018669 TCONS_00004455 TCONS_00002190 TCONS_00053412 TCONS_00053413 TCONS_00005920 TCONS_00007794 TCONS_00083950

TCONS_00084972 TCONS_00089113 TCONS_00067779 TCONS_00077508 TCONS_00067780 TCONS_00024813 TCONS_00028949 TCONS_00085518 TCONS_00033967

TCONS_00021752 TCONS_00024586 TCONS_00045125 TCONS_00005321 TCONS_00058961 TCONS_00011588 TCONS_00014381 TCONS_00015280 TCONS_00025594

TCONS_00053705 TCONS_00090630 TCONS_00054598 TCONS_00016367 TCONS_00024331 TCONS_00050591 TCONS_00084694 TCONS_00027581 TCONS_00007235

TCONS_00035156 TCONS_00046341 TCONS_00083750 TCONS_00091413 TCONS_00033820 TCONS_00080570 TCONS_00082644 TCONS_00034075 TCONS_00067413

TCONS_00087832 TCONS_00002126 TCONS_00052961 TCONS_00033381 TCONS_00064048 TCONS_00081102 TCONS_00027580 TCONS_00086403 TCONS_00020184

TCONS_00042017 TCONS_00046141 TCONS_00002630 TCONS_00054342 TCONS_00014511 TCONS_00073698 TCONS_00028437 TCONS_00013166 TCONS_00024585

TCONS_00040394 TCONS_00077532 TCONS_00065533 TCONS_00083050 TCONS_00086424 TCONS_00003593 TCONS_00091830 TCONS_00084357 TCONS_00053627

TCONS_00053388 TCONS_00090360 TCONS_00036725 TCONS_00022308 TCONS_00037354 TCONS_00023046 TCONS_00027896 TCONS_00028719 TCONS_00060898

TCONS_00030263 TCONS_00091791 TCONS_00007806 TCONS_00025369 TCONS_00077816 TCONS_00064393 TCONS_00002809 TCONS_00007104 TCONS_00019060

TCONS_00021503 TCONS_00086769 TCONS_00049925 TCONS_00065367 TCONS_00044946 TCONS_00043865 TCONS_00079601 TCONS_00010354 TCONS_00053653

TCONS_00064613 TCONS_00083049 TCONS_00042786 TCONS_00064136 TCONS_00081463 TCONS_00019061 TCONS_00042196 TCONS_00090355 TCONS_00016294

TCONS_00082055 TCONS_00079530 TCONS_00024913 TCONS_00007844 TCONS_00067412 TCONS_00013095 TCONS_00025370 TCONS_00056482 TCONS_00082322

TCONS_00084518 TCONS_00056132 TCONS_00056133 TCONS_00004105 TCONS_00090845 TCONS_00090846 TCONS_00046257 TCONS_00008815 TCONS_00080794

TCONS_00023994 TCONS_00007272 TCONS_00083372 TCONS_00007411 TCONS_00015203 TCONS_00007030 TCONS_00004611 TCONS_00034583 TCONS_00064340

TCONS_00065307 TCONS_00077858 TCONS_00079602 TCONS_00044042 TCONS_00080366 TCONS_00064003 TCONS_00046309 TCONS_00043802 TCONS_00081039

TCONS_00036692 TCONS_00014592 TCONS_00019094 TCONS_00027829 TCONS_00048684 TCONS_00046256 TCONS_00002160 TCONS_00008922 TCONS_00078108

TCONS_00003390 TCONS_00026230 TCONS_00037092 TCONS_00004488 TCONS_00033515 TCONS_00024561 TCONS_00054024 TCONS_00081117 TCONS_00067752

TCONS_00045955 TCONS_00080051 TCONS_00018836 TCONS_00039816 TCONS_00011408 TCONS_00008546 TCONS_00005921 TCONS_00092067 TCONS_00026505

TCONS_00028473 TCONS_00024400 TCONS_00004483 TCONS_00078299 TCONS_00036035 TCONS_00086279 TCONS_00049481 TCONS_00050906 TCONS_00039817

TCONS_00018239 TCONS_00036392 TCONS_00051246 TCONS_00080508 TCONS_00054023 TCONS_00009444 TCONS_00081321 TCONS_00020062 TCONS_00056700

TCONS_00010674 TCONS_00002805 TCONS_00055475 TCONS_00061061 TCONS_00007538 TCONS_00046974 TCONS_00037036 TCONS_00008777 TCONS_00057268

TCONS_00057349 TCONS_00072132 TCONS_00080232 TCONS_00024872 TCONS_00039820 TCONS_00025241 TCONS_00046975 TCONS_00002756 TCONS_00024871

TCONS_00040128 TCONS_00023642 TCONS_00059550 TCONS_00083470 TCONS_00078109 TCONS_00014854 TCONS_00081711 TCONS_00034302 TCONS_00087366

TCONS_00062777 TCONS_00067414 TCONS_00082786 TCONS_00021991 TCONS_00022898 TCONS_00072893 TCONS_00061375 TCONS_00021502 TCONS_00019286

TCONS_00004042 TCONS_00028072 TCONS_00008080 TCONS_00012164 TCONS_00059021 TCONS_00015911 TCONS_00041845 TCONS_00084995 TCONS_00012472

TCONS_00018244 TCONS_00056287 TCONS_00064500 TCONS_00024901 TCONS_00040281 TCONS_00089152 TCONS_00061060 TCONS_00022893 TCONS_00088647

TCONS_00040201 TCONS_00079568 TCONS_00007729 TCONS_00079218 TCONS_00088648 TCONS_00021793 TCONS_00075107 TCONS_00084359 TCONS_00004128

TCONS_00045820 TCONS_00023067 TCONS_00082500 TCONS_00018958 TCONS_00034664 TCONS_00084358 TCONS_00041843 TCONS_00043800 TCONS_00068599

TCONS_00010902 TCONS_00061522 TCONS_00056301 TCONS_00091581 TCONS_00033514 TCONS_00058715 TCONS_00067553 TCONS_00034665 TCONS_00034666

TCONS_00055192 TCONS_00012542 TCONS_00025596 TCONS_00009160 TCONS_00077401 TCONS_00024652 TCONS_00068600 TCONS_00066006 TCONS_00086732

TCONS_00011285 TCONS_00079589 TCONS_00037847 TCONS_00007728 TCONS_00081457 TCONS_00090916 TCONS_00048824 TCONS_00004501 TCONS_00004502

TCONS_00013962 TCONS_00048825 TCONS_00002026 TCONS_00028262 TCONS_00067554 TCONS_00030640 TCONS_00031586 TCONS_00031587 TCONS_00077914

TCONS_00068601 TCONS_00034694 TCONS_00014466 TCONS_00086731 TCONS_00045393 TCONS_00061100 TCONS_00091373 TCONS_00056300 TCONS_00031694

TCONS_00084375 TCONS_00077915 TCONS_00028679 TCONS_00027855 TCONS_00078518 TCONS_00030639 TCONS_00061498 TCONS_00084074 TCONS_00002676

TCONS_00081546 TCONS_00012261 TCONS_00079598 TCONS_00002690 TCONS_00080816 TCONS_00060285 TCONS_00046062 TCONS_00075106 TCONS_00003611

TCONS_00036391 TCONS_00061352 TCONS_00054105 TCONS_00061453 TCONS_00090867 TCONS_00011023 TCONS_00061452 TCONS_00066534 TCONS_00015780

TCONS_00042252 TCONS_00081216 TCONS_00081217 TCONS_00061454 TCONS_00061165 TCONS_00065562 TCONS_00090077 TCONS_00083030 TCONS_00083031

TCONS_00064544 TCONS_00040280 TCONS_00061733 TCONS_00008082 TCONS_00062074 TCONS_00026381 TCONS_00022872 TCONS_00002699 TCONS_00088748

TCONS_00069255 TCONS_00082757 TCONS_00015264 TCONS_00072277 TCONS_00050710 TCONS_00068266 TCONS_00015263 TCONS_00057000 TCONS_00082301

TCONS_00033823 TCONS_00081710 TCONS_00018194 TCONS_00061757 TCONS_00082933 TCONS_00025468 TCONS_00007805 TCONS_00088046 TCONS_00052066

TCONS_00018193 TCONS_00083032 TCONS_00090617 TCONS_00079693 TCONS_00009819 TCONS_00056182 TCONS_00026396 TCONS_00081458 TCONS_00055855

TCONS_00055856 TCONS_00057020 TCONS_00057392 TCONS_00057393 TCONS_00092573 TCONS_00042249 TCONS_00033734 TCONS_00034194 TCONS_00018195

TCONS_00009820 TCONS_00021794 TCONS_00070180 TCONS_00080376 TCONS_00008081 TCONS_00082984 TCONS_00061351 TCONS_00049200 TCONS_00049201

TCONS_00039990 TCONS_00062423 TCONS_00033822 TCONS_00064672 TCONS_00042020 TCONS_00040202 TCONS_00011523 TCONS_00001980 TCONS_00054104

TCONS_00060968 TCONS_00078960 TCONS_00025155 TCONS_00012642 TCONS_00052566 TCONS_00089050 TCONS_00014707 TCONS_00031276 TCONS_00084127

TCONS_00008046 TCONS_00024781 TCONS_00021526 TCONS_00008760 TCONS_00049621 TCONS_00053629 TCONS_00030163 TCONS_00026061 TCONS_00075217

TCONS_00004603 TCONS_00068757 TCONS_00000988 TCONS_00067301 TCONS_00008045 TCONS_00060969 TCONS_00056324 TCONS_00026503 TCONS_00062084

TCONS_00078961 TCONS_00048927 TCONS_00034698 TCONS_00021258 TCONS_00033542 TCONS_00012259 TCONS_00062251 TCONS_00055191 TCONS_00012260

TCONS_00089047 TCONS_00028474 TCONS_00041373 TCONS_00077890 TCONS_00079224 TCONS_00051963 TCONS_00026380 TCONS_00030781 TCONS_00002489

TCONS_00004367 TCONS_00089046 TCONS_00026237 TCONS_00022216 TCONS_00087616 TCONS_00064294 TCONS_00013593 TCONS_00014897 TCONS_00024889

TCONS_00084811 TCONS_00051964 TCONS_00067506 TCONS_00086913 TCONS_00067507 TCONS_00074456 TCONS_00082756 TCONS_00031715 TCONS_00077584

TCONS_00086452 TCONS_00003997 TCONS_00066489 TCONS_00026379 TCONS_00064193 TCONS_00023083 TCONS_00002158 TCONS_00004692 TCONS_00014593

TCONS_00015245 TCONS_00061156 TCONS_00025907 TCONS_00024900 TCONS_00002497 TCONS_00064310 TCONS_00083767 TCONS_00042198 TCONS_00022294

TCONS_00068756 TCONS_00020097 TCONS_00023995 TCONS_00061557 TCONS_00020546 TCONS_00083135 TCONS_00072131 TCONS_00057478 TCONS_00051292

TCONS_00011166 TCONS_00064791 TCONS_00035128 TCONS_00061435 TCONS_00064792 TCONS_00081044 TCONS_00080154 TCONS_00003675 TCONS_00023101

TCONS_00083134 TCONS_00021602 TCONS_00042288 TCONS_00049141 TCONS_00080287 TCONS_00045162 TCONS_00064309 TCONS_00074513 TCONS_00053066

TCONS_00074514 TCONS_00091579 TCONS_00042287 TCONS_00050922 TCONS_00087102 TCONS_00035036 TCONS_00049441 TCONS_00040126 TCONS_00042130

TCONS_00018238 TCONS_00083560 TCONS_00091984 TCONS_00084693 TCONS_00090615 TCONS_00062249 TCONS_00062250 TCONS_00084946 TCONS_00024890

TCONS_00068095 TCONS_00079532 TCONS_00049415 TCONS_00011546 TCONS_00040127 TCONS_00061903 TCONS_00062494 TCONS_00088184 TCONS_00052565

TCONS_00040219 TCONS_00048020 TCONS_00069467 TCONS_00085023 TCONS_00087405 TCONS_00087406 TCONS_00079595 TCONS_00018675 TCONS_00007275

TCONS_00028042 TCONS_00068603 TCONS_00079594 TCONS_00007724 TCONS_00020023 TCONS_00061436 TCONS_00082327 TCONS_00030241 TCONS_00068498

TCONS_00044766 TCONS_00016329 TCONS_00067557 TCONS_00087987 TCONS_00011021 TCONS_00079771 TCONS_00007276 TCONS_00018676 TCONS_00028663

TCONS_00079533 TCONS_00011022 TCONS_00068755 TCONS_00087215 TCONS_00011631 TCONS_00043191 TCONS_00037604 TCONS_00049457 TCONS_00030242

TCONS_00036128 TCONS_00078236 TCONS_00025615 TCONS_00067348 TCONS_00082265 TCONS_00078511 TCONS_00065667 TCONS_00087988 TCONS_00061205

TCONS_00012660 TCONS_00018524 TCONS_00088037 TCONS_00005922 TCONS_00049768 TCONS_00026060 TCONS_00066001 TCONS_00059735 TCONS_00039988

TCONS_00090772 TCONS_00086660 TCONS_00067031 TCONS_00067029 TCONS_00067030 TCONS_00068094 TCONS_00068093 TCONS_00062722 TCONS_00062723

TCONS_00011250 TCONS_00039770 TCONS_00068092 TCONS_00019822 TCONS_00080079 TCONS_00008979 TCONS_00004085 TCONS_00064392 TCONS_00031224

TCONS_00031225 TCONS_00055836 TCONS_00088115 TCONS_00071250 TCONS_00024563 TCONS_00086661 TCONS_00030514 TCONS_00019614 TCONS_00085019

TCONS_00030860 TCONS_00041098 TCONS_00020176 TCONS_00015609 TCONS_00044884 TCONS_00060888 TCONS_00060889 TCONS_00067032 TCONS_00039130

TCONS_00080017 TCONS_00081807 TCONS_00089679 TCONS_00003385 TCONS_00003386 TCONS_00041802 TCONS_00024915 TCONS_00008978 TCONS_00018085

TCONS_00030861 TCONS_00081808 TCONS_00022538 TCONS_00022539 TCONS_00090775 TCONS_00004484 TCONS_00065238 TCONS_00012444 TCONS_00010257

TCONS_00011393 TCONS_00014362 TCONS_00049897 TCONS_00082760 TCONS_00084599 TCONS_00064225 TCONS_00091730 TCONS_00033034 TCONS_00049318

TCONS_00084380 TCONS_00068483 TCONS_00079905 TCONS_00091104 TCONS_00037848 TCONS_00078959 TCONS_00051041 TCONS_00087615 TCONS_00052503

TCONS_00019374 TCONS_00011394 TCONS_00088704 TCONS_00085020 TCONS_00006535 TCONS_00049317 TCONS_00078621 TCONS_00087295 TCONS_00083597

TCONS_00044916 TCONS_00019911 TCONS_00077913 TCONS_00018780 TCONS_00019912 TCONS_00062105 TCONS_00027570 TCONS_00087871 TCONS_00044787

TCONS_00079915 TCONS_00070621 TCONS_00022896 TCONS_00060991 TCONS_00088442 TCONS_00024570 TCONS_00061716 TCONS_00061717 TCONS_00022995

TCONS_00082309 TCONS_00077784 TCONS_00032696 TCONS_00090087 TCONS_00046915 TCONS_00084434 TCONS_00067310 TCONS_00038072 TCONS_00039821

TCONS_00042368 TCONS_00082167 TCONS_00037954 TCONS_00022968 TCONS_00002729 TCONS_00009259 TCONS_00031659 TCONS_00002677 TCONS_00009188

TCONS_00068372 TCONS_00024610 TCONS_00041404 TCONS_00082561 TCONS_00061166 TCONS_00011112 TCONS_00051269 TCONS_00021012 TCONS_00019375

TCONS_00022994 TCONS_00077783 TCONS_00077782 TCONS_00090291 TCONS_00002125 TCONS_00039848 TCONS_00022724 TCONS_00067309 TCONS_00002124

TCONS_00019867 TCONS_00005356 TCONS_00062076 TCONS_00025386 TCONS_00070622 TCONS_00021489 TCONS_00022599 TCONS_00041801 TCONS_00060447

TCONS_00039769 TCONS_00019866 TCONS_00087883 TCONS_00002932 TCONS_00090844 TCONS_00061712 TCONS_00079949 TCONS_00012555 TCONS_00018240

TCONS_00044799 TCONS_00068360 TCONS_00004189 TCONS_00083507 TCONS_00010900 TCONS_00079914 TCONS_00044384 TCONS_00079811 TCONS_00064080

TCONS_00084170 TCONS_00075223 TCONS_00079496 TCONS_00088042 TCONS_00067753 TCONS_00058937 TCONS_00071041 TCONS_00017497 TCONS_00044904

TCONS_00084699 TCONS_00042903 TCONS_00004711 TCONS_00033884 TCONS_00022111 TCONS_00036932 TCONS_00061362 TCONS_00042983 TCONS_00079810

TCONS_00087218 TCONS_00049732 TCONS_00046718 TCONS_00091796 TCONS_00087217 TCONS_00080664 TCONS_00091795 TCONS_00050849 TCONS_00011286

TCONS_00018908 TCONS_00046973 TCONS_00059672 TCONS_00040692 TCONS_00068497 TCONS_00079534 TCONS_00049234 TCONS_00068317 TCONS_00067442

TCONS_00046798 TCONS_00057242 TCONS_00090288 TCONS_00057241 TCONS_00034239 TCONS_00087219 TCONS_00057425 TCONS_00011542 TCONS_00061743

TCONS_00041800 TCONS_00009020 TCONS_00078185 TCONS_00022112 TCONS_00070171 TCONS_00001555 TCONS_00053132 TCONS_00041109 TCONS_00054406

TCONS_00012630 TCONS_00022895 TCONS_00046867 TCONS_00091462 TCONS_00016778 TCONS_00081283 TCONS_00089253 TCONS_00063964 TCONS_00068499

TCONS_00051271 TCONS_00008740 TCONS_00025156 TCONS_00086948 TCONS_00049235 TCONS_00033925 TCONS_00073984 TCONS_00008415 TCONS_00033988

TCONS_00087742 TCONS_00030165 TCONS_00003673 TCONS_00086287 TCONS_00074458 TCONS_00083709 TCONS_00022293 TCONS_00042904 TCONS_00079387

TCONS_00026375 TCONS_00035530 TCONS_00081284 TCONS_00012367 TCONS_00001253 TCONS_00007247 TCONS_00058803 TCONS_00053091 TCONS_00042016

TCONS_00084845 TCONS_00048929 TCONS_00083596 TCONS_00040691 TCONS_00011165 TCONS_00042615 TCONS_00041403 TCONS_00011222 TCONS_00056638

TCONS_00030164 TCONS_00065329 TCONS_00021429 TCONS_00064347 TCONS_00083207 TCONS_00021102 TCONS_00043085 TCONS_00015619 TCONS_00082325

TCONS_00074256 TCONS_00015178 TCONS_00083508 TCONS_00041364 TCONS_00064807 TCONS_00077912 TCONS_00046799 TCONS_00008664 TCONS_00084407

TCONS_00074848 TCONS_00044915 TCONS_00083853 TCONS_00083266 TCONS_00090771 TCONS_00082755 TCONS_00077512 TCONS_00083854 TCONS_00074650

TCONS_00065330 TCONS_00004489 TCONS_00053032 TCONS_00019287 TCONS_00054078 TCONS_00053486 TCONS_00065014 TCONS_00067441 TCONS_00003612

TCONS_00018994 TCONS_00088441 TCONS_00054561 TCONS_00053650 TCONS_00022113 TCONS_00091488 TCONS_00065694 TCONS_00056468 TCONS_00053000

TCONS_00012325 TCONS_00020076 TCONS_00050783 TCONS_00017574 TCONS_00018861 TCONS_00070620 TCONS_00020092 TCONS_00020093 TCONS_00049769

TCONS_00044903 TCONS_00079535 TCONS_00004421 TCONS_00011284 TCONS_00012471 TCONS_00079388 TCONS_00045423 TCONS_00041160 TCONS_00088701

TCONS_00043111 TCONS_00030218 TCONS_00031264 TCONS_00041237 TCONS_00064488 TCONS_00065629 TCONS_00046825 TCONS_00084119 TCONS_00067060

TCONS_00091684 TCONS_00057244 TCONS_00042132 TCONS_00061350 TCONS_00078110 TCONS_00057516 TCONS_00067440 TCONS_00079310 TCONS_00091018

TCONS_00091019 TCONS_00091020 TCONS_00087210 TCONS_00034016 TCONS_00035068 TCONS_00078623 TCONS_00046096 TCONS_00005346 TCONS_00007502

TCONS_00078249 TCONS_00035069 TCONS_00092044 TCONS_00049357 TCONS_00035216 TCONS_00091367 TCONS_00080617 TCONS_00078515 TCONS_00008377

TCONS_00056156 TCONS_00056157 TCONS_00065270 TCONS_00041238 TCONS_00067027 TCONS_00018320 TCONS_00013183 TCONS_00076539 TCONS_00018321

TCONS_00067479 TCONS_00057243 TCONS_00057245 TCONS_00015873 TCONS_00019990 TCONS_00007921 TCONS_00009084 TCONS_00056021 TCONS_00063999

TCONS_00079373 TCONS_00017591 TCONS_00002912 TCONS_00014553 TCONS_00067028 TCONS_00010823 TCONS_00031519 TCONS_00068226 TCONS_00065999

TCONS_00086379 TCONS_00003608 TCONS_00043086 TCONS_00043087 TCONS_00053651 TCONS_00070179 TCONS_00004298 TCONS_00065052 TCONS_00065269

TCONS_00022511 TCONS_00065051 TCONS_00079386 TCONS_00081712 TCONS_00050784 TCONS_00014552 TCONS_00037014 TCONS_00060961 TCONS_00002627

TCONS_00078517 TCONS_00030273 TCONS_00081714 TCONS_00018820 TCONS_00041724 TCONS_00081713 TCONS_00078516 TCONS_00024282 TCONS_00044143

TCONS_00063998 TCONS_00067349 TCONS_00054013 TCONS_00030535 TCONS_00030536 TCONS_00060960 TCONS_00062071 TCONS_00062072 TCONS_00050811

TCONS_00091683 TCONS_00089608 TCONS_00046059 TCONS_00004530 TCONS_00004531 TCONS_00004532 TCONS_00088626 TCONS_00050779 TCONS_00056470

TCONS_00019939 TCONS_00063997 TCONS_00064262 TCONS_00078705 TCONS_00004529 TCONS_00017519 TCONS_00048771 TCONS_00083598 TCONS_00073314

TCONS_00064202 TCONS_00039718 TCONS_00085126 TCONS_00073313 TCONS_00089607 TCONS_00067151 TCONS_00059795 TCONS_00081589 TCONS_00011490

TCONS_00055858 TCONS_00040624 TCONS_00046095 TCONS_00059796 TCONS_00026853 TCONS_00064203 TCONS_00041487 TCONS_00041488 TCONS_00015752

TCONS_00033250 TCONS_00062495 TCONS_00041489 TCONS_00086483 TCONS_00090584 TCONS_00058894 TCONS_00058337 TCONS_00043288 TCONS_00043289

TCONS_00016444 TCONS_00064837 TCONS_00043290 TCONS_00041905 TCONS_00018377 TCONS_00046179 TCONS_00053014 TCONS_00091366 TCONS_00062173

TCONS_00015409 TCONS_00015751 TCONS_00058545 TCONS_00061339 TCONS_00087129 TCONS_00050648 TCONS_00007300 TCONS_00007301 TCONS_00077471

TCONS_00008567 TCONS_00008568 TCONS_00055955 TCONS_00088588 TCONS_00039520 TCONS_00039368 TCONS_00011742 TCONS_00015408 TCONS_00062174

TCONS_00087209 TCONS_00027689 TCONS_00045266 TCONS_00019514 TCONS_00044014 TCONS_00046479 TCONS_00090688 TCONS_00067421 TCONS_00077359

TCONS_00083852 TCONS_00042556 TCONS_00034006 TCONS_00011744 TCONS_00041273 TCONS_00083343 TCONS_00054832 TCONS_00050649 TCONS_00080373

TCONS_00088587 TCONS_00056835 TCONS_00056279 TCONS_00069007 TCONS_00003346 TCONS_00014708 TCONS_00015779 TCONS_00056257 TCONS_00083149

TCONS_00002794 TCONS_00080532 TCONS_00077472 TCONS_00040259 TCONS_00040258 TCONS_00056048 TCONS_00011743 TCONS_00041517 TCONS_00001372

TCONS_00008523 TCONS_00040327 TCONS_00057022 TCONS_00089414 TCONS_00069006 TCONS_00057517 TCONS_00083707 TCONS_00057153 TCONS_00012820

TCONS_00057152 TCONS_00059677 TCONS_00022255 TCONS_00014798 TCONS_00077865 TCONS_00039279 TCONS_00049138 TCONS_00036826 TCONS_00018659

TCONS_00045598 TCONS_00009090 TCONS_00012819 TCONS_00079639 TCONS_00038095 TCONS_00061063 TCONS_00080118 TCONS_00043026 TCONS_00021719

TCONS_00041374 TCONS_00087364 TCONS_00068399 TCONS_00083269 TCONS_00012818 TCONS_00003345 TCONS_00033826 TCONS_00090232 TCONS_00022003

TCONS_00013045 TCONS_00056320 TCONS_00049124 TCONS_00013046 TCONS_00080530 TCONS_00080531 TCONS_00067373 TCONS_00045155 TCONS_00077495

TCONS_00009091 TCONS_00079229 TCONS_00061594 TCONS_00045005 TCONS_00037319 TCONS_00003119 TCONS_00004594 TCONS_00030525 TCONS_00067680

TCONS_00005027 TCONS_00040701 TCONS_00020680 TCONS_00078156 TCONS_00005026 TCONS_00005028 TCONS_00055567 TCONS_00024897 TCONS_00035217

TCONS_00086392 TCONS_00007248 TCONS_00057072 TCONS_00062641 TCONS_00040907 TCONS_00040908 TCONS_00005374 TCONS_00082262 TCONS_00088018

TCONS_00040700 TCONS_00061592 TCONS_00091200 TCONS_00088130 TCONS_00052774 TCONS_00024793 TCONS_00014428 TCONS_00061900 TCONS_00061902

TCONS_00061593 TCONS_00040381 TCONS_00049767 TCONS_00039902 TCONS_00017306 TCONS_00043192 TCONS_00034240 TCONS_00030099 TCONS_00040197

TCONS_00050273 TCONS_00008566 TCONS_00012694 TCONS_00015659 TCONS_00015087 TCONS_00003343 TCONS_00080074 TCONS_00075327 TCONS_00042352

TCONS_00003344 TCONS_00083934 TCONS_00003342 TCONS_00083609 TCONS_00013960 TCONS_00045708 TCONS_00036675 TCONS_00040672 TCONS_00080778

TCONS_00021527 TCONS_00077422 TCONS_00089415 TCONS_00024725 TCONS_00061689 TCONS_00049863 TCONS_00079228 TCONS_00012632 TCONS_00064206

TCONS_00004020 TCONS_00030098 TCONS_00064207 TCONS_00014386 TCONS_00023118 TCONS_00061901 TCONS_00049378 TCONS_00001998 TCONS_00045025

TCONS_00061286 TCONS_00090096 TCONS_00082263 TCONS_00079628 TCONS_00088071 TCONS_00042220 TCONS_00006546 TCONS_00071598 TCONS_00084974

TCONS_00078044 TCONS_00090060 TCONS_00031154 TCONS_00042219 TCONS_00045024 TCONS_00041365 TCONS_00091253 TCONS_00064838 TCONS_00060583

TCONS_00050822 TCONS_00025160 TCONS_00084381 TCONS_00088649 TCONS_00062124 TCONS_00055951 TCONS_00021731 TCONS_00024789 TCONS_00020695

TCONS_00067939 TCONS_00061287 TCONS_00046506 TCONS_00088533 TCONS_00005025 TCONS_00058959 TCONS_00082558 TCONS_00051881 TCONS_00067990

TCONS_00084629 TCONS_00072836 TCONS_00084825 TCONS_00047396 TCONS_00008067 TCONS_00061013 TCONS_00007299 TCONS_00079714 TCONS_00061288

TCONS_00063804 TCONS_00059454 TCONS_00077358 TCONS_00066932 TCONS_00079521 TCONS_00003738 TCONS_00005372 TCONS_00005373 TCONS_00059495

TCONS_00039826 TCONS_00047395 TCONS_00056834 TCONS_00081450 TCONS_00078367 TCONS_00056833 TCONS_00083608 TCONS_00056832 TCONS_00059205

TCONS_00059206 TCONS_00080300 TCONS_00057649 TCONS_00057650 TCONS_00090949 TCONS_00007050 TCONS_00043216 TCONS_00081833 TCONS_00002673

TCONS_00045023 TCONS_00058728 TCONS_00089389 TCONS_00042628 TCONS_00087083 TCONS_00003341 TCONS_00022292 TCONS_00011545 TCONS_00022530

TCONS_00041161 TCONS_00011493 TCONS_00036127 TCONS_00041254 TCONS_00024724 TCONS_00031810 TCONS_00054251 TCONS_00079464 TCONS_00001188

TCONS_00051880 TCONS_00058621 TCONS_00002985 TCONS_00090430 TCONS_00088278 TCONS_00090431 TCONS_00082179 TCONS_00004481 TCONS_00089699

TCONS_00015222 TCONS_00082028 TCONS_00071072 TCONS_00076474 TCONS_00030100 TCONS_00016189 TCONS_00016317 TCONS_00002493 TCONS_00011492

TCONS_00021979 TCONS_00044909 TCONS_00040690 TCONS_00010899 TCONS_00007625 TCONS_00008066 TCONS_00054441 TCONS_00015086 TCONS_00041402

TCONS_00009177 TCONS_00022894 TCONS_00055857 TCONS_00031730 TCONS_00084824 TCONS_00091878 TCONS_00025500 TCONS_00042660 TCONS_00008065

TCONS_00052379 TCONS_00082359 TCONS_00090097 TCONS_00025460 TCONS_00057221 TCONS_00064878 TCONS_00065729 TCONS_00089700 TCONS_00046396

TCONS_00007941 TCONS_00087365 TCONS_00057222 TCONS_00082323 TCONS_00063373 TCONS_00031520 TCONS_00024770 TCONS_00035122 TCONS_00025159

TCONS_00051346 TCONS_00054355 TCONS_00079972 TCONS_00083837 TCONS_00063374 TCONS_00012415 TCONS_00042199 TCONS_00072478 TCONS_00023028

TCONS_00081847 TCONS_00053854 TCONS_00054118 TCONS_00056363 TCONS_00067026 TCONS_00068091 TCONS_00030537 TCONS_00023686 TCONS_00080230

TCONS_00024572 TCONS_00022126 TCONS_00031275 TCONS_00033593 TCONS_00056364 TCONS_00056365 TCONS_00057424 TCONS_00022115 TCONS_00050780

TCONS_00043259 TCONS_00003220 TCONS_00024746 TCONS_00023001 TCONS_00011213 TCONS_00078414 TCONS_00043258 TCONS_00034727 TCONS_00057021

TCONS_00042039 TCONS_00075744 TCONS_00018603 TCONS_00081927 TCONS_00040017 TCONS_00069448 TCONS_00043257 TCONS_00042040 TCONS_00040015

TCONS_00040018 TCONS_00040016 TCONS_00089784 TCONS_00089785 TCONS_00060286 TCONS_00082101 TCONS_00053245 TCONS_00042041 TCONS_00075088

TCONS_00025470 TCONS_00004369 TCONS_00068158 TCONS_00057344 TCONS_00015085 TCONS_00013835 TCONS_00082761 TCONS_00090101 TCONS_00002674

TCONS_00009983 TCONS_00090100 TCONS_00033594 TCONS_00007868 TCONS_00058623 TCONS_00023933 TCONS_00007869 TCONS_00081780 TCONS_00009045

TCONS_00016207 TCONS_00040572 TCONS_00049199 TCONS_00050696 TCONS_00056278 TCONS_00080991 TCONS_00006561 TCONS_00037786 TCONS_00009046

TCONS_00037793 TCONS_00022148 TCONS_00025529 TCONS_00000508 TCONS_00026543 TCONS_00059132 TCONS_00037593 TCONS_00053010 TCONS_00023685

TCONS_00015023 TCONS_00091822 TCONS_00040673 TCONS_00083708 TCONS_00022149 TCONS_00041459 TCONS_00041458 TCONS_00022609 TCONS_00075347

TCONS_00081090 TCONS_00078519 TCONS_00034740 TCONS_00021915 TCONS_00020862 TCONS_00053446 TCONS_00089504 TCONS_00088836 TCONS_00015235

TCONS_00070795 TCONS_00046209 TCONS_00079843 TCONS_00082324 TCONS_00042671 TCONS_00070182 TCONS_00030794 TCONS_00036872 TCONS_00068140

TCONS_00011369 TCONS_00015411 TCONS_00062405 TCONS_00090127 TCONS_00019428 TCONS_00010009 TCONS_00061570 TCONS_00046210 TCONS_00011242

TCONS_00089357 TCONS_00090173 TCONS_00003198 TCONS_00078349 TCONS_00015056 TCONS_00084740 TCONS_00050853 TCONS_00023745 TCONS_00024627

TCONS_00041409 TCONS_00065313 TCONS_00070183 TCONS_00024792 TCONS_00047772 TCONS_00053354 TCONS_00090322 TCONS_00082758 TCONS_00079037

TCONS_00000647 TCONS_00014921 TCONS_00082531 TCONS_00033473 TCONS_00072484 TCONS_00018258 TCONS_00031308 TCONS_00064000 TCONS_00075526

TCONS_00023672 TCONS_00058537 TCONS_00035457 TCONS_00030573 TCONS_00031307 TCONS_00030244 TCONS_00026088 TCONS_00013393 TCONS_00070806

TCONS_00031277 TCONS_00013595 TCONS_00082964 TCONS_00028830 TCONS_00056825 TCONS_00090289 TCONS_00080980 TCONS_00071179 TCONS_00018257

TCONS_00014934 TCONS_00030759 TCONS_00082829 TCONS_00009842 TCONS_00050852 TCONS_00058622 TCONS_00006975 TCONS_00021413 TCONS_00024769

TCONS_00006360 TCONS_00025343 TCONS_00082963 TCONS_00072657 TCONS_00060176 TCONS_00030715 TCONS_00064302 TCONS_00064652 TCONS_00024550

TCONS_00091188 TCONS_00079038 TCONS_00028598 TCONS_00033827 TCONS_00050698 TCONS_00005320 TCONS_00081800 TCONS_00069271 TCONS_00088279

TCONS_00030272 TCONS_00005319 TCONS_00081928 TCONS_00062612 TCONS_00059364 TCONS_00091823 TCONS_00091824 TCONS_00052374 TCONS_00088633

TCONS_00052380 TCONS_00016477 TCONS_00077483 TCONS_00025949 TCONS_00030271 TCONS_00008866 TCONS_00018140 TCONS_00055539 TCONS_00010896

TCONS_00024820 TCONS_00036685 TCONS_00081801 TCONS_00001199 TCONS_00008906 TCONS_00014427 TCONS_00050697 TCONS_00053015 TCONS_00062502

TCONS_00058439 TCONS_00021614 TCONS_00012209 TCONS_00007442 TCONS_00015081 TCONS_00010226 TCONS_00053119 TCONS_00035458 TCONS_00006993

TCONS_00077842 TCONS_00077813 TCONS_00045383 TCONS_00080981 TCONS_00070662 TCONS_00006359 TCONS_00027553 TCONS_00026124 TCONS_00015725

TCONS_00056439 TCONS_00086675 TCONS_00079245 TCONS_00070844 TCONS_00083791 TCONS_00031547 TCONS_00015784 TCONS_00023064 TCONS_00023065

TCONS_00056362 TCONS_00025501 TCONS_00081929 TCONS_00003219 TCONS_00079276 TCONS_00005631 TCONS_00024768 TCONS_00012162 TCONS_00007198

TCONS_00058353 TCONS_00019521 TCONS_00054374 TCONS_00013264 TCONS_00063224 TCONS_00044865 TCONS_00082635 TCONS_00005060 TCONS_00005061

TCONS_00005062 TCONS_00004806 TCONS_00055292 TCONS_00043214 TCONS_00032847 TCONS_00088717 TCONS_00008469 TCONS_00060580 TCONS_00060579

TCONS_00053441 TCONS_00034831 TCONS_00067102 TCONS_00068188 TCONS_00014608 TCONS_00058244 TCONS_00002551 TCONS_00018141 TCONS_00042828

TCONS_00003061 TCONS_00077841 TCONS_00006216 TCONS_00002864 TCONS_00054381 TCONS_00057329 TCONS_00067777 TCONS_00058245 TCONS_00015455

TCONS_00072477 TCONS_00091879 TCONS_00086380 TCONS_00046507 TCONS_00042891 TCONS_00087602 TCONS_00013807 TCONS_00031546 TCONS_00054383

TCONS_00016058 TCONS_00025406 TCONS_00069228 TCONS_00053504 TCONS_00033987 TCONS_00019940 TCONS_00042182 TCONS_00044866 TCONS_00054422

TCONS_00058908 TCONS_00014591 TCONS_00090287 TCONS_00079043 TCONS_00065217 TCONS_00090728 TCONS_00090729 TCONS_00079236 TCONS_00056508

TCONS_00020004 TCONS_00024694 TCONS_00040243 TCONS_00062595 TCONS_00014978 TCONS_00048034 TCONS_00031402 TCONS_00045857 TCONS_00046508

TCONS_00009675 TCONS_00035106 TCONS_00053355 TCONS_00035105 TCONS_00034077 TCONS_00003348 TCONS_00013174 TCONS_00087363 TCONS_00003347

TCONS_00001200 TCONS_00052504 TCONS_00033828 TCONS_00052244 TCONS_00070600 TCONS_00078943 TCONS_00030933 TCONS_00012282 TCONS_00061540

TCONS_00052309 TCONS_00007592 TCONS_00077474 TCONS_00069496 TCONS_00032845 TCONS_00078695 TCONS_00078576 TCONS_00035235 TCONS_00034262

TCONS_00079042 TCONS_00049077 TCONS_00050584 TCONS_00010769 TCONS_00010770 TCONS_00034663 TCONS_00068716 TCONS_00057932 TCONS_00042596

TCONS_00087107 TCONS_00025387 TCONS_00091187 TCONS_00080472 TCONS_00040674 TCONS_00046914 TCONS_00004470 TCONS_00062549 TCONS_00011643

TCONS_00036129 TCONS_00054338 TCONS_00081912 TCONS_00083246 TCONS_00088658 TCONS_00024638 TCONS_00078942 TCONS_00031114 TCONS_00012751

TCONS_00030932 TCONS_00070245 TCONS_00074850 TCONS_00061755 TCONS_00078389 TCONS_00004503 TCONS_00012032 TCONS_00031686 TCONS_00048979

TCONS_00090286 TCONS_00090926 TCONS_00007889 TCONS_00019924 TCONS_00078154 TCONS_00029049 TCONS_00049230 TCONS_00020979 TCONS_00034263

TCONS_00073544 TCONS_00063372 TCONS_00052258 TCONS_00044336 TCONS_00089588 TCONS_00020592 TCONS_00078153 TCONS_00010156 TCONS_00009166

TCONS_00044337 TCONS_00016857 TCONS_00065055 TCONS_00063277 TCONS_00077475 TCONS_00052199 TCONS_00033554 TCONS_00042458 TCONS_00043215

TCONS_00065469 TCONS_00078415 TCONS_00020967 TCONS_00031249 TCONS_00031865 TCONS_00013828 TCONS_00021638 TCONS_00046599 TCONS_00012163

TCONS_00012161 TCONS_00036619 TCONS_00003410 TCONS_00072887 TCONS_00072202 TCONS_00067053 TCONS_00026436 TCONS_00065342 TCONS_00086992

TCONS_00069805 TCONS_00009168 TCONS_00077473 TCONS_00080378 TCONS_00024560 TCONS_00034728 TCONS_00050510 TCONS_00050511 TCONS_00079360

TCONS_00050719 TCONS_00074849 TCONS_00042665 TCONS_00018888 TCONS_00064001 TCONS_00080995 TCONS_00089670 TCONS_00053929 TCONS_00054318

TCONS_00008053 TCONS_00081422 TCONS_00054317 TCONS_00018889 TCONS_00014389 TCONS_00019097 TCONS_00083054 TCONS_00073554 TCONS_00078689

TCONS_00052373 TCONS_00080554 TCONS_00021732 TCONS_00053944 TCONS_00037745 TCONS_00017614 TCONS_00082099 TCONS_00007227 TCONS_00071841

TCONS_00080370 TCONS_00012543 TCONS_00086393 TCONS_00089311 TCONS_00065244 TCONS_00042180 TCONS_00042181 TCONS_00022690 TCONS_00021169

TCONS_00080470 TCONS_00008052 TCONS_00046347 TCONS_00061754 TCONS_00060820 TCONS_00028459 TCONS_00089008 TCONS_00041390 TCONS_00087818

TCONS_00039989 TCONS_00045089 TCONS_00044561 TCONS_00086880 TCONS_00012692 TCONS_00012693 TCONS_00031118 TCONS_00031685 TCONS_00089416

TCONS_00078636 TCONS_00008807 TCONS_00081674 TCONS_00063278 TCONS_00052375 TCONS_00073275 TCONS_00040843 TCONS_00020179 TCONS_00089356

TCONS_00081916 TCONS_00073553 TCONS_00013601 TCONS_00052896 TCONS_00033343 TCONS_00048144 TCONS_00088430 TCONS_00079273 TCONS_00045926

TCONS_00029230 TCONS_00080330 TCONS_00080377 TCONS_00011610 TCONS_00004463 TCONS_00005305 TCONS_00002644 TCONS_00070842 TCONS_00061285

TCONS_00070841 TCONS_00046287 TCONS_00024617 TCONS_00076291 TCONS_00001333 TCONS_00001334 TCONS_00007890 TCONS_00086894 TCONS_00083114

TCONS_00053011 TCONS_00020696 TCONS_00061002 TCONS_00057779 TCONS_00064034 TCONS_00087318 TCONS_00054861 TCONS_00061003 TCONS_00021883

TCONS_00058797 TCONS_00027644 TCONS_00047542 TCONS_00005178 TCONS_00021622 TCONS_00033506 TCONS_00034653 TCONS_00051311 TCONS_00080211

TCONS_00086468 TCONS_00087959 TCONS_00052377 TCONS_00072030 TCONS_00069270 TCONS_00005611 TCONS_00083359 TCONS_00083360 TCONS_00080475

TCONS_00011176 TCONS_00079677 TCONS_00000501 TCONS_00015082 TCONS_00072472 TCONS_00072473 TCONS_00049455 TCONS_00090836 TCONS_00078701

TCONS_00056134 TCONS_00004962 TCONS_00065215 TCONS_00088845 TCONS_00086726 TCONS_00057786 TCONS_00059352 TCONS_00002503 TCONS_00011582

TCONS_00018714 TCONS_00080209 TCONS_00082007 TCONS_00050720 TCONS_00052949 TCONS_00083838 TCONS_00048143 TCONS_00007555 TCONS_00081685

TCONS_00043713 TCONS_00075360 TCONS_00089587 TCONS_00069015 TCONS_00086291 TCONS_00073295 TCONS_00079465 TCONS_00071144 TCONS_00070297

TCONS_00074125 TCONS_00004500 TCONS_00084812 TCONS_00008054 TCONS_00011376 TCONS_00086469 TCONS_00088170 TCONS_00003407 TCONS_00081416

TCONS_00091062 TCONS_00020077 TCONS_00084826 TCONS_00024540 TCONS_00010709 TCONS_00086734 TCONS_00036322 TCONS_00061318 TCONS_00074088

TCONS_00010670 TCONS_00021733 TCONS_00051675 TCONS_00057788 TCONS_00033558 TCONS_00013602 TCONS_00067218 TCONS_00033342 TCONS_00084801

TCONS_00046004 TCONS_00052874 TCONS_00012544 TCONS_00021528 TCONS_00021989 TCONS_00033511 TCONS_00046901 TCONS_00078381 TCONS_00033942

TCONS_00077818 TCONS_00082407 TCONS_00080474 TCONS_00078694 TCONS_00042698 TCONS_00082570 TCONS_00069016 TCONS_00002698 TCONS_00020698

TCONS_00053945 TCONS_00000362 TCONS_00074096 TCONS_00002448 TCONS_00012538 TCONS_00089537 TCONS_00090296 TCONS_00090175 TCONS_00031446

TCONS_00012539 TCONS_00012395 TCONS_00027751 TCONS_00030746 TCONS_00064438 TCONS_00081417 TCONS_00089202 TCONS_00002502 TCONS_00022295

TCONS_00019925 TCONS_00084795 TCONS_00056821 TCONS_00042814 TCONS_00042815 TCONS_00056826 TCONS_00000500 TCONS_00041406 TCONS_00014731

TCONS_00030438 TCONS_00010847 TCONS_00010671 TCONS_00029407 TCONS_00088641 TCONS_00029408 TCONS_00032362 TCONS_00064851 TCONS_00029517

TCONS_00015892 TCONS_00007101 TCONS_00042401 TCONS_00042611 TCONS_00091862 TCONS_00056680 TCONS_00080369 TCONS_00029410 TCONS_00064159

TCONS_00065181 TCONS_00021631 TCONS_00090879 TCONS_00041456 TCONS_00029411 TCONS_00057445 TCONS_00071934 TCONS_00049147 TCONS_00011978

TCONS_00038094 TCONS_00041455 TCONS_00042696 TCONS_00045127 TCONS_00014732 TCONS_00011229 TCONS_00002454 TCONS_00068271 TCONS_00068272

TCONS_00079352 TCONS_00039462 TCONS_00005081 TCONS_00034095 TCONS_00087855 TCONS_00080207 TCONS_00084741 TCONS_00012104 TCONS_00056823

TCONS_00002684 TCONS_00015292 TCONS_00048978 TCONS_00051308 TCONS_00054192 TCONS_00057126 TCONS_00057127 TCONS_00065399 TCONS_00088168

TCONS_00003409 TCONS_00084742 TCONS_00071933 TCONS_00083199 TCONS_00074827 TCONS_00067776 TCONS_00064850 TCONS_00030035 TCONS_00018674
